# Artemyrianins A–G from *Artemisia myriantha* and Their Cytotoxicity Against HepG2 Cells

**DOI:** 10.1007/s13659-020-00255-z

**Published:** 2020-06-28

**Authors:** Shuang Tang, Yun-Bao Ma, Chang-An Geng, Cheng Shen, Tian-Ze Li, Xue-Mei Zhang, Li-Hua Su, Zhen Gao, Jing Hu, Ji-Jun Chen

**Affiliations:** 1 State Key Laboratory of Phytochemistry and Plant Resources in West China, Yunnan Key Laboratory of Natural Medicinal Chemistry, Kunming Institute of Botany, Chinese Academy of Sciences, Kunming, 650201 People’s Republic of China; 2 University of Chinese Academy of Sciences, Beijing, 100049 People’s Republic of China

**Keywords:** *Artemisia myriantha*, Artemyrianins A–G, Sesquiterpenoids, Norlignans, Cytotoxicity, HepG2 cells

## Abstract

**Abstract:**

Four new sesquiterpenoids, artemyrianins A–D (**1**–**4**), and three new norlignans, artemyrianins E–G (**5**–**7**), together with five known compounds (**8**–**12**), were isolated from the aerial parts of *Artemisia myriantha* (Asteraceae). The new compounds were established by spectroscopic data analyses (HRMS, IR, 1D and 2D NMR), and their absolute configurations were confirmed by the single-crystal X-ray diffraction or ECD calculations. The isolates showed cytotoxicity against HepG2 cells with IC_50_ values ranging from 33.3 to 145.2 *μ*M.

**Graphic Abstract:**

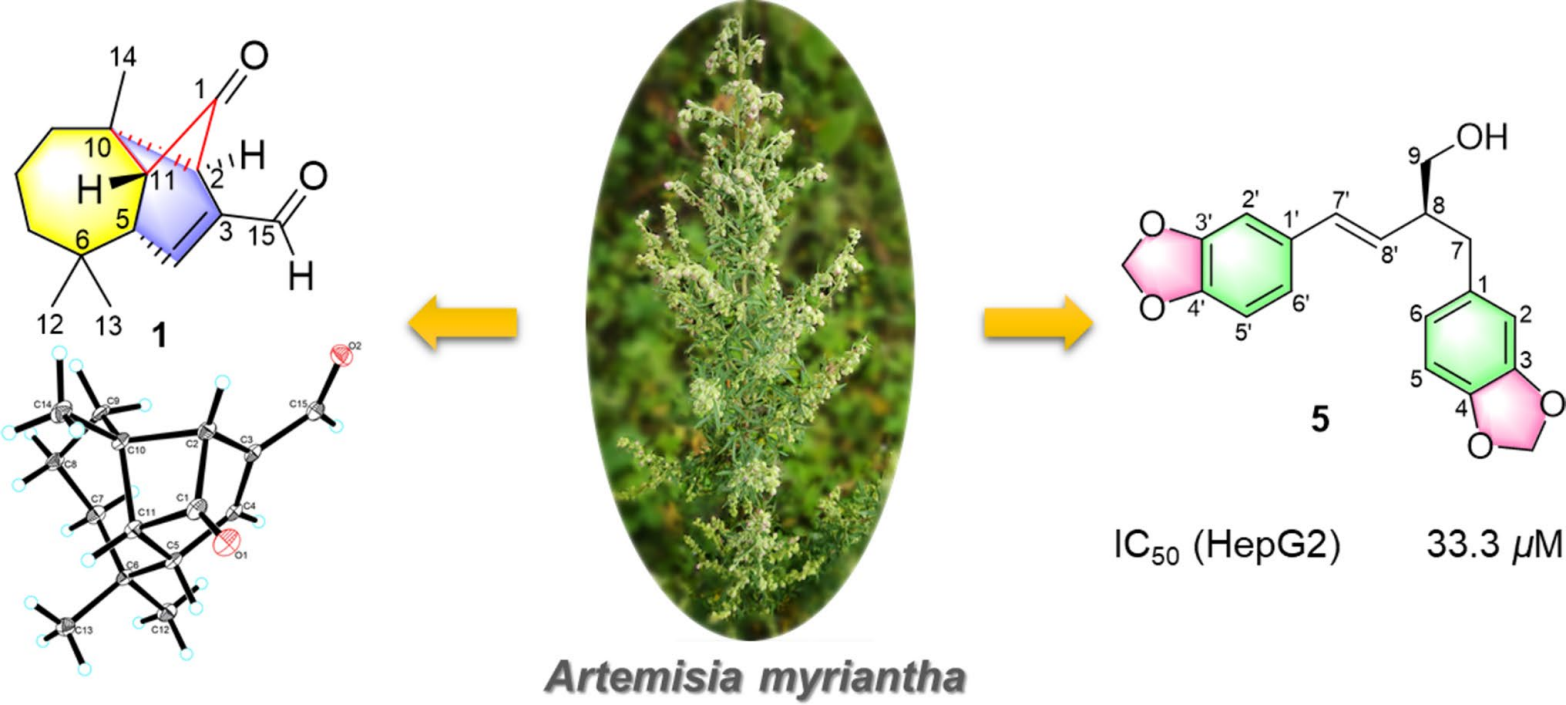

**Electronic supplementary material:**

The online version of this article (10.1007/s13659-020-00255-z) contains supplementary material, which is available to authorized users.

## Introduction

Hepatocellular carcinoma (HCC) resulting in 780000 deaths every year is the fourth cause of cancer mortality [1]. The treatment for advanced HCC has long been unsatisfied until the authorization of sorafenib in 2007. Afterwards, six drugs involving nivolumab, regorafenib, lenvatinib, pembrolizumab, ramucirumab and cabozantinib have been approved by the Food and Drug Administration (FDA). Whereas, the drug resistance and side effects are inevitable [2]. Natural products, especially sesquiterpenoids, such as artemisinin, thapsigargin, parthenolide [3] and arglabin [4] are leading compounds for the development of potent therapeutic agents for neoplastic diseases, which inspired us to search for anti-HCC active compounds with high efficiency and low toxicity from natural sources.

**Fig. 1:**
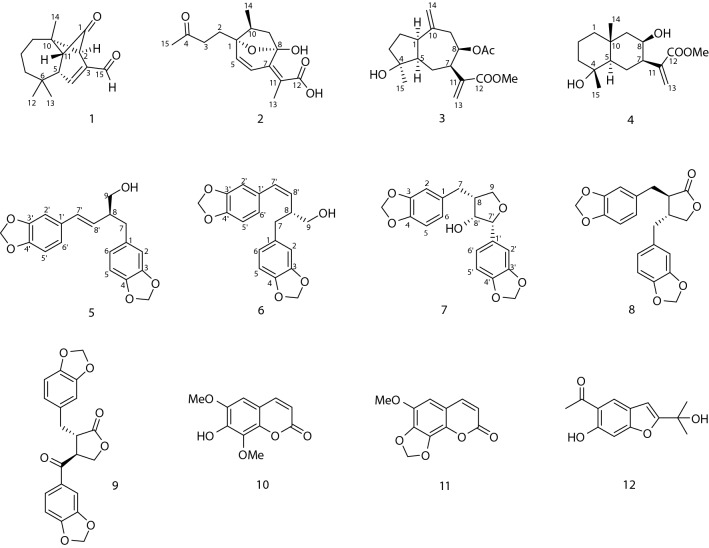
Chemical structures of compounds **1**–**12**

*Artemisia myriantha* Wall. ex Bess., a folk medicine herb, has been used to treat inflammation and menorrhagia diseases [5]. According to previous investigation, sesquiterpenoids [5–9], dimeric sesquiterpenoids [5], flavones [7] and volatile oils [10–12] with antibacterial, anticancer and immunomodulating activity have been reported from this plant. In order to investigate the bioactive constituents of *A. myriantha*, our phytochemical study led to the isolation of four new sesquiterpenoids, artemyrianins A–D (**1**–**4**), three new norlignans, artemyrianins E–G (**5**–**7**), as well as five known compounds (Fig. 1). Herein, we reported their isolation, structural identification and cytotoxicity on HepG2 cells.

## Results and Discussion

**Fig. 2:**
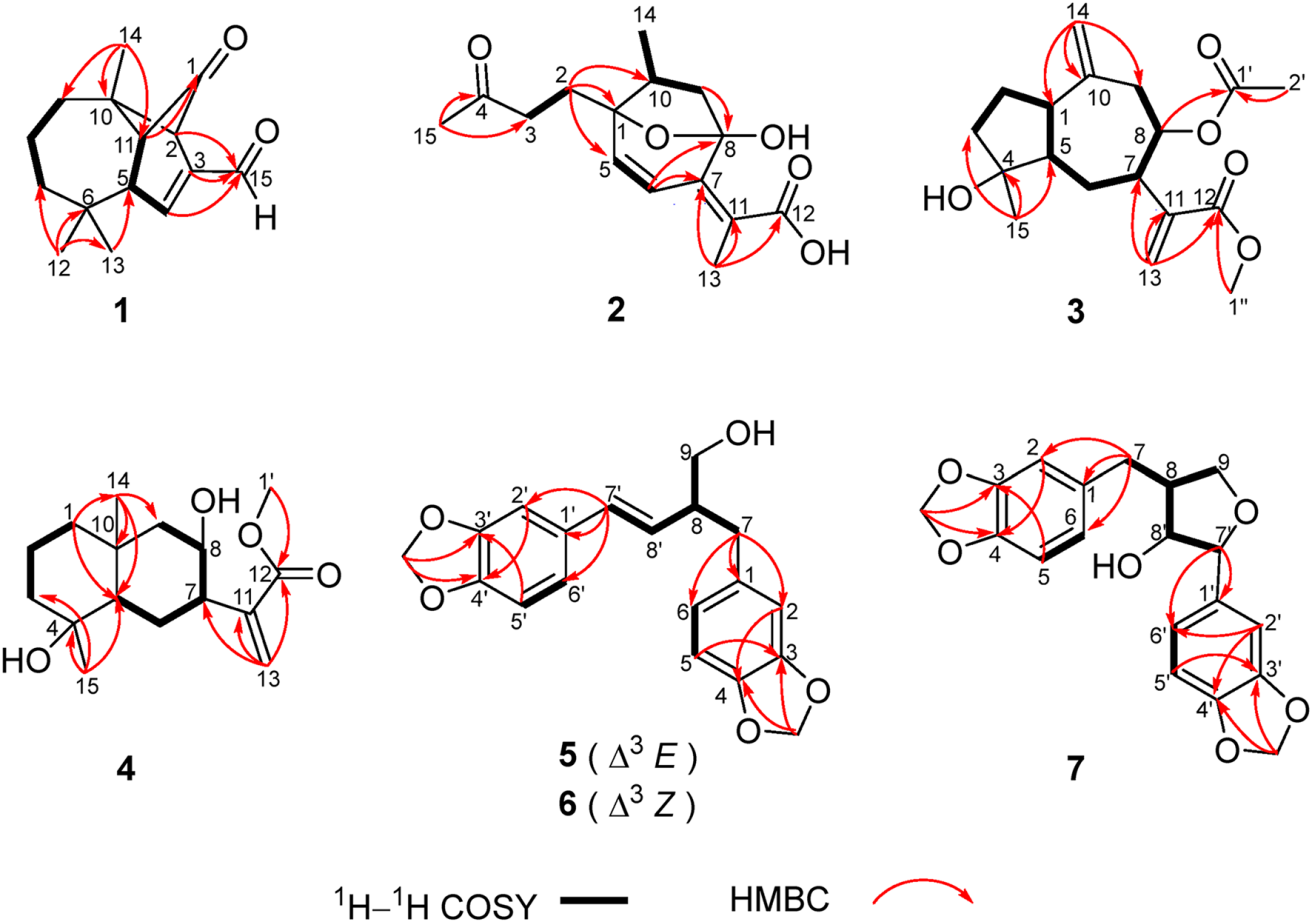
Key ^1^H–^1^H COSY and HMBC correlations of compounds **1**–**7**

**Fig. 3:**
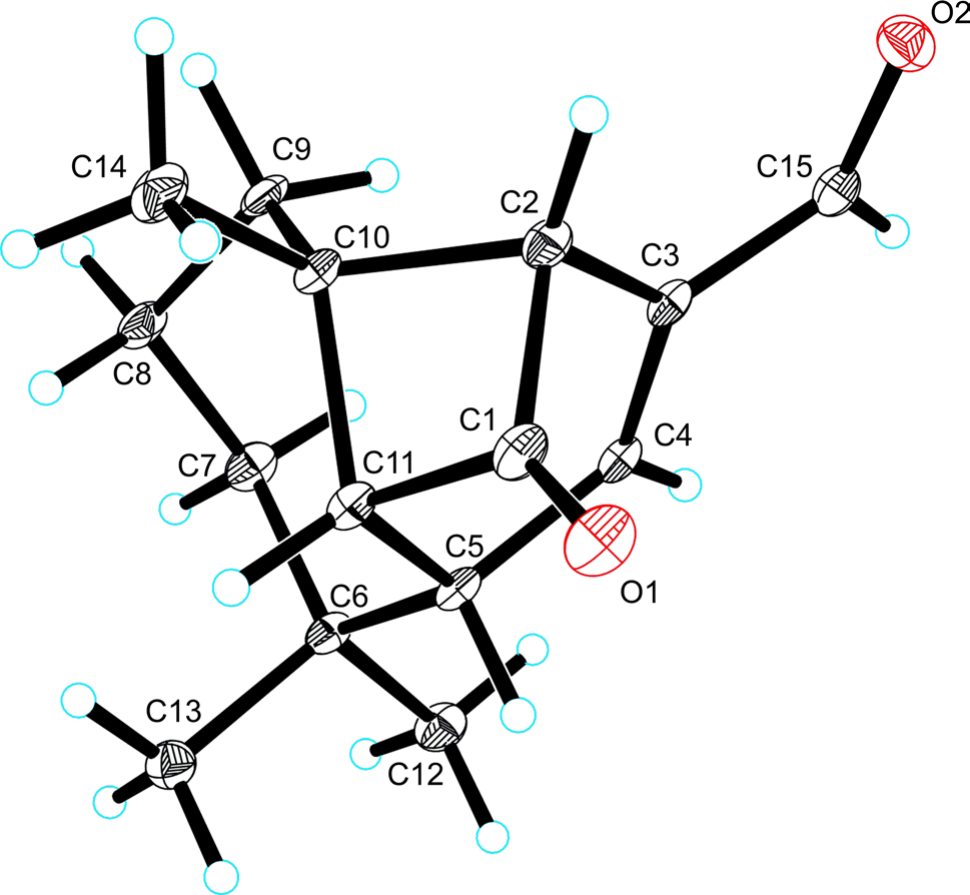
The X-ray ORTEP drawing of compound **1**

Artemyrianin A (**1**) was obtained as colorless prismatic crystals and had a molecular formula of C_15_H_20_O_2_ with six indices of hydrogen deficiency from the (+)-HRESIMS (*m*/*z* 233.1518 [M+H]^+^, calcd for 233.1536). The IR spectrum indicated the presence of carbonyl (1771 and 1671 cm^−1^) and double bond (1606 cm^−1^) functionalities. The ^1^H NMR data (Table 1) exhibited signals for three singlet methyls at *δ*_H_ 1.28 (H_3_-14), 1.16 (H_3_-13) and 1.01 (H_3_-12), a double bond at *δ*_H_ 7.00 (1H, d, *J* = 2.2 Hz, H-4) and a formyl group at *δ*_H_ 9.54 (1H, s, H-15). The ^13^C NMR (DEPT) spectrum displayed 15 carbon resonances, including three methyls at *δ*_C_ 30.5 (C-13), 27.5 (C-14) and 26.5 (C-12), three methylenes at *δ*_C_ 43.2 (C-7), 33.8 (C-9) and 21.6 (C-8), four methines [including an olefinic at *δ*_C_ 148.7 (C-4)], three quaternary carbons [including an olefinic at *δ*_C_ 145.5 (C-3)], a formyl group at *δ*_C_ 189.9 (C-15) and a keto carbonyl at *δ*_C_ 203.9 (C-1). Inspection of its ^1^H and ^13^C NMR (DEPT) data suggested the similar structure with vulgarone A [13] except that a methyl (*δ*_H_ 1.78, *δ*_C_ 23.5) in vulgarone A was replaced by a formyl group (*δ*_H_ 9.54, *δ*_C_ 189.9) in **1**. The HMBC correlations (Fig. 2) from H-15 to C-2 (*δ*_C_ 61.2), C-3 and C-4 confirmed the position of the formyl group at C-3. The single-crystal X-ray diffraction experiment using Cu *Kα* radiation (Fig. 3) unambiguously determined its structure as 3*E*-(2*R*,5*S*,10*S*,11*R*)-15-oxovulgarone A.

**Table 1:** ^1^H NMR and ^13^C NMR (DEPT) data for compounds **1**–**4** (*δ* in ppm, *J* in Hz)

No.	**1**		**2**		**3**		**4**	
	*δ*_C_	*δ*_H_	*δ*_C_	*δ*_H_	*δ*_C_	*δ*_H_	*δ*_C_	*δ*_H_
1	203.9, C	–	87.2, C	–	46.9, CH	3.17, m	41.3, CH_2_	Ha: 1.45, ol^*a*^
	–	–	–	–	–	–	–	Hb: 1.08, td (12.6, 5.0)
2	61.2, CH	3.68, d (7.4)	28.9, CH_2_	Ha: 2.24, ddd (14.9, 9.8, 5.2)	27.0, CH_2_	Ha: 1.96, ol^*a*^	19.7, CH_2_	1.57, m
	–	–	–	Hb: 1.94, m	–	Hb: 1.76, ol^*a*^	–	–
3	145.5, C	–	38.1, CH_2_	Ha: 2.70, ddd (18.2, 9.8, 5.6)	40.3, CH_2_	1.76, m	43.6, CH_2_	Ha: 1.83, m
	–	–	–	Hb: 2.53, ddd (18.2, 9.7, 5.2)	–	–	–	Hb: 1.37, m
4	148.7, CH	7.00, d (2.2)	207.8, C	–	81.8, C	–	72.4, C	–
5	57.1, CH	2.75, q (2.2)	142.6, CH	6.19, d (9.5)	54.1, CH	2.03, ddd (12.1, 9.5, 2.5)	56.0, CH	1.36, ol^*a*^
6	38.2, C	–	119.5, CH	6.59, d (9.5)	26.5, CH_2_	Ha: 1.66, m	20.1, CH_2_	1.69, m
	–	–	–	–	–	Hb: 1.44, m	–	–
7	43.2, CH_2_	Ha: 1.54, ol^*a*^	155.7, C	–	44.3, CH	2.95, m	45.7, CH	2.86, m
	–	Hb: 1.43, m	–	–	–	–	–	–
8	21.6, CH_2_	Ha: 1.55, ol^*a*^	106.4, C	–	70.7, CH	5.09, dt (6.0, 2.8)	68.0, CH	4.06, m
	–	Hb: 1.35, m	–	–	–	–	–	–
9	33.8, CH_2_	1.65, m	38.7, CH_2_	Ha: 2.60, dd (13.2, 10.6)	40.7, CH_2_	Ha: 2.72, dd (13.9, 6.0)	49.9, CH_2_	Ha: 1.81, ol^*a*^
	–	–	–	Hb: 1.63, m	–	Hb: 2.31, dd (13.9, 2.8)	–	Hb: 1.46, ol^*a*^
10	35.4, C	–	41.7, CH	2.42, dp (10.6, 7.0)	144.9, C	–	34.8, C	–
11	65.7, CH	2.99, dt (7.4, 2.2)	117.2, C	–	142.9, C	–	142.0, C	–
12	26.5, CH_3_	1.01, s	172.0, C	–	167.3, C	–	167.7, C	–
13	30.5, CH_3_	1.16, s	8.2, CH_3_	1.89, s	125.8, CH_2_	Ha: 6.26, s	126.5, CH_2_	Ha: 6.39, d (1.2)
	–	–	–	–	–	Hb: 5.53, s	–	Hb: 5.73, t (1.2)
14	27.5, CH_3_	1.28, s	15.5, CH_3_	1.01, d (7.0)	114.0, CH_2_	Ha: 4.91, s	21.1, CH_3_	1.12, s
	–	–	–	–	–	Hb: 4.78, s	–	–
15	189.9, CH	9.54, s	30.0, CH_3_	2.15, s	24.5, CH_3_	1.23, s	23.0, CH_3_	1.20, s
1′	–	–	–	–	170.6, C	–	52.2, CH_3_	3.78, s
2′	–	–	–	–	21.3, CH_3_	1.98, s	–	–
1″	–	–	–	–	52.2, CH_3_	3.76, s	–	–

^*a*^ “ol” is used to indicate overlapped signals, for which the coupling constants could not be read

**Fig. 4:**
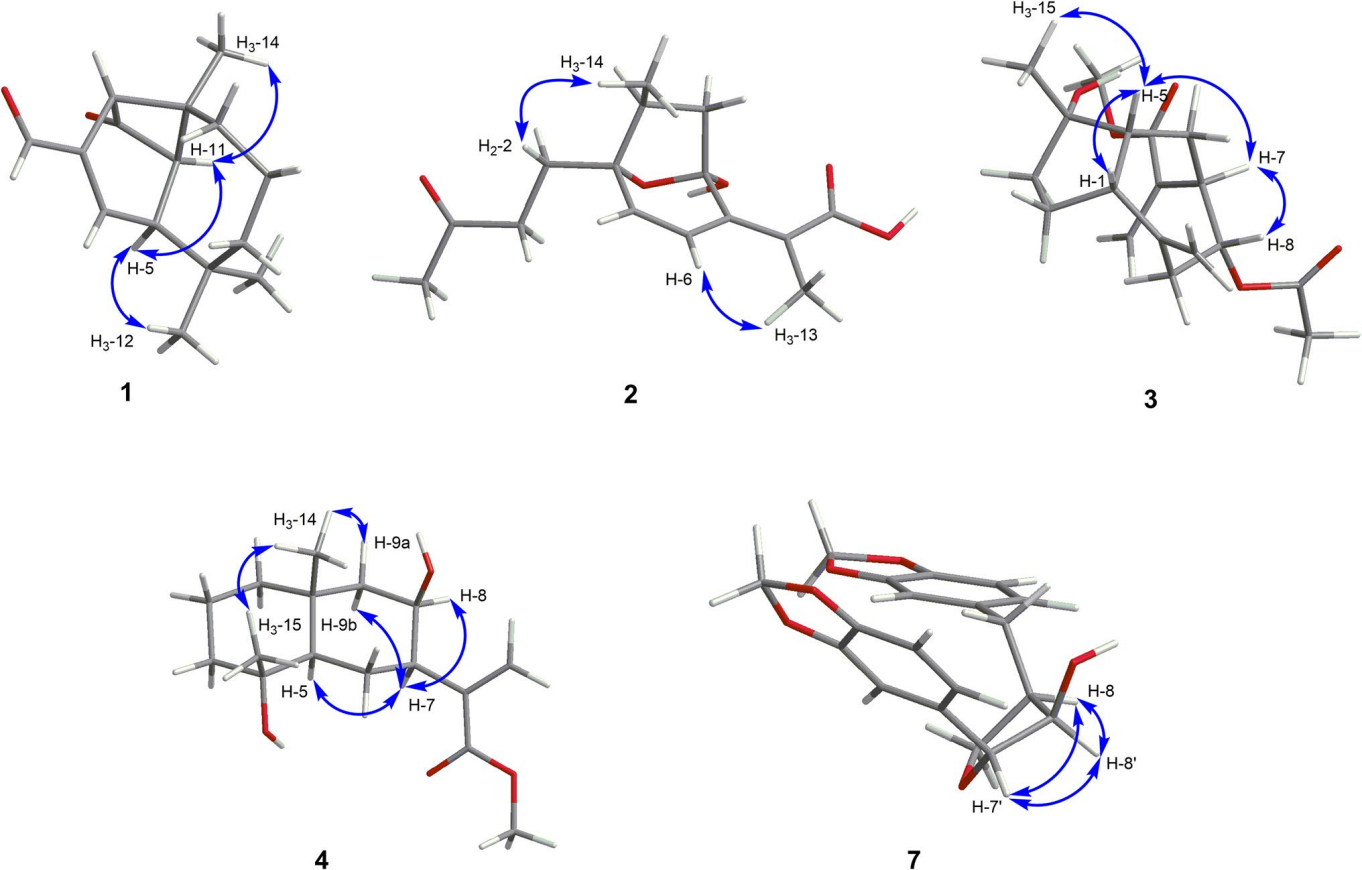
Key ROESY correlations of compounds **1**–**4** and **7**

**Fig. 5:**
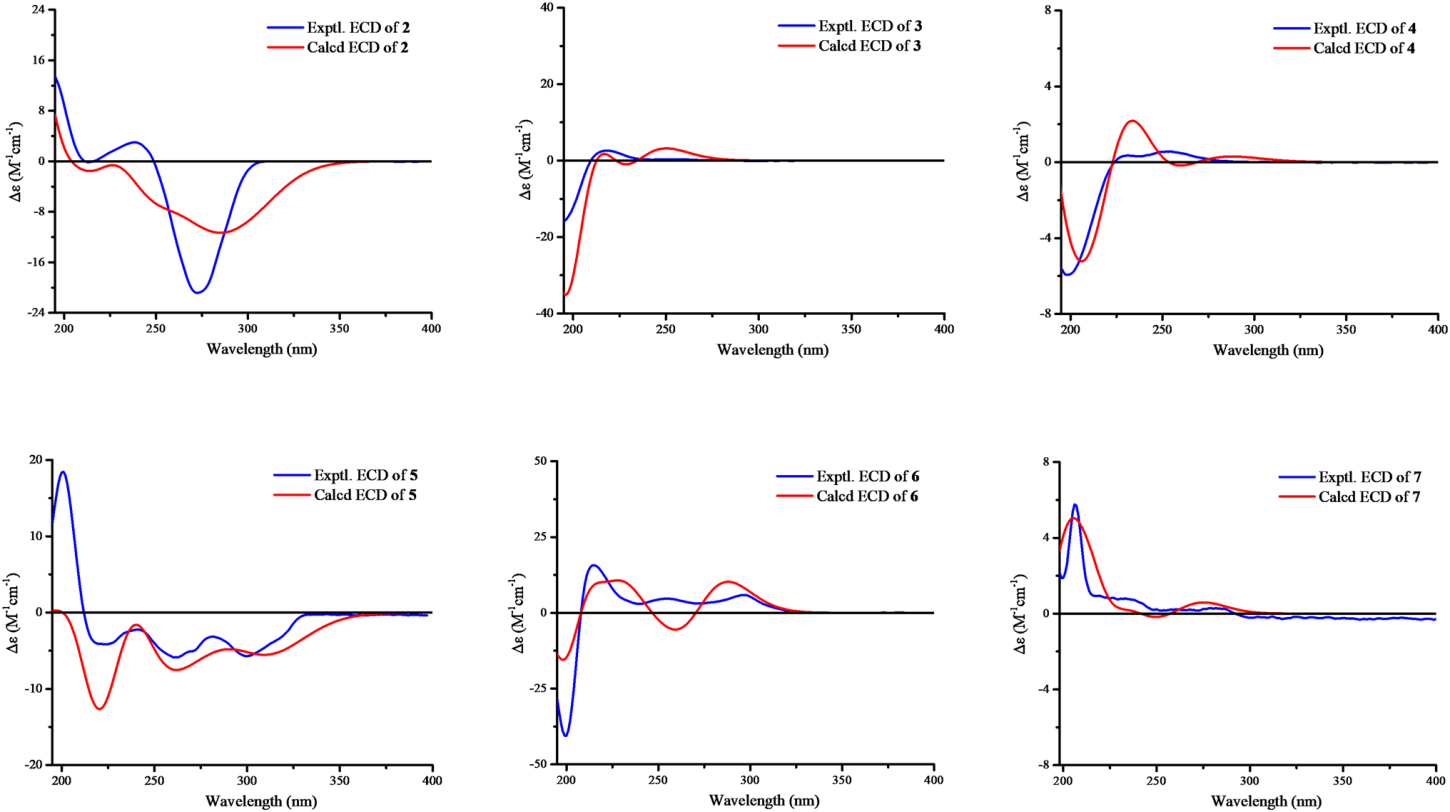
The experimental and calculated ECD spectra of compounds **2**–**7**

Artemyrianin B (**2**) was purified as a white amorphous powder with a molecular formula of C_15_H_20_O_5_ by the [M−H]^−^ ion at *m*/*z* 279.1242 (calcd for 279.1238) in the (−)-HRESIMS, exhibiting six degrees of unsaturation. The absorption bands at 3435, 1771, 1716 and 1633 cm^−1^ in the IR spectrum explained the existence of hydroxy, carbonyl and double bond groups. Two olefinic protons at *δ*_H_ 6.59 (d, *J* = 9.5 Hz, H-6) and 6.19 (d, *J* = 9.5 Hz, H-5), along with three methyls at *δ*_H_ 2.15 (s, H_3_-15), 1.89 (s, H_3_-13) and 1.01 (d, *J* = 7.0 Hz, H_3_-14) were recognized in the ^1^H NMR data. The ^13^C NMR (DEPT) data showed 15 resonances ascribed to three methyls at *δ*_C_ 30.0 (C-15), 15.5 (C-14) and 8.2 (C-13), three methylenes at *δ*_C_ 38.7 (C-9), 38.1 (C-3) and 28.9 (C-2), three methines [including two olefinic at *δ*_C_ 142.6 (C-5) and 119.5 (C-6)], an oxygenated tertiary carbon at *δ*_C_ 87.2 (C-1), a dioxygenated secondary carbon at *δ*_C_ 106.4 (C-8), two olefinic quaternary carbons at *δ*_C_ 155.7 (C-7) and 117.2 (C-11), and two carbonyls at *δ*_C_ 207.8 (C-4) and 172.0 (C-12), indicating a sesquiterpenoid scaffold. The spin systems of H_2_-2/H_2_-3, H-5/H-6 and H_2_-9/H-10/H_3_-14 inferred from the ^1^H–^1^H COSY spectrum, together with the HMBC correlations from H_3_-15 to C-3 and C-4, from H_3_-13 to C-7, C-11 and C-12, from H-6 to C-7 and C-8, from H_2_-9 (*δ*_H_ 2.60, 1.63) to C-8, and from H_2_-2 (*δ*_H_ 2.24, 1.94) to C-1, C-5 and C-10 (*δ*_C_ 41.7) constructed its planar structure with a 7-membered ring. An oxygen bridge linking C-1 and C-8 was affirmed by the de-shielded shifts of C-1 (*δ*_C_ 87.2) and C-8 (*δ*_C_ 106.4), and thus fulfilled the unsaturation degrees. The small coupling constant between H-5 and H-6 (*J* = 9.5 Hz) indicated the *cis*-configuration of Δ^5^. The *cis*-configuration of Δ^7(11)^ was deduced with the fact that the cross-peak between H-6 and H_3_-13 was observed in the ROESY spectrum. In addition, the ROESY correlations (Fig. 4) of H_2_-2 (*δ*_H_ 2.24, 1.94) with H_3_-14 (*δ*_H_ 1.01) revealed their homolateral orientation. Its absolute configuration was established by the electronic circular dichroism (ECD) experiment which fitted well with the calculated ECD curve (Fig. 5). Therefore, compound **2** was characterized as 5*Z*,7(11)*Z*-(1*R*,8*S*,10*S*)-1,8-epoxy-8-hydroxy-4,5-secoguaian-5,7(11)-dien-12-oic acid.

Artemyrianin C (**3**) was assigned a molecular formula of C_18_H_26_O_5_ by the analysis of the (+)-HRESIMS (*m*/*z* 345.1675 [M+Na]^+^, calcd for 345.1672), with six double bond equivalents. The characteristic absorption bands at 3434 (hydroxy), 1735 and 1720 (carbonyl) and 1629 cm^−1^ (double bond) were recognized in the IR spectrum. Compared with 2*α*-acetoxy-4*α*-hydroxy-1*β*-guai-11(13),10(14)-dien-12,8*α*-olide [14], the acetoxy group at C-2 was absent and the *α*-methylene-*γ*-lactone ring was opened in **3**, which connected to a methoxy [*δ*_H_ 3.76 (s, H_3_-1″) and *δ*_C_ 52.2 (C-1″)] and an extra acetyl group [*δ*_H_ 1.98 (s, H_3_-2′) and *δ*_C_ 170.6 (C-1′), 21.3 (C-2′)]. The HMBC correlations from H-8 (*δ*_H_ 5.09) to C-1′, and from H_3_-1″ to C-12 (*δ*_C_ 167.3) verified the acetyl group at C-8 and the presence of methyl ester in the structure. Moreover, the shielded shift of C-2 from *δ*_C_ 75.6 (CH) in 2*α*-acetoxy-4*α*-hydroxy-1*β*-guai-11(13),10(14)-dien-12,8*α*-olide to *δ*_C_ 27.0 (CH_2_) in **3** interpreted the absence of the acetoxy group in **3**. In the ROESY experiment, the correlations of H-5 with H-1, H-7 and H_3_-15, and of H-7 with H-8 explained their same orientation. Its absolute configuration was proposed as 1*R*,4*S*,5*R*,7*R*,8*R* by means of ECD calculation. Consequently, compound **3** was defined as (1*R*,4*S*,5*R*,7*R*,8*R*)-4-hydroxy-8-acetoxyguaian-10(14),11(13)-dien-12-oic acid methyl ester.

Artemyrianin D (**4**) was proposed a molecular formula of C_16_H_26_O_4_ based on the (+)-HRESIMS (*m*/*z* 305.1725 [M+Na]^+^, calcd for 305.1723). The IR spectrum suggested absorption bands for hydroxy, carbonyl and double bond functionalities at 3501, 3459, 1701 and 1626 cm^−1^. Its ^1^H and ^13^C NMR (DEPT) data exhibited similarity with those of 8-desacyl-4*β-*hydroxy-15-dihydrocarmanin [15] except for the absence of three hydroxyl groups in **4**, which was proved by the shielded shifts of C-1 [from *δ*_C_ 76.1 (CH) to *δ*_C_ 41.3 (CH_2_)], C-6 [from *δ*_C_ 66.3 (CH) to *δ*_C_ 20.1 (CH_2_)] and C-15 [from *δ*_C_ 63.7 (CH_2_) to *δ*_C_ 23.0 (CH_3_)], respectively. The above deduction was further supported by the HMBC correlations of H_2_-1 (*δ*_H_ 1.45, 1.08) with C-3 (*δ*_C_ 43.6), C-5 (*δ*_C_ 56.0) and C-14 (*δ*_C_ 21.1), of H_2_-6 (*δ*_H_ 1.69) with C-8 (*δ*_C_ 68.0), C-10 (*δ*_C_ 34.8) and C-11 (*δ*_C_ 142.0), and of H_3_-15 (*δ*_H_ 1.20) with C-3, C-4 (*δ*_C_ 72.4) and C-5. The ROESY correlations of H-7 with H-5, H-8 and H-9b, and of H_3_-14 with H-9a and H_3_-15 suggested that H-5, H-7 and H-8 were on the same side, while the H_3_-14 and H_3_-15 were on the other side. The absolute configuration of **4** was constructed from the high match of experimental and calculated ECD data. Thus, compound **4** was identified as (4*R*,5*R*,7*R*,8*R*,10*R*)-4,8-dihydroxyeudesma-11(13)-en-12-oic acid methyl ester.

Artemyrianin E (**5**) was isolated as a yellowish oil and had a chemical composition of C_19_H_18_O_5_ with eleven degrees of unsaturation from the HREIMS (*m*/*z* 326.1148 [M]^+^, calcd for 326.1154). The IR spectrum displayed the existence of hydroxy (3384 cm^−1^) and phenyl ring (1606, 1503 and 1444 cm^−1^) groups. The similar ^1^H and ^13^C NMR (DEPT) data (Table 2) of **5** to those of (+)-artemisarin [16] revealed their closely related structures. The large coupling constant of H-7′ with H-8′ ($J_{{7^{\prime},8^{\prime}}}$ = 15.7 Hz) indicated the *E*-configuration of $\Delta^{{7^{\prime}}}$ in **5**, instead of *Z*-configuration in (+)-artemisarin ($J_{{7^{\prime},8^{\prime}}}$ = 11.2 Hz). The absolute stereochemistry of **5** was elucidated by the high agreement between the experimental and calculated ECD spectra. Hence, compound **5** was assigned as 7′*E*-(8*S*)-artemisarin.

**Table 2:** ^1^H NMR and ^13^C NMR (DEPT) data for compounds **5**–**7** (*δ* in ppm, *J* in Hz)

No.	**5**		**6**		**7**	
	*δ*_C_	*δ*_H_	*δ*_C_	*δ*_H_	*δ*_C_	*δ*_H_
1	133.5, C	–	133.4, C	–	134.1, C	–
2	109.6, CH	6.67, d (1.8)	109.8, CH	6.62, ol^*a*^	108.5, CH	6.69, d (1.6)
3	145.9, C	–	147.6, C	–	148.0, C	–
4	147.7, C	–	146.6, C	–	146.1, C	–
5	108.2, CH	6.70, ol^*a*^	108.3, CH	6.70, d (7.9)	109.2, CH	6.72, d (7.9)
6	122.2, CH	6.61, dd (7.9, 1.8)	122.2, CH	6.59, ol^*a*^	121.5, CH	6.64, dd (7.9, 1.6)
7	37.8, CH_2_	Ha: 2.72, dd (13.3, 6.6)	38.1, CH_2_	Ha: 2.70, dd (13.7, 6.3)	31.6, CH_2_	Ha: 2.84, dd (13.9, 7.9)
	–	Hb: 2.63, dd (13.3, 7.3)	–	Hb: 2.55, dd (13.7, 7.8)	–	Hb: 2.65, dd (13.9, 7.7)
8	47.7, CH	2.59, m	42.6, CH	3.13, m	44.6, CH	2.47, m
9	65.3, CH_2_	Ha: 3.63, dd (10.6, 5.0)	66.0, CH_2_	Ha: 3.62, dd (10.6, 5.4)	72.1, CH_2_	Ha: 4.17, t (8.2)
	–	Hb: 3.52, dd (10.6, 7.0)	–	Hb: 3.51, dd (10.6, 7.4)	–	Hb: 3.81, dd (10.2, 8.2)
1′	131.7, C	–	131.3, C	–	135.2, C	–
2′	105.6, CH	6.87, d (1.6)	109.0, CH	6.60, ol^*a*^	106.1, CH	6.79, br s
3′	147.2, C	–	146.1, C	–	147.0, C	–
4′	148.1, C	–	147.7, C	–	148.0, C	–
5′	108.4, CH	6.72, ol^*a*^	108.3, CH	6.72, d (7.7)	108.4, CH	6.76, ol^*a*^
6′	120.9, CH	6.74, dd (8.0, 1.6)	122.3, CH	6.59, ol^*a*^	118.7, CH	6.76, ol^*a*^
7′	132.0, CH	6.32, d (15.7)	132.2, CH	6.52, d (11.6)	88.6, CH	4.87, d (2.0)
8′	129.0, CH	5.88, dd (15.7, 8.0)	132.2, CH	5.37, dd (11.6, 10.5)	79.6, CH	4.05, dd (5.1, 2.0)
OCH_2_O	101.2, CH_2_	5.92, s	101.2, CH_2_	5.94, s	101.2, CH_2_	5.94, s
	100.9, CH_2_	5.90, s	101.0, CH_2_	5.91, s	101.1, CH_2_	5.92, s

^*a*^“ol” is used to indicate overlapped signals, for which the coupling constants could not be read

Artemyrianin F (**6**) gave a molecular formula of C_19_H_18_O_5_ from the HREIMS at *m*/*z* 326.1152 ([M]^+^, calcd for 326.1154). Its IR spectrum presented the characteristic absorption bands of hydroxy (3404 cm^−1^) and phenyl ring (1607, 1503 and 1442 cm^−1^) functionalities. The high resemblance between the ^1^H and ^13^C NMR (DEPT) data of **6** and (+)-artemisarin [16] manifested their same planar structure. The geometry of $\Delta^{{7^{\prime}}}$ was *Z*-form as deduced from the small $J_{{7^{\prime},8^{\prime}}}$ value of 11.6 Hz. The ECD spectrum of **6** was superimposable to the calculated one, compound **6** was thus elucidated as 7′*Z*-(8*R*)-artemisarin.

Artemyrianin G (**7**) had a molecular formula of C_19_H_18_O_6_ based on the HREIMS at *m*/*z* 342.1104 ([M]^+^, calcd for 342.1103). The existence of hydroxy (3406 cm^−1^) and phenyl ring (1608, 1503 and 1443 cm^−1^) groups was recognized in the IR spectrum. Its ^1^H and ^13^C NMR (DEPT) spectroscopic data were close to those of mandshuricol A [17], but the main difference was that C-8′ in **7** was a methine attached to a hydroxy with chemical shift of *δ*_C_ 79.6 and C-8′ in mandshuricol A was a quaternary carbon attached to a hydroxy and a hydroxymethyl group with chemical shift of *δ*_C_ 83.4, which suggested that **7** was de-hydroxymethyl derivative at C-8′ of mandshuricol A. The above deduction was supported by the ^1^H–^1^H COSY correlations of H_2_-9/H-8/H-8′/H-7′. The ROESY correlations of H-8/H-8′, H-7′/H-8′ and H-8/H-7′ suggested the same orientation of H-8, H-7′ and H-8′. Its absolute configuration was proposed owning to the well match between the experimental and calculated ECD curves. Accordingly, compound **7** was established as (8*R*,7′*R*,8′*R*)-8′-dehydroxymethylmandshuricol A.

By comparing their ^1^H and ^13^C NMR (DEPT) spectroscopic data with those reported in literatures, five known compounds were identified as (−)-hinokinin (**8**) [18], (8*R*,8′*R*)-4,4′,5,5′-bis(methylenedioxy)-7′-oxolignano-9,9′-lactone (**9**) [19], isofraxidin (**10**) [20], dracunculin (**11**) [21] and 5-acetyl-6-hydroxy-2-(1-hydroxy-1-methylethyl)benzofuran (**12**) [22], respectively.

In addition to four sesquiterpenoids (**1**–**4**), five lignans (**5**–**9**), two coumarins (**10** and **11**) and a benzofuran (**12**) were isolated from *A. myriantha* for the first time. The isolates were assayed for their cytotoxicity against HepG2 cells with sorafenib as the positive control. As a result (Table 3), all compounds showed cytotoxic activity with IC_50_ values ranging from 33.3 to 145.2 *μ*M.

## Experimental

### General Experimental Procedures

HRESIMS data were obtained from an LCMS-IT-TOF mass spectrometer (Shimadzu, Kyoto, Japan) or a UPLC/Q-TOF mass spectrometer (Agilent Technologies, Santa Clara, USA). HREIMS data were acquired on an Agilent G6230 mass spectrometer (Agilent Technologies, Santa Clara, USA). 1D and 2D NMR spectra were recorded on Avance III HD 400 (Bruker, Bremerhaven, Germany) or III 600 (Bruker, Fällanden, Switzerland) spectrometers with TMS as the internal standard. IR spectra were performed on a NICOLET iS10 spectrometer (Thermo Fisher Scientific, Madison, USA) with KBr pellets or ATR ITX-DIAMOND mode. UV spectra were measured on a Shimadzu UV-2700 spectrophotometer (Shimadzu, Suzhou, China). X-ray crystallographic data with Cu *Kα* radiation were collected on a Bruker D8 QUEST apparatus (Bruker, Karlsruher, Germany). ECD experiments were conducted with an Applied Photophysics Chirascan instrument (Applied Photophysics, Surrey, UK). Optical rotations were obtained on an Autopol VI automatic polarimeter (Rudolph Research Analytical, Hackettstown, NJ, USA). Melting point was acquired on an SGW^®^ X-4B microscopic melting point instrument (Shanghai Precision and Scientific Instrument Co., Ltd., Shanghai, China). Silica gel (200–300 mesh) and Sephadex LH-20 gel (20–50 μm) for column chromatography were purchased from Linyi Haixiang Co., Ltd. (Haixiang, Linyi, China) and Pharmacia Fine Chemical Co., Ltd., (Pharmacia, Uppsala, Sweden), respectively. TLC detection was carried out using silica gel HSGF254 plates (Jiangyou, Chemical Co., Ltd., Yantai, China), and compounds were visualized by heating after spraying with 10% H_2_SO_4_ in EtOH (v/v). MPLC separation was achieved on a Dr-Flash II instrument (Lisure Science Co., Ltd., Suzhou, China) with an MCI gel CHP 20P column (75–150 *μ*m, Mitsubishi Chemical Corporation, Tokyo, Japan). Semi-preparative HPLC purification was performed on a Shimadzu HPLC system equipped with LC-20AR pumps and a model SPD-M20A UV detector (Shimadzu, Kyoto, Japan) using an Agilent Eclipse XDB-C_18_ column (5 *μ*m, 9.4 × 250 mm, Agilent Technologies, Santa Clara, USA).

### Plant Material

The aerial parts of *A. myriantha* Wall. ex Bess. were obtained from Lijiang, Yunnan Province, China, in September 2018, and identified by Dr. Zhuo Zhou (Key Laboratory for Plant Diversity and Biogeography of East Asia, Kunming Institute of Botany, Chinese Academy of Sciences). A voucher specimen (No. 201809AM) was stored in the Laboratory of Antivirus and Natural Medicinal Chemistry, Kunming Institute of Botany, Chinese Academy of Sciences.

### Extraction and Isolation

The air-dried aerial parts of *A. myriantha* (24.7 kg) were smashed and extracted with 90% EtOH for two times at room temperature (each 100 L, four days). The combined extracts were concentrated and suspended in H_2_O, which was extracted with EtOAc. The EtOAc portion (1.2 kg) was fractionated into six fractions A–F (300, 120, 150, 230, 165, 220 g) via silica gel column chromatography (CC) using acetone–petroleum ether (PE) gradient (5:95, 10:90, 20:80, 30:70, 40:60 and 50:50, v/v). Fr. C (150 g) was separated through MPLC on an MCI gel CHP 20P column and eluted with a gradient of H_2_O–MeOH (50:50, 30:70, 20:80 and 0:100) to give four subfractions (Frs. C1–C4). Fr. C1 (29 g) was subjected to silica gel CC (EtOAc–PE, 10:90 to 30:70) to afford three subfractions (Frs. C1-1‒C1-3). Compound **4** (1.9 g) was obtained by recrystallization in MeOH from Fr. C1-1 (12.5 g). Fr. C1-2 (7 g) was chromatographed over Sephadex LH-20 CC (MeOH–CHCl_3_, 50:50) and further purified by semi-preparative HPLC (H_2_O–MeCN, 38:62) to provide compound **2** (6 mg, *t*_R_ = 27.5 min). Fr. C2 (35 g) was separated into three subfractions (Frs. C2-1‒C2-3) via silica gel CC with a gradient of EtOAc–PE (15:85 to 30:70). From Fr. C2-1 (1.5 g), compounds **1** (30 mg, *t*_R_ = 13.0 min) and **12** (8 mg, *t*_R_ = 39.5 min) was obtained by semi-preparative HPLC with H_2_O–MeCN (42:58) as the mobile phase. Fr. C2-3 (12 g) was submitted on silica gel CC with a step gradient of acetone–PE (20:80 to 30:70) and further purified by semi-preparative HPLC (H_2_O–MeOH, 51:49) to yield compounds **3** (62 mg, *t*_R_ = 23.5 min) and **10** (9 mg, *t*_R_ = 35.0 min). Fr. C4 (16.5 g) was separated on silica gel CC (EtOAc–PE, 15:85 to 30:70) to give three subfractions (Frs. C4-1–C4-3). Compounds **5** (85 mg, *t*_R_ = 25.0 min), **6** (15 mg, *t*_R_ = 21.5 min), **8** (7 mg, *t*_R_ = 14.0 min), and **11** (88 mg, *t*_R_ = 32.0 min) were provided from Fr. C4-2 (1.3 g) by semi-preparative HPLC (H_2_O–MeCN, 42:58). Fr. C4-3 (2.7 g) was subjected to Sephadex LH-20 CC (MeOH–CHCl_3_, 50:50) and further purified by semi-preparative HPLC (H_2_O–MeCN, 47:53) to afford compounds **7** (4 mg, *t*_R_ = 19.0 min) and **9** (4 mg, *t*_R_ = 15.0 min).

### Spectroscopy Data of Compounds

The ^1^H NMR spectra of artemyrianins A–G (**1**–**7**) were recorded in CDCl_3_ at 600 MHz, and their ^13^C NMR (DEPT) spectra were recorded in CDCl_3_ at 150 MHz.

#### Artemyrianin A (**1**)

Colorless, prismatic crystals (MeOH); mp 95.8–97.5 °C; [*α*]_D_^22^ + 45 (*c* 0.13, MeOH); UV (MeOH) *λ*_max_ (log *ε*) 206 (3.69), 224 (3.73), 266 (3.49) nm; IR *ν*_max_ 1771, 1671, 1606, 1454, 1132 cm^−1^; ^1^H NMR and ^13^C NMR (DEPT) data see Table 1; (+)-HRESIMS *m*/*z* 233.1518 [M+H]^+^ (calcd for C_15_H_21_O_2_, 233.1536).

#### Artemyrianin B (**2**)

White amorphous powder; [*α*]_D_^22^ − 205 (*c* 0.11, MeOH); UV (MeOH) *λ*_max_ (log *ε*) 263 (3.06) nm; ECD (MeOH) *λ*_max_ (Δ*ε*) 213 (− 0.14), 238 (+ 3.00), 272 (− 20.81) nm; IR (KBr) *ν*_max_ 3435, 1771, 1716, 1633, 1291, 1171, 1085, 1026 cm^−1^; ^1^H NMR and ^13^C NMR (DEPT) data see Table 1; ( −)-HRESIMS *m*/*z* 279.1242 [M−H]^−^ (calcd for C_15_H_19_O_5_, 279.1238).

#### Artemyrianin C (**3**)

White amorphous powder; [*α*]_D_^24^ − 110 (*c* 0.11, MeOH); UV (MeOH) *λ*_max_ (log *ε*) 204 (4.08) nm; ECD (MeOH) *λ*_max_ (Δ*ε*) 219 (+ 2.17) nm; IR (KBr) *ν*_max_ 3434, 1735, 1720, 1629, 1440, 1376, 1247, 1148 cm^−1^; ^1^H NMR and ^13^C NMR (DEPT) data see Table 1; (+)-HRESIMS *m*/*z* 345.1675 [M+Na]^+^ (calcd for C_18_H_26_O_5_Na, 345.1672).

#### Artemyrianin D (**4**)

White amorphous powder; [*α*]_D_^24^ − 111 (*c* 0.16, MeOH); UV (MeOH) *λ*_max_ (log *ε*) 206 (3.82) nm; ECD (MeOH) *λ*_max_ (Δ*ε*) 196 (− 5.96), 254 (+ 0.56) nm; IR (KBr) *ν*_max_ 3501, 3459, 1701, 1626, 1290, 1245 cm^−1^; ^1^H NMR and ^13^C NMR (DEPT) data see Table 1; (+)-HRESIMS *m*/*z* 305.1725 [M+Na]^+^ (calcd for C_16_H_26_O_4_Na, 305.1723).

#### Artemyrianin E (**5**)

Yellowish oil; [*α*]_D_^27^− 185 (*c* 0.22, MeOH); UV (MeOH) *λ*_max_ (log *ε*) 215 (4.18), 267 (4.04), 290 (3.87) nm; ECD (MeCN) *λ*_max_ (Δ*ε*) 201 (+ 18.46), 224 (− 4.18), 241 (− 2.24), 262 (− 5.89), 282 (− 3.15), 300 (− 5.71) nm; IR *ν*_max_ 3384, 1606, 1503, 1489, 1444, 1250, 1190, 1039, 929, 804 cm^−1^; ^1^H NMR and ^13^C NMR (DEPT) data see Table 2; HREIMS *m*/*z* 326.1148 [M]^+^ (calcd for C_19_H_18_O_5_, 326.1154).

#### Artemyrianin F (**6**)

Yellowish oil; [*α*]_D_^19^ + 152 (*c* 0.15, MeOH); UV (MeCN) *λ*_max_ (log *ε*) 262 (3.03), 290 (2.95) nm; ECD (MeCN) *λ*_max_ (Δ*ε*) 200 (− 40.56), 215 (+ 15.69), 240 (+ 2.92), 297 (+ 5.89) nm; IR (KBr) *ν*_max_ 3404, 1607, 1503, 1489, 1442, 1245, 1189, 1040, 928, 869, 812 cm^−1^; ^1^H NMR and ^13^C NMR (DEPT) data see Table 2; HREIMS *m*/*z* 326.1152 [M]^+^ (calcd for C_19_H_18_O_5_, 326.1154).

#### Artemyrianin G (**7**)

White amorphous powder; [*α*]_D_^26^ − 18 (*c* 0.12, MeOH); UV (MeOH) *λ*_max_ (log *ε*) 235 (3.63), 287 (3.59) nm; ECD (MeCN) *λ*_max_ (Δ*ε*) 206 (+ 5.76), 248 (+ 0.20), 282 (+ 0.29) nm; IR *ν*_max_ 3406, 1608, 1503, 1490, 1443, 1247, 1189, 1099, 1039, 927, 811 cm^−1^; ^1^H NMR and ^13^C NMR (DEPT) data see Table 2; HREIMS *m*/*z* 342.1104 [M]^+^ (calcd for C_19_H_18_O_6_, 342.1103).

### X-ray Crystallographic Analysis of Artemyrianin A (**1**)

Single crystals of compound **1** were obtained from MeOH. X-ray crystallographic data were collected on a Bruker D8 Quest apparatus employing graphite-monochromated Cu *Kα* radiation at 100.(2) K. Cell refinement and data reduction were carried out by the aid of Bruker SAINT. The structure was solved by direct methods with SHELXS-97. All non-hydrogen atoms were refined anisotropically by means of the least-squares method, and all hydrogen atoms were positioned using difference Fourier overlapping calculation and geometric calculations. Flack parameter = 0.18(14). The crystallographic data for **1** have been deposited at the Cambridge Crystallographic Data Centre (CCDC) as Supplementary Publication No. CCDC 1985641. Copy of the data can be obtained free of charge from CCDC, 12 Union Road, Cambridge CB2 1EZ, UK [fax: +44 (0) 1223 336033; e-mail: deposit@ccdc.cam.ac.uk].

#### Crystal Data for **1**

C_15_H_20_O_2_, *M* = 232.31, *a* = 6.7424(3) Å, *b* = 6.9858(3) Å, *c* = 7.7309(3) Å, *α* = 105.3440(10)°, *β* = 101.9110(10)°, *γ* = 106.6430(10)°, *V* = 320.37(2) Å^3^, *T* = 100.(2) K, space group *P*1, *Z* = 1, *μ*(Cu *Kα*) = 0.615 mm^−1^, 4616 reflections measured, 2090 independent reflections (*R*_*int*_ = 0.0519). The final *R*_1_ values were 0.0484 [*I* > 2*σ*(*I*)]. The final *wR*(*F*^2^) values were 0.1244 [*I* > 2*σ*(*I*)]. The final *R*_1_ values were 0.0485 (all data). The final *wR*(*F*^2^) values were 0.1245 (all data). The goodness of fit on *F*^2^ was 1.040. Flack parameter = 0.18(14).

### Computational Details

The ECD calculations for compounds **2**–**7** were achieved by Gaussian 09 program. The configurations of compounds **2**–**4** and **7** were initially established on the basis of their ROESY data, and further optimized by means of the DFT calculation at b3lyp/6-31G(d,p) level in the gas phase. The imaginary frequencies were excluded by the aid of frequency calculations at the same level. Taking the solvent effects into consideration, ECD calculations were carried out using the TDDFT methodology at b3lyp/6-311+g(d,p) level. The ECD curves were plotted by the Origin Pro 9 program (OriginLab Corporation, Northampton, USA).

### Cytotoxicity Assay

MTT assays were conducted to evaluate the cytotoxic activity of the isolates on HepG2 cells [23]. HepG2 cells were seeded onto 96-well plates at a density of 1 × 10^4^ cells per well. Following 24 h incubation at 37 °C with 5% CO_2_, cells were treated with 100 *μ*L of culture medium containing tested samples at gradient concentrations for 48 h. Next, the medium was removed and replaced by 100 *μ*L MTT solution (1 mg/mL), and the plates were further incubated in dark for four h. After removing the medium, 100 *μ*L DMSO was added into each well to dissolve the MTT formazan salt. Then the plates were measured at 490 nm on a microplate reader (Bio-Rad, USA). The cytotoxicity of compounds was expressed as IC_50_ values which calculated by GraphPad prism 5 (GraphPad Software, San Diego, California, USA). The HepG2 cells (Jining Biotechnology Co., Ltd., Shanghai, China) were maintained at 37 °C with 5% CO_2_ and grown in Dulbecco’s Modified Eagle Medium (DMEM) (Gibco, Thermo Fisher Scientific Co., Ltd., Suzhou, China) supplemented with 10% fetal bovine serum (FBS) (Gibco, Life Technologies, NY, USA) (Table 3).

**Table 3:** Cytotoxic activity of compounds against HepG2 cells

Compound	IC_50_ ± SD (μM)^*a*^	Compound	IC_50_ ± SD (μM)^*a*^
**1**	117.5 ± 3.8	**8**	76.8 ± 6.5
**2**	119.2 ± 8.3	**9**	126.4 ± 1.5
**3**	145.2 ± 5.3	**10**	134.8 ± 8.0
**4**	55.4 ± 3.8	**11**	133.2 ± 12.6
**5**	33.3 ± 0.6	**12**	47.1 ± 6.1
**6**	66.4 ± 0.7	Sorafenib	9.2 ± 2.7
**7**	96.9 ± 8.1		

^*a*^Data were expressed as means ± SD (*n* = 3) from three independent experiments

## Electronic supplementary material

Below is the link to the electronic supplementary material.

Supplementary file1 (DOCX 7425 kb)
