# Supplementary material for: Artemyrianins A–G from Artemisia myriantha and Their Cytotoxicity Against HepG2 Cells
Source: Nat Prod Bioprospect. 2020 Jun 28;10(4):251–60. doi: 10.1007/s13659-020-00255-z (PMC7367949; doi:10.1007/s13659-020-00255-z)
Supplement: Supplementary file 1 — Supplementary file1 (DOCX 7425 kb) [file 13659_2020_255_MOESM1_ESM.docx]

**Supporting Information**

**Artemyrianins A**−**G from *Artemisia myriantha* and Their Cytotoxicity against HepG2 Cells**

**Shuang Tang^1,2^ ‧ Yun-Bao Ma^1^ ‧ Chang-An Geng^1^ ‧ Cheng Shen^1,2^ ‧ Tian-Ze Li^1^ ‧ Xue-Mei Zhang^1^ ‧ Li-Hua Su^1,2^ ‧ Zhen Gao^1,2^ ‧ Jing Hu^1^ ‧ Ji-Jun Chen** **⃰ ^,1,2^**

^1^State Key Laboratory of Phytochemistry and Plant Resources in West China, Yunnan Key Laboratory of Natural Medicinal Chemistry, Kunming Institute of Botany, Chinese Academy of Sciences, Kunming 650201, People's Republic of China

^2^University of Chinese Academy of Sciences, Beijing 100049, People's Republic of China

*Corresponding author. Kunming Institute of Botany, Chinese Academy of Sciences, Kunming 650201, People's Republic of China. Tel.: +86 871 65223265; Fax: +86-871-65227197. *E-mail address*: [chenjj@mail.kib.ac.cn](mailto:chenjj@mail.kib.ac.cn) (Ji-Jun Chen).

**Contents**

Computational details‧‧‧‧‧‧‧‧‧‧‧‧‧‧‧‧‧‧‧‧‧‧‧‧‧‧‧‧‧‧‧‧‧‧‧‧‧‧‧‧‧‧‧‧‧‧‧‧‧‧‧‧‧‧‧‧‧‧‧‧‧‧‧‧‧‧‧‧‧‧‧‧‧‧‧‧‧‧‧‧‧‧‧‧‧‧‧‧‧‧‧‧‧‧‧‧‧‧‧‧‧‧‧‧‧‧‧‧‧‧‧P3

Figures S1−10, the spectroscopic data of artemyrianin A (**1**)‧‧‧‧‧‧‧‧‧‧‧‧‧‧‧‧‧‧‧‧‧‧‧‧‧‧‧‧‧‧‧‧‧‧‧‧‧‧‧‧‧‧‧‧‧‧P4−8

Figures S11−20, the spectroscopic data of artemyrianin B (**2**)‧‧‧‧‧‧‧‧‧‧‧‧‧‧‧‧‧‧‧‧‧‧‧‧‧‧‧‧‧‧‧‧‧‧‧‧‧‧‧‧‧‧P9−13

Figures S21−30, the spectroscopic data of artemyrianin C (**3**)‧‧‧‧‧‧‧‧‧‧‧‧‧‧‧‧‧‧‧‧‧‧‧‧‧‧‧‧‧‧‧‧‧‧‧‧‧‧‧‧P14−18

Figures S31−40, the spectroscopic data of artemyrianin D (**4**)‧‧‧‧‧‧‧‧‧‧‧‧‧‧‧‧‧‧‧‧‧‧‧‧‧‧‧‧‧‧‧‧‧‧‧‧‧‧‧‧P19−23

Figures S41−51, the spectroscopic data of artemyrianin E (**5**)‧‧‧‧‧‧‧‧‧‧‧‧‧‧‧‧‧‧‧‧‧‧‧‧‧‧‧‧‧‧‧‧‧‧‧‧‧‧‧‧P24−28

Figures S52−62, the spectroscopic data of artemyrianin F (**6**)‧‧‧‧‧‧‧‧‧‧‧‧‧‧‧‧‧‧‧‧‧‧‧‧‧‧‧‧‧‧‧‧‧‧‧‧‧‧‧‧P29−33

Figures S63−73, the spectroscopic data of artemyrianin G (**7**)‧‧‧‧‧‧‧‧‧‧‧‧‧‧‧‧‧‧‧‧‧‧‧‧‧‧‧‧‧‧‧‧‧‧‧‧‧‧‧‧P34−38

X-ray crystallographic data for artemyrianin A (**1**)‧‧‧‧‧‧‧‧‧‧‧‧‧‧‧‧‧‧‧‧‧‧‧‧‧‧‧‧‧‧‧‧‧‧‧‧‧‧‧‧‧‧‧‧‧‧‧‧‧‧‧‧‧‧‧‧P39−40

**Computational details**

The ECD calculations for compounds **2**−**7** were achieved by Gaussian 09 program. The configurations of compounds **2**−**4** and **7** were initially established on the basis of their ROESY data, and further optimized by means of the DFT calculation at b3lyp/6-31G(d,p) level in the gas phase. The imaginary frequencies were excluded by the aid of frequency calculations at the same level. Taking the solvent effects into consideration, ECD calculations were carried out using the TDDFT methodology at b3lyp/6-311+g(d,p) level. The ECD curves were plotted by the Origin Pro 9 program (OriginLab Corporation, Northampton, USA).

1. Frisch, M. J.; Trucks, G. W.; Schlegel, H. B.; Scuseria, G. E.; Robb, M. A.; Cheeseman, J. R.; Scalmani, G.; Barone, V.; Mennucci, B.; Petersson, G. A.; Nakatsuji, H.; Caricato, M.; Li, X.; Hratchian, H. P.; Izmaylov, A. F.; Bloino, J.; Zheng, G.; Sonnenberg, J. L.; Hada, M.; Ehara, M.; Toyota, K.; Fukuda, R.; Hasegawa, J.; Ishida, M.; Nakajima, T.; Honda, Y.; Kitao, O.; Nakai, H.; Vreven, T.; Montgomery, J. A.; Peralta, J. J. E.; Ogliaro, F.; Bearpark, M.; Heyd, J. J.; Brothers, E.; Kudin, K. N.; Staroverov, V. N.; Keith, T.; Kobayashi, R.; Normand, J.; Raghavachari, K.; Rendell, A.; Burant, J. C.; Iyengar, S. S.; Tomasi, J.; Cossi, M.; Rega, N.; Millam, J. M.; Klene, M.; Knox, J. E.; Cross, J. B.; Bakken, V.; Adamo, C.; Jaramillo, J.; Gomperts, R.; Stratmann, R. E.; Yazyev, O.; Austin, A. J.; Cammi, R.; Pomelli, C.; Ochterski, J. W.; Martin, R. L.; Morokuma, K.; Zakrzewski, V. G.; Voth, G. A.; Salvador, P.; Dannenberg, J. J.; Dapprich, S.; Daniels, A. D.; Farkas, O.; Foresman, J. B.; Ortiz, J. V.; Cioslowski, J.; Fox, D. J. Gaussian 09, Revision C.01; Gaussian, Inc., Wallingford CT: 2010.


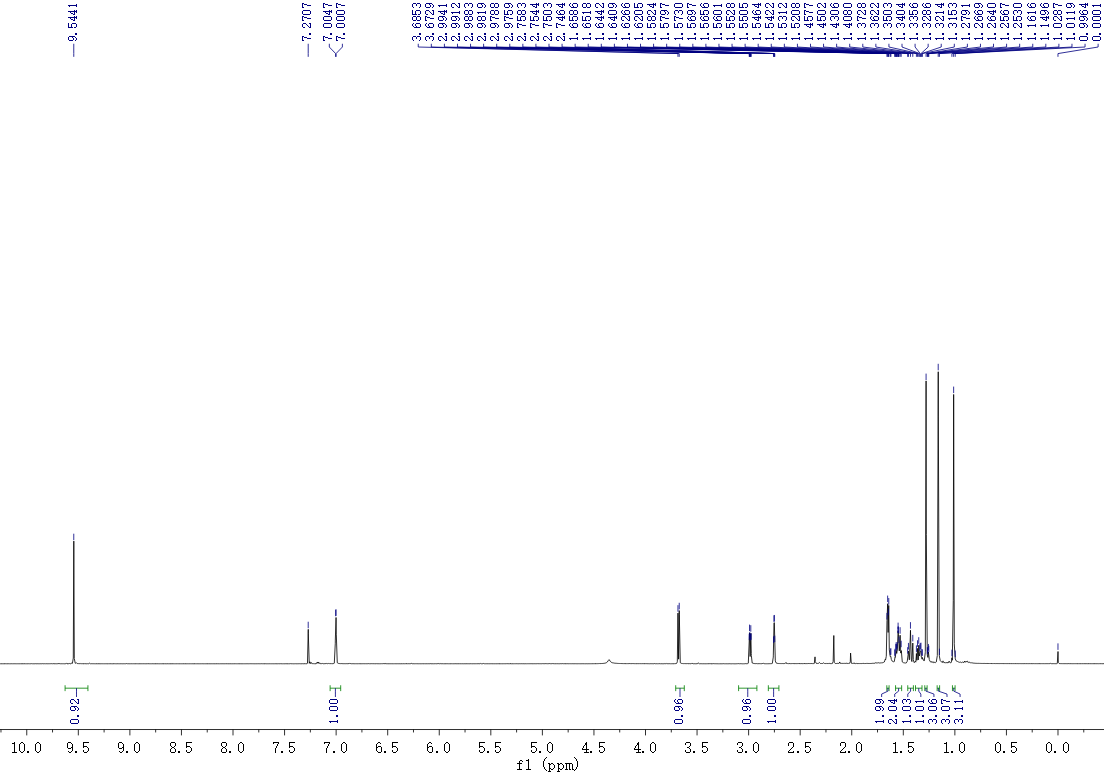


**Figure S1**. ^1^H NMR spectrum of artemyrianin A (**1**) recorded in CDCl_3_ at 600 MHz


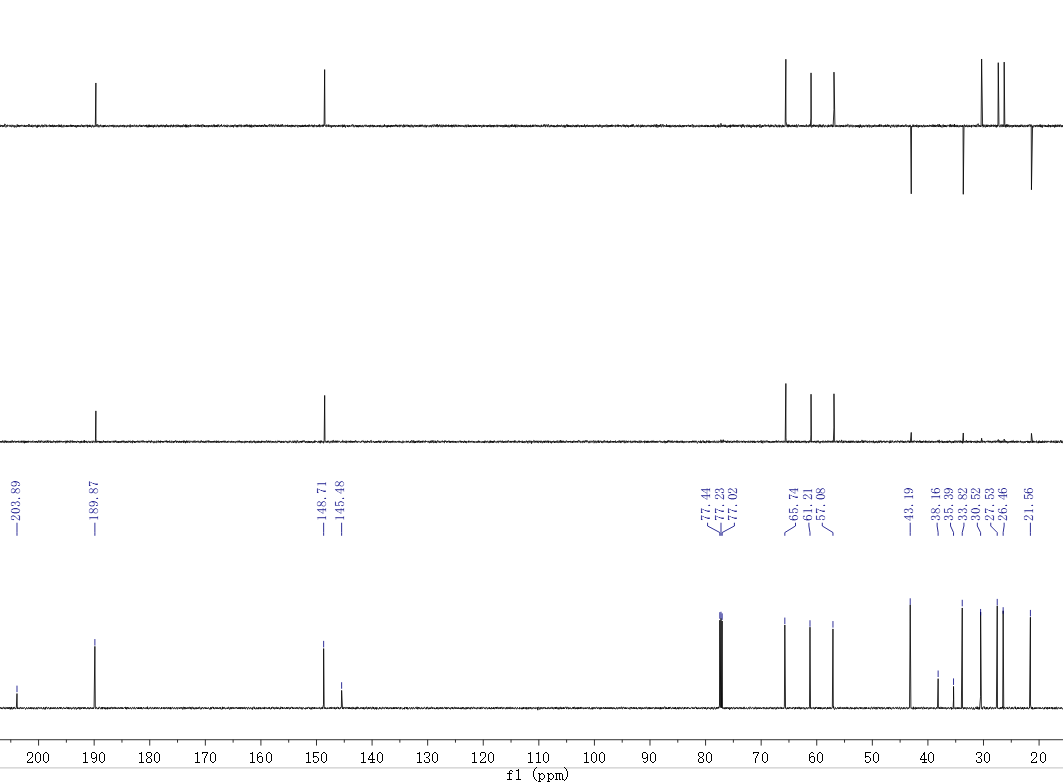


**Figure S2**. ^13^C NMR (DEPT) spectrum of artemyrianin A (**1**) recorded in CDCl_3_ at 150 MHz


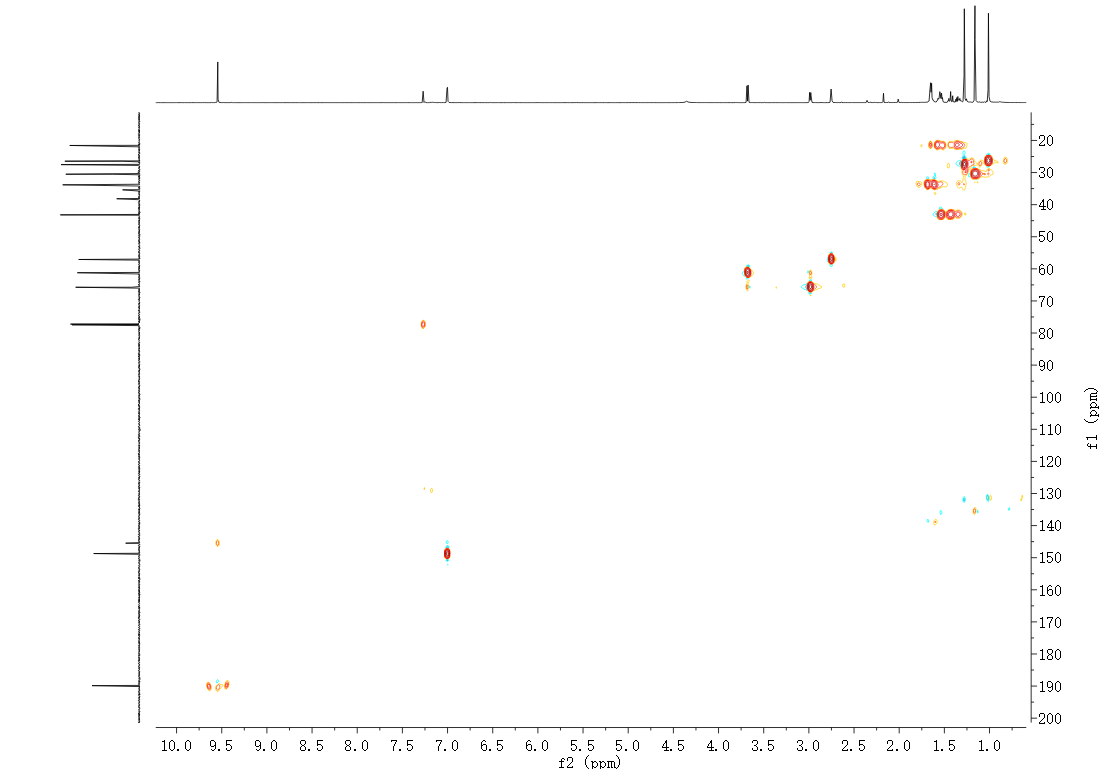


**Figure S3**. HSQC spectrum of artemyrianin A (**1**) recorded in CDCl_3_


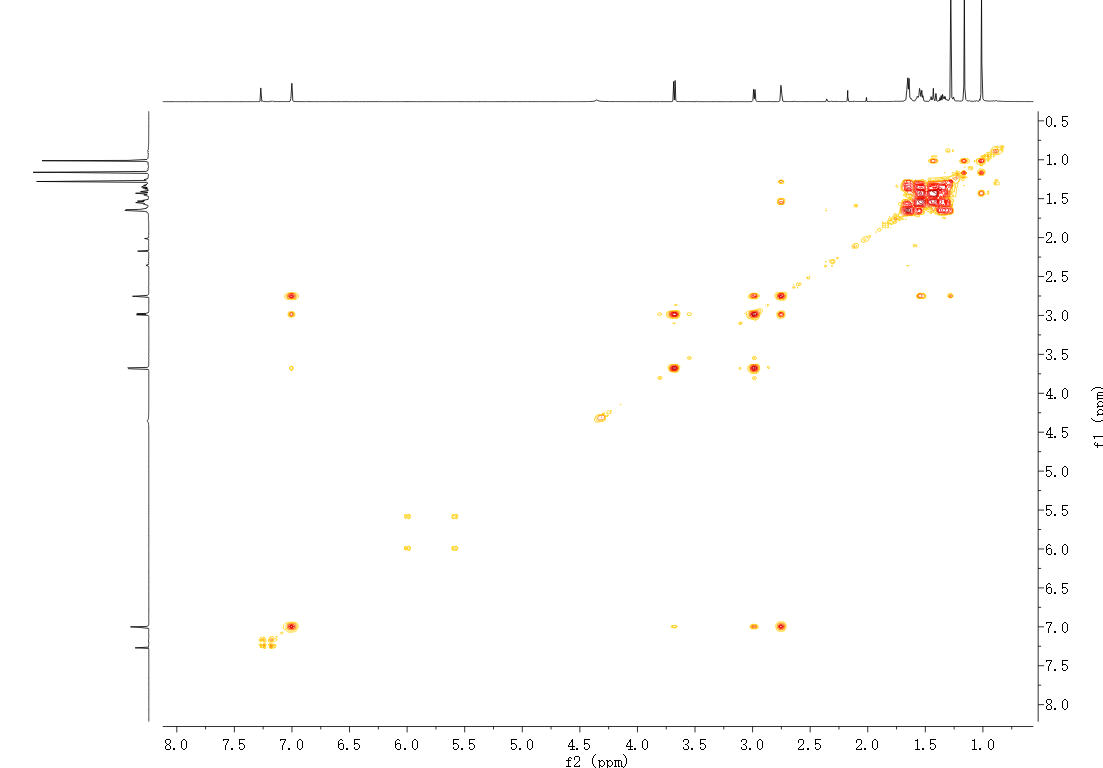


**Figure S4**. ^1^H−^1^H COSY spectrum of artemyrianin A (**1**) recorded in CDCl_3_


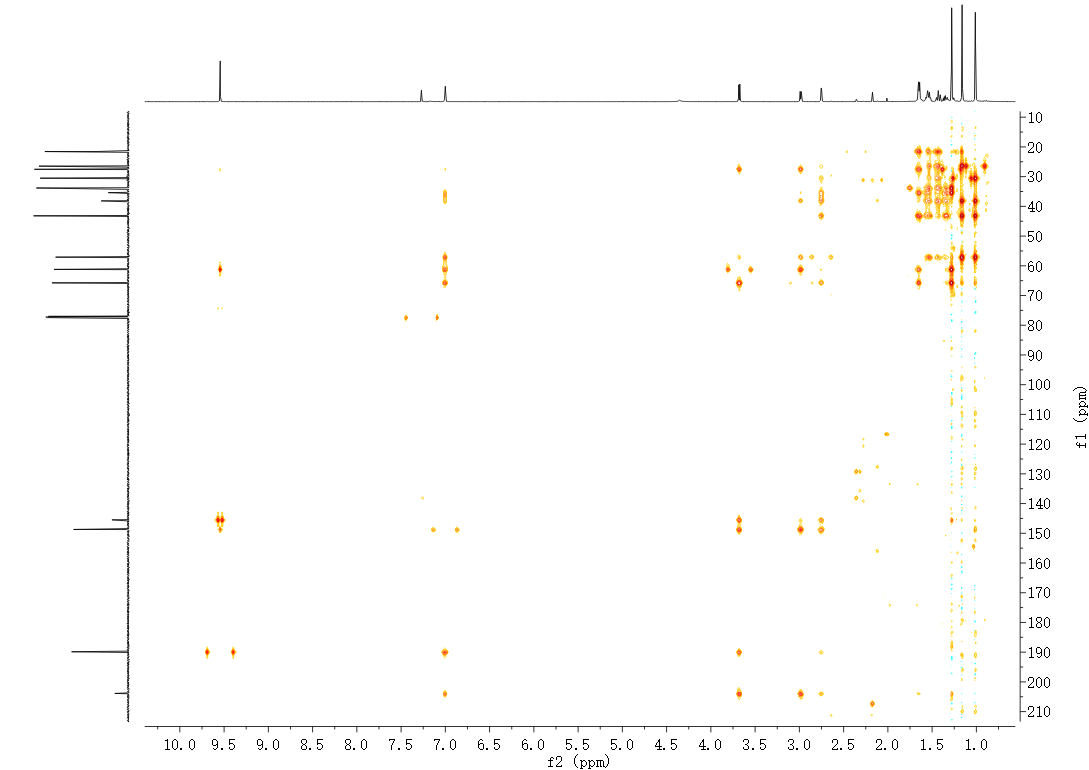


**Figure S5**. HMBC spectrum of artemyrianin A (**1**) recorded in CDCl_3_


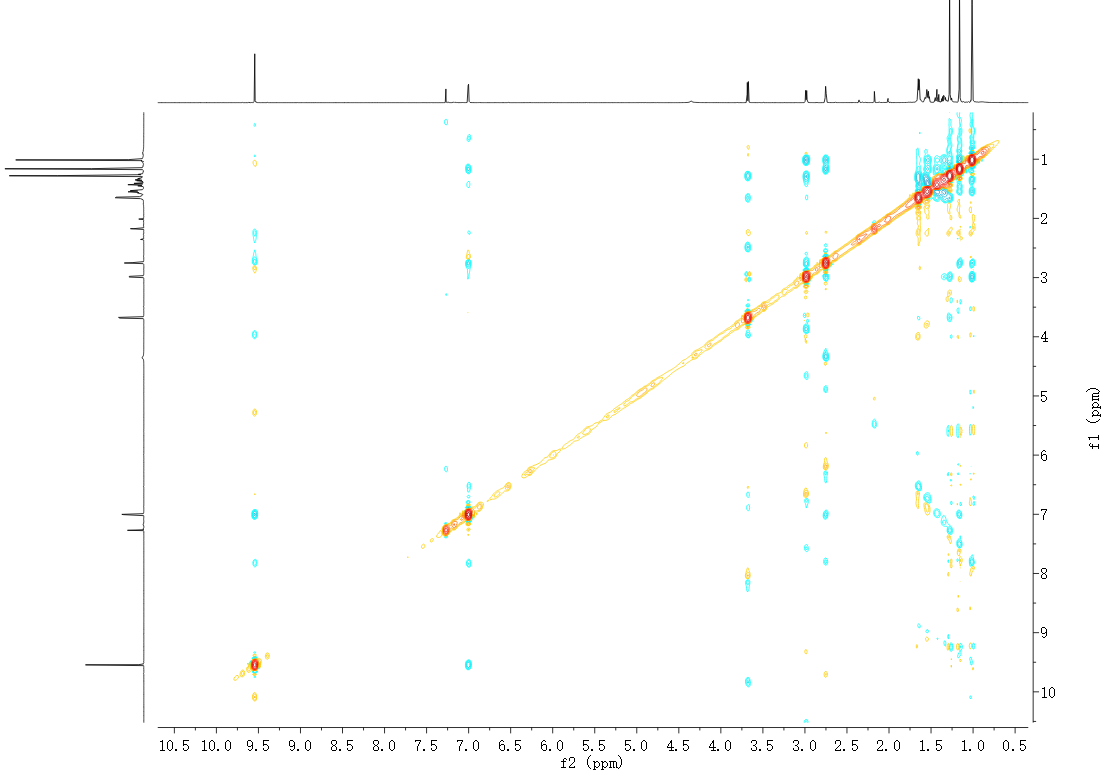


**Figure S6**. ROESY spectrum of artemyrianin A (**1**) recorded in CDCl_3_


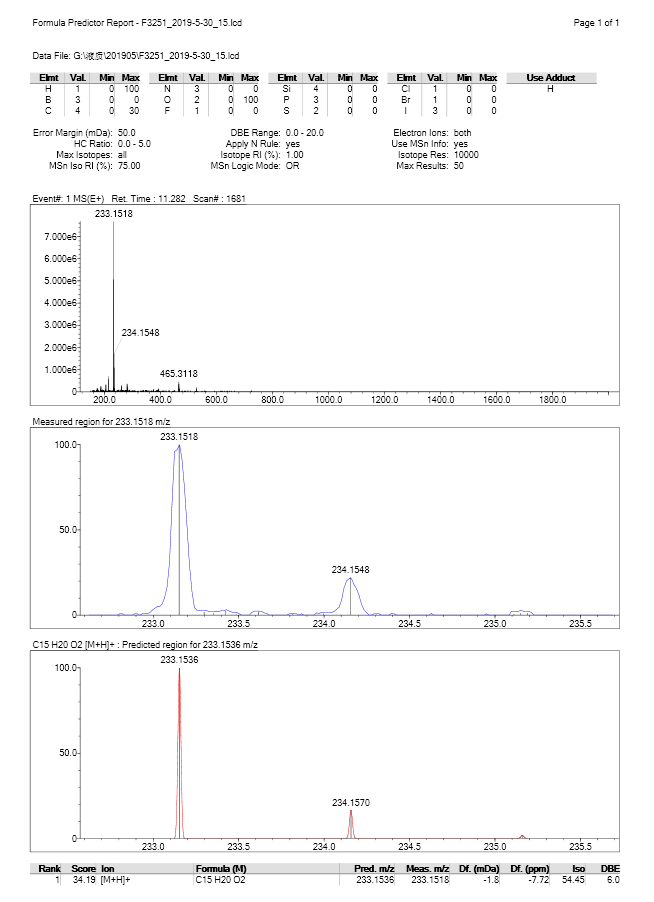


**Figure S7**. (+)-HRESIMS spectrum of artemyrianin A (**1**)


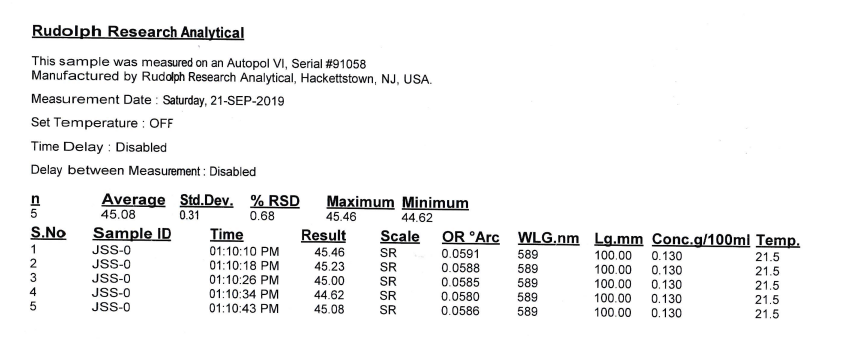


**Figure S8**. [*α*]_D_ spectrum of artemyrianin A (**1**) in MeOH


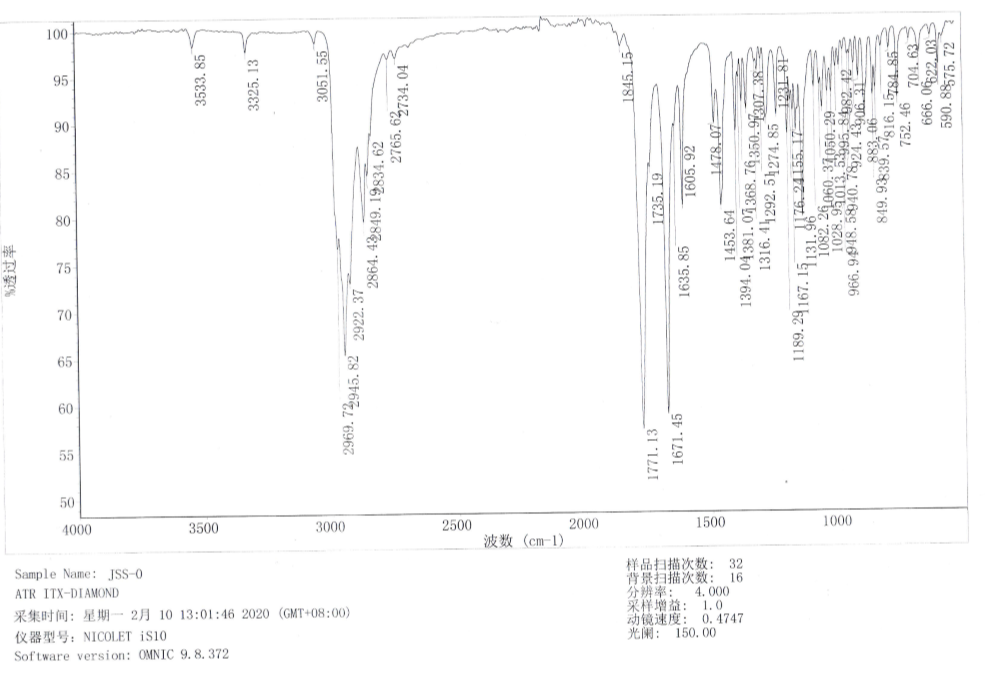


**Figure S9**. IR spectrum of artemyrianin A (**1**)


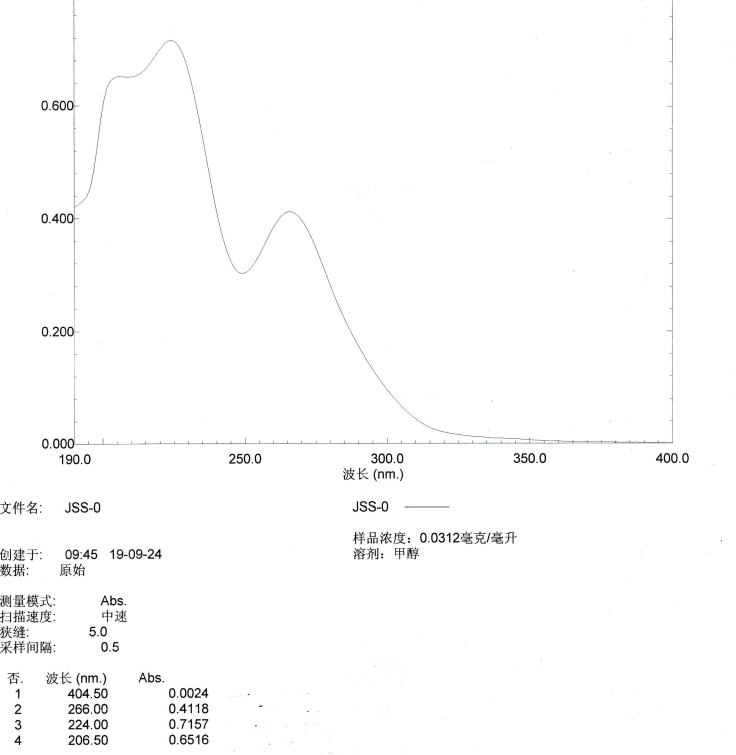


**Figure S10**. UV spectrum of artemyrianin A (**1**) in MeOH

 **
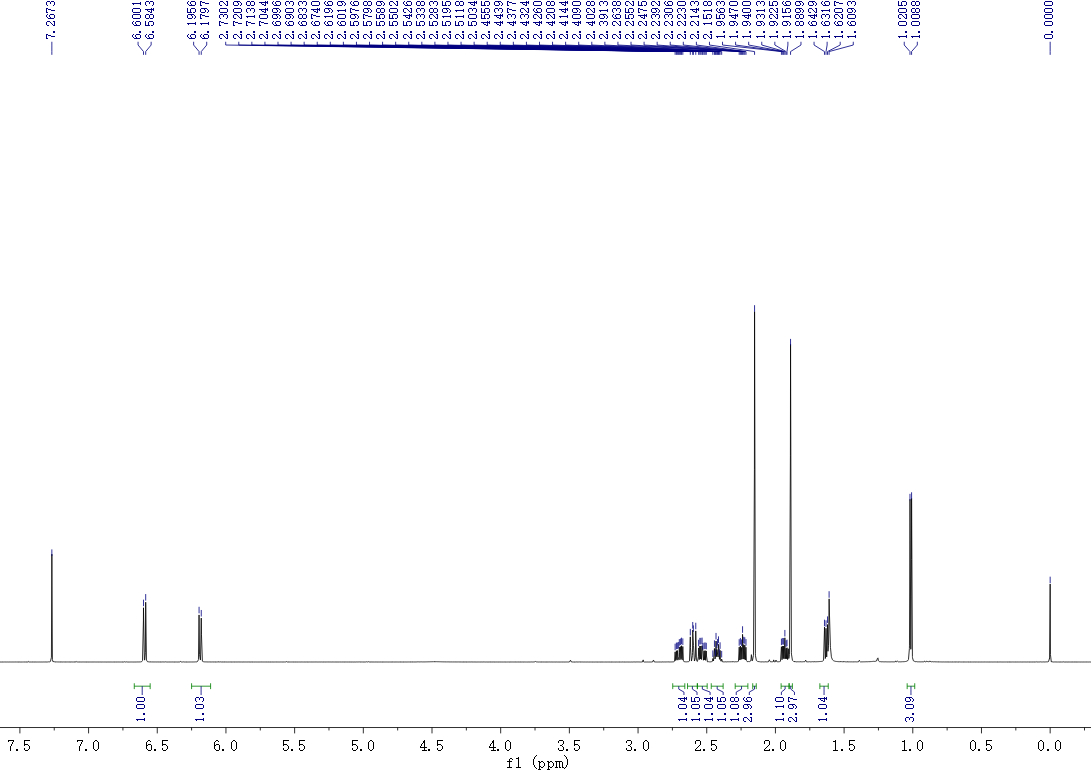
**

**Figure S11**. ^1^H NMR spectrum of artemyrianin B (**2**) recorded in CDCl_3_ at 600 MHz

**
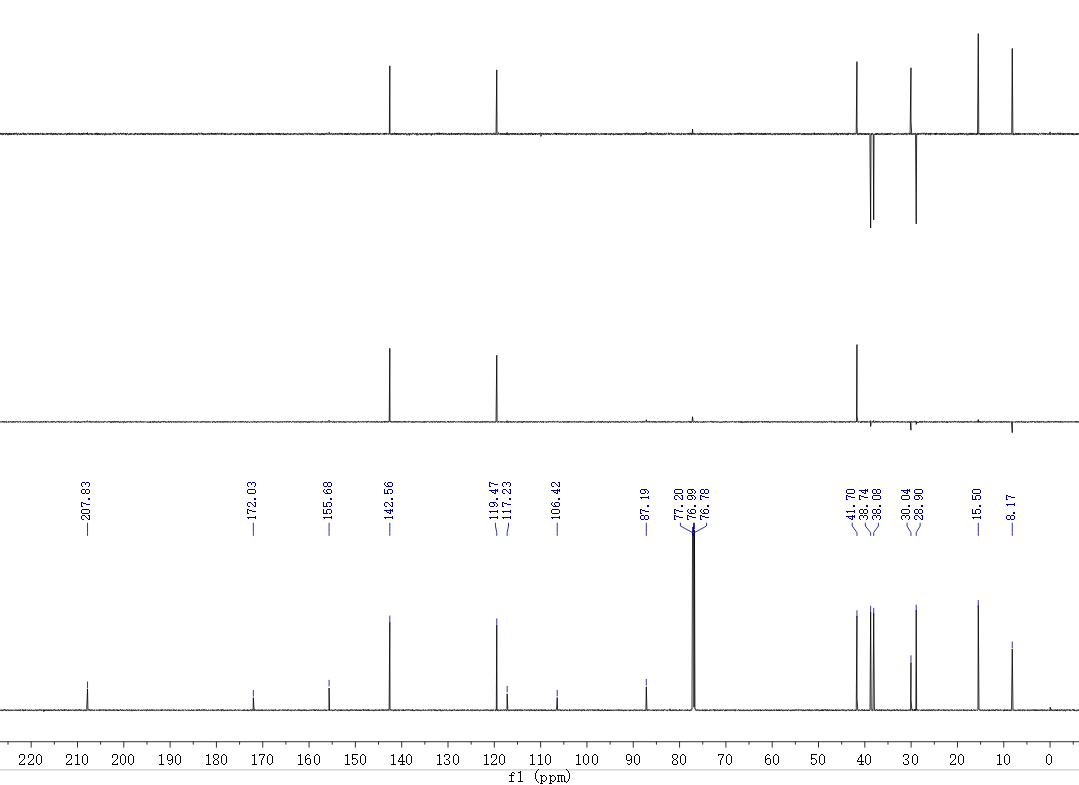
**

**Figure S12**. ^13^C NMR (DEPT) spectrum of artemyrianin B (**2**) recorded in CDCl_3_ at 150 MHz


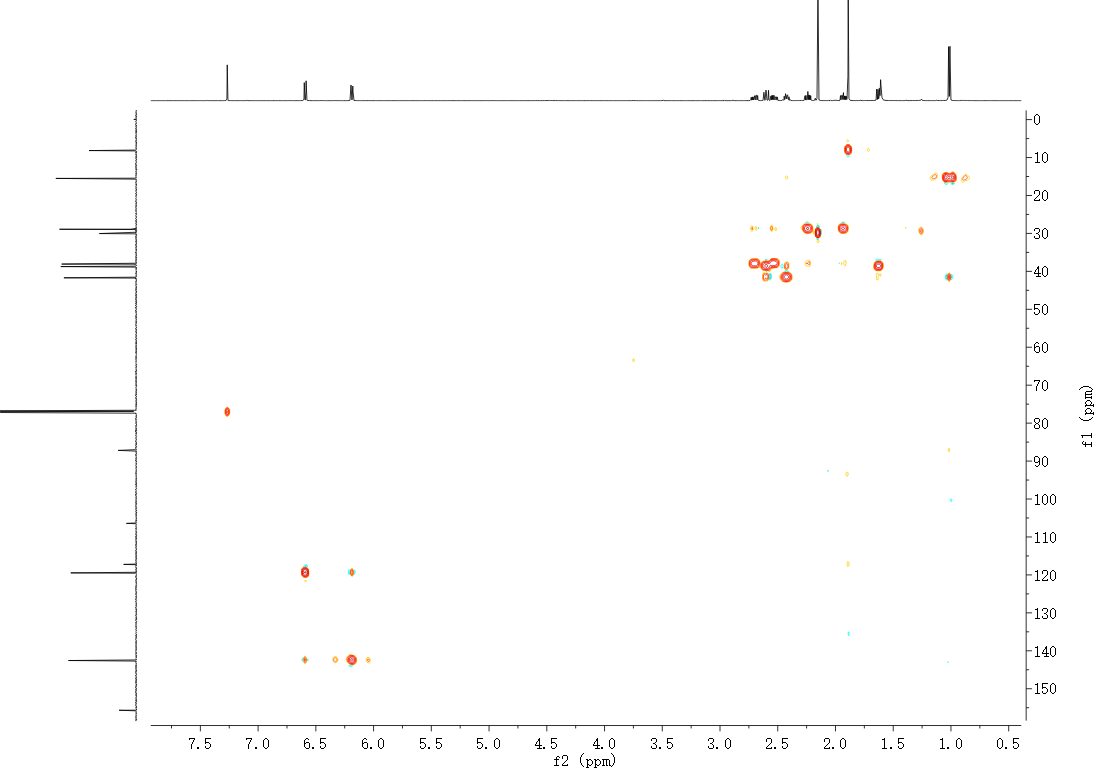


**Figure S13**. HSQC spectrum of artemyrianin B (**2**) recorded in CDCl_3_

**
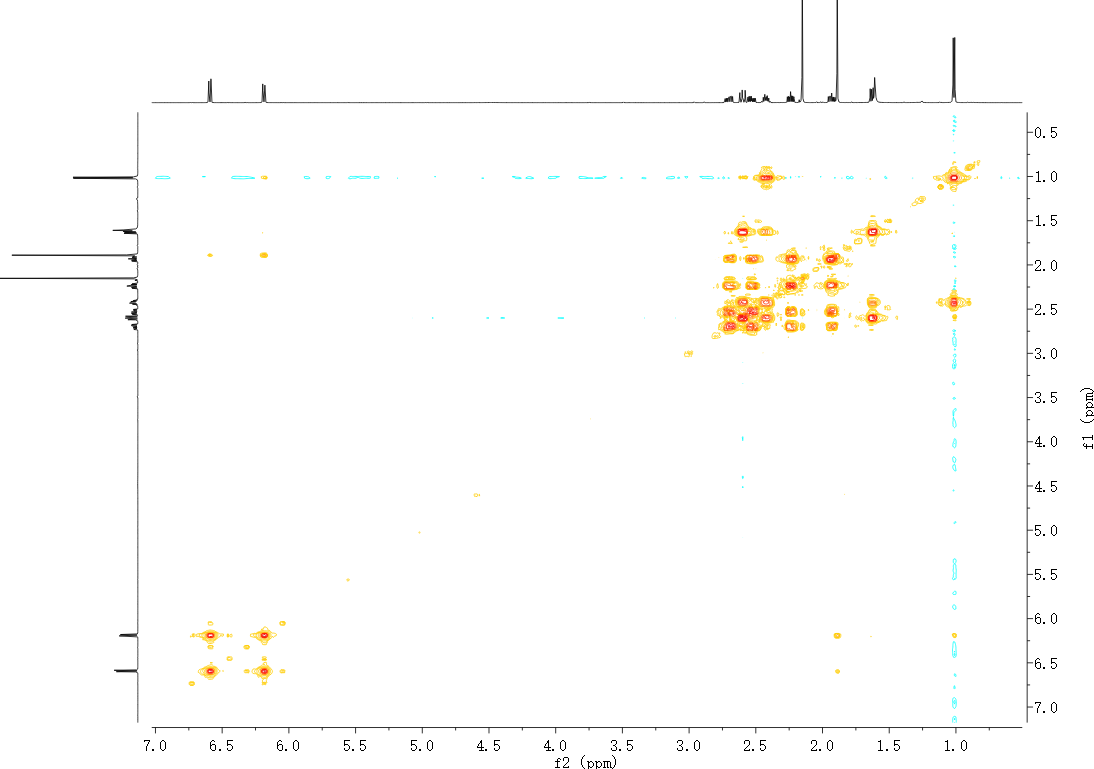
**

**Figure S14**. ^1^H−^1^H COSY spectrum of artemyrianin B (**2**) recorded in CDCl_3_


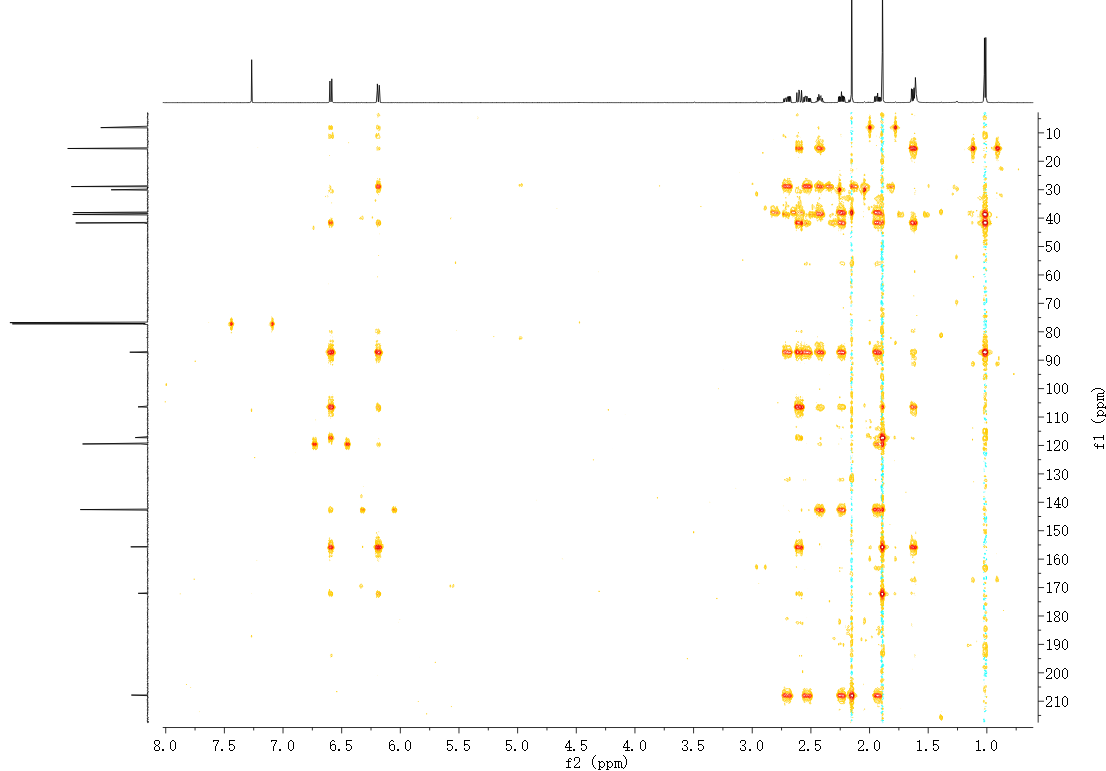


**Figure S15**. HMBC spectrum of artemyrianin B (**2**) recorded in CDCl_3_


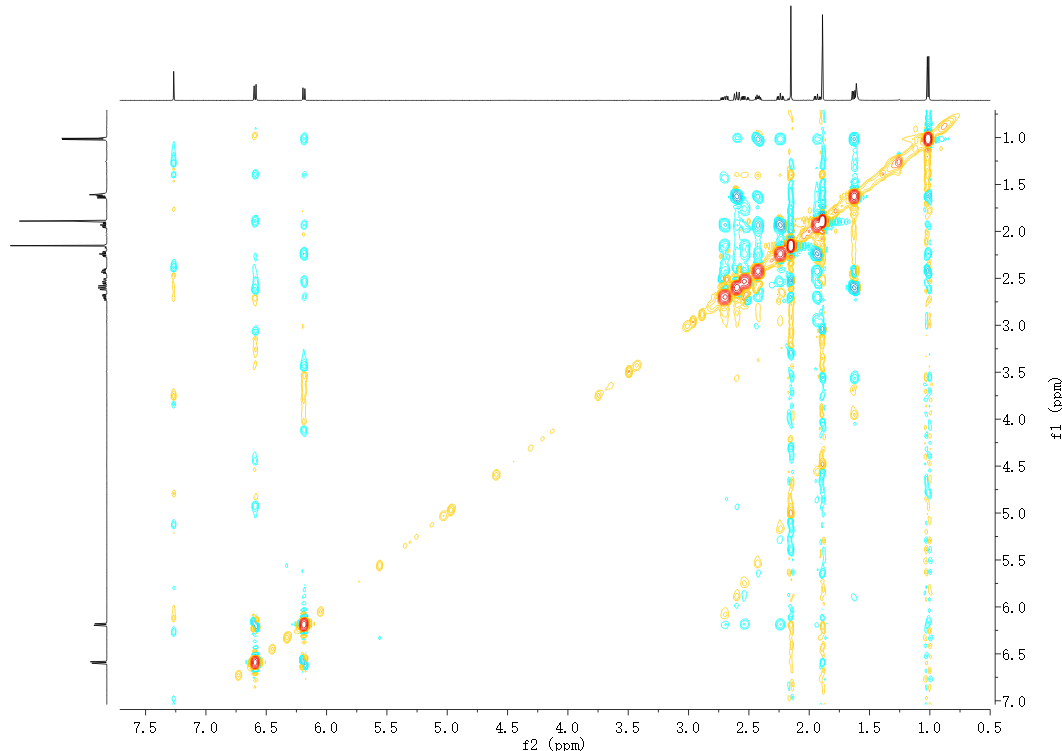


H_3_-13

H-6

**Figure S16**. ROESY spectrum of artemyrianin B (**2**) recorded in CDCl_3_


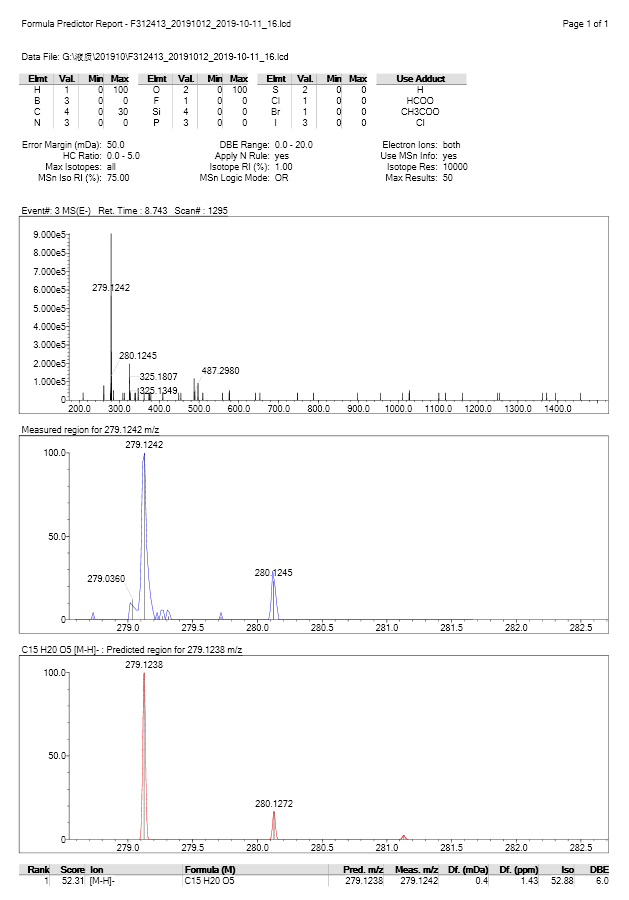


**Figure S17**. (−)-HRESIMS spectrum of artemyrianin B (**2**)


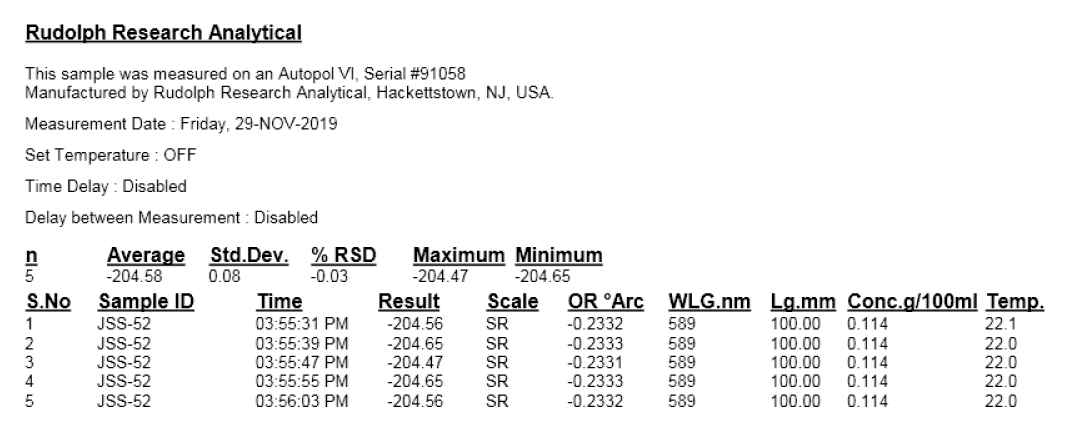


**Figure S18**. [*α*]_D_ spectrum of artemyrianin B (**2**) in MeOH


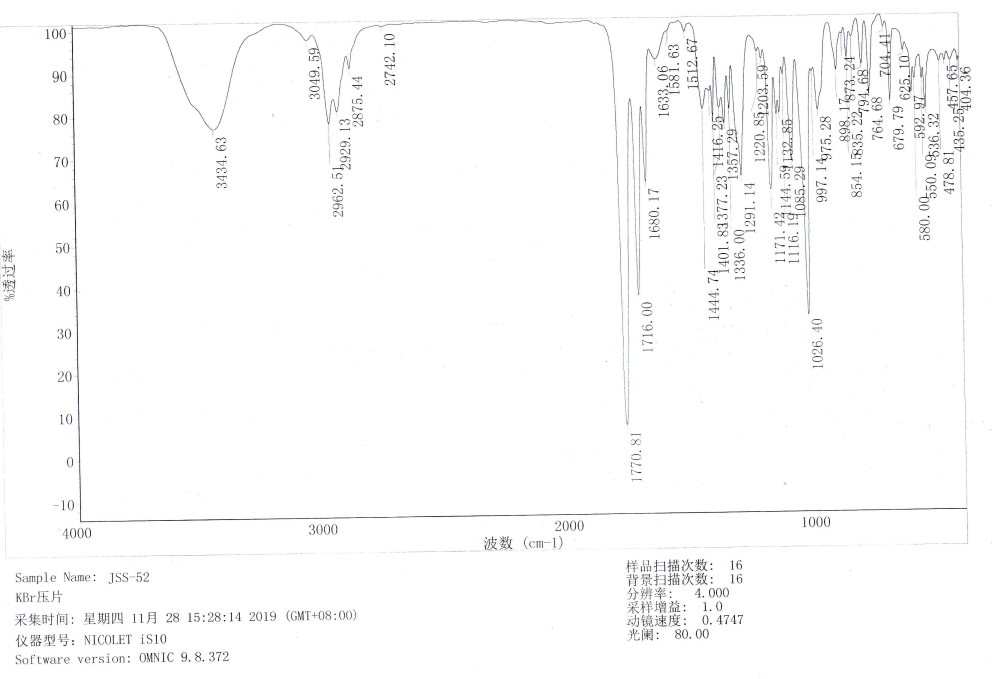


**Figure S19**. IR spectrum of artemyrianin B (**2**)


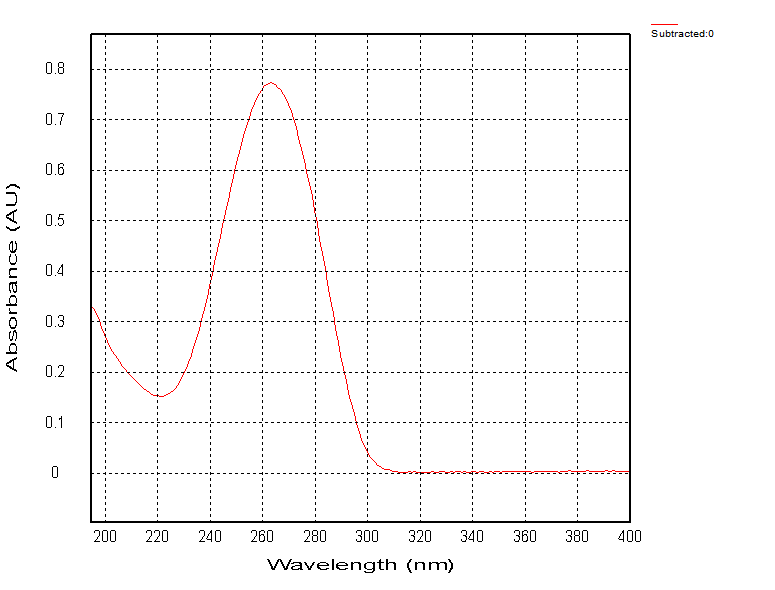


**Figure S20**. UV spectrum of artemyrianin B (**2**) in MeOH


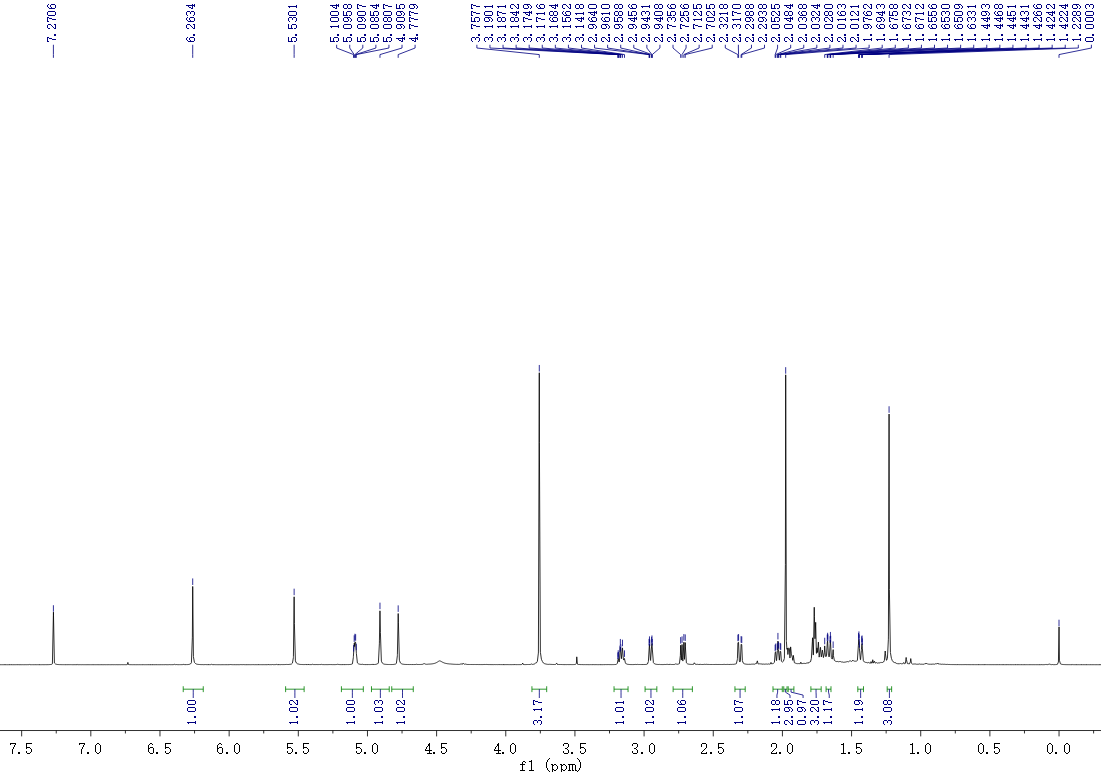


**Figure S21**. ^1^H NMR spectrum of artemyrianin C (**3**) recorded in CDCl_3_ at 600 MHz


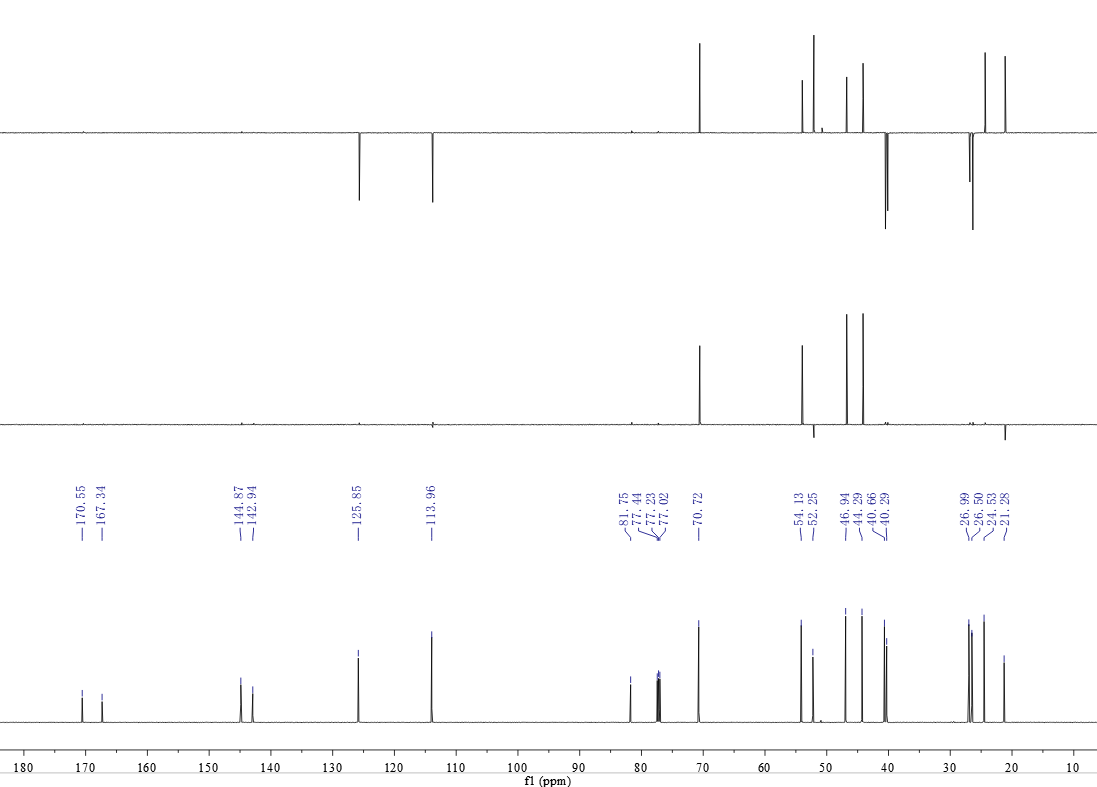


**Figure S22**. ^13^C NMR (DEPT) spectrum of artemyrianin C (**3**) recorded in CDCl_3_ at 150 MHz


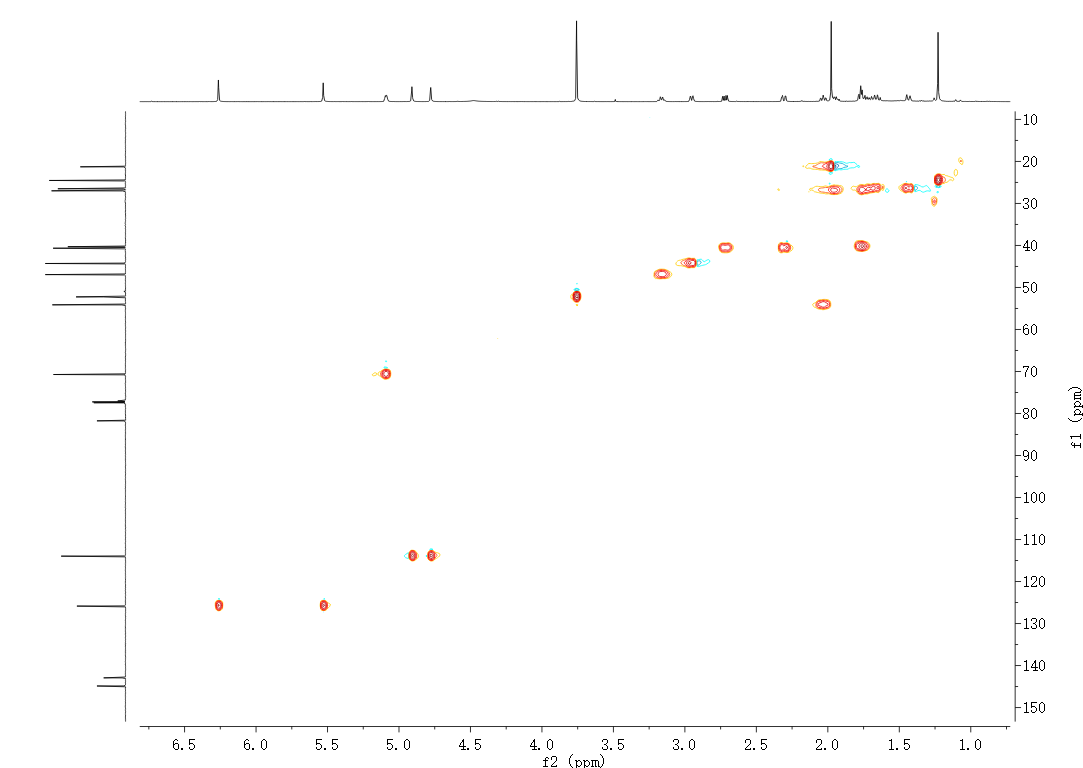


**Figure S23**. HSQC spectrum of artemyrianin C (**3**) recorded in CDCl_3_


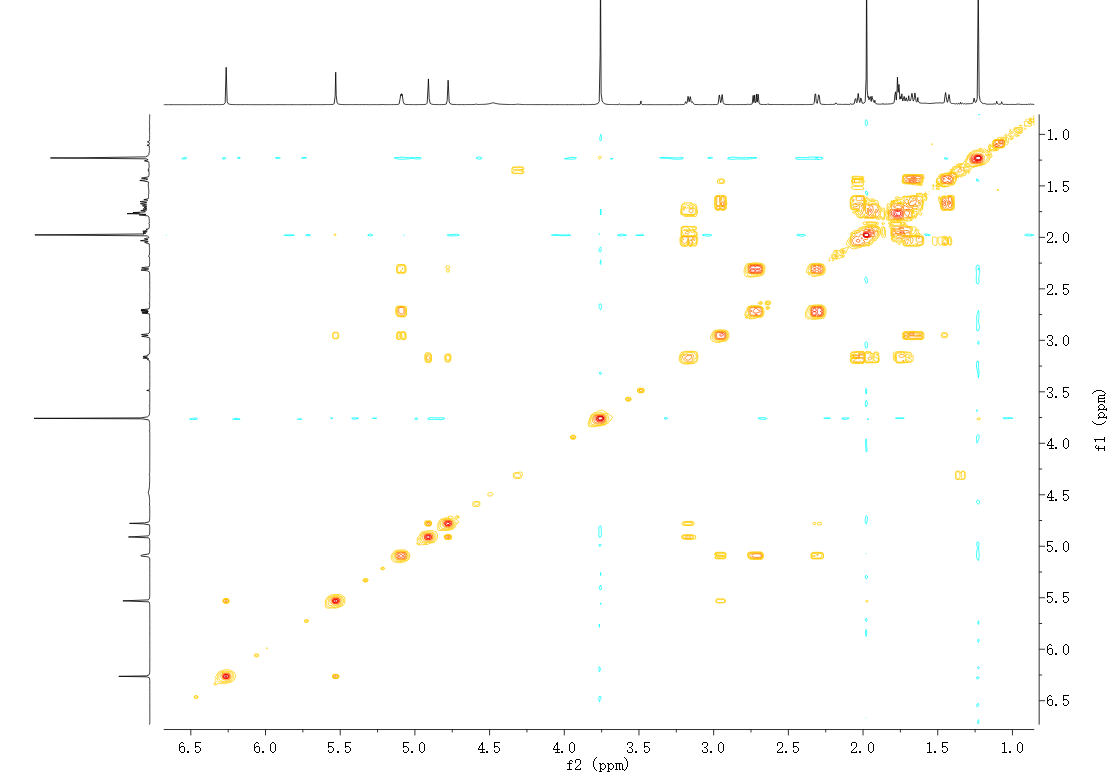


**Figure S24**. ^1^H−^1^H COSY spectrum of artemyrianin C (**3**) recorded in CDCl_3_


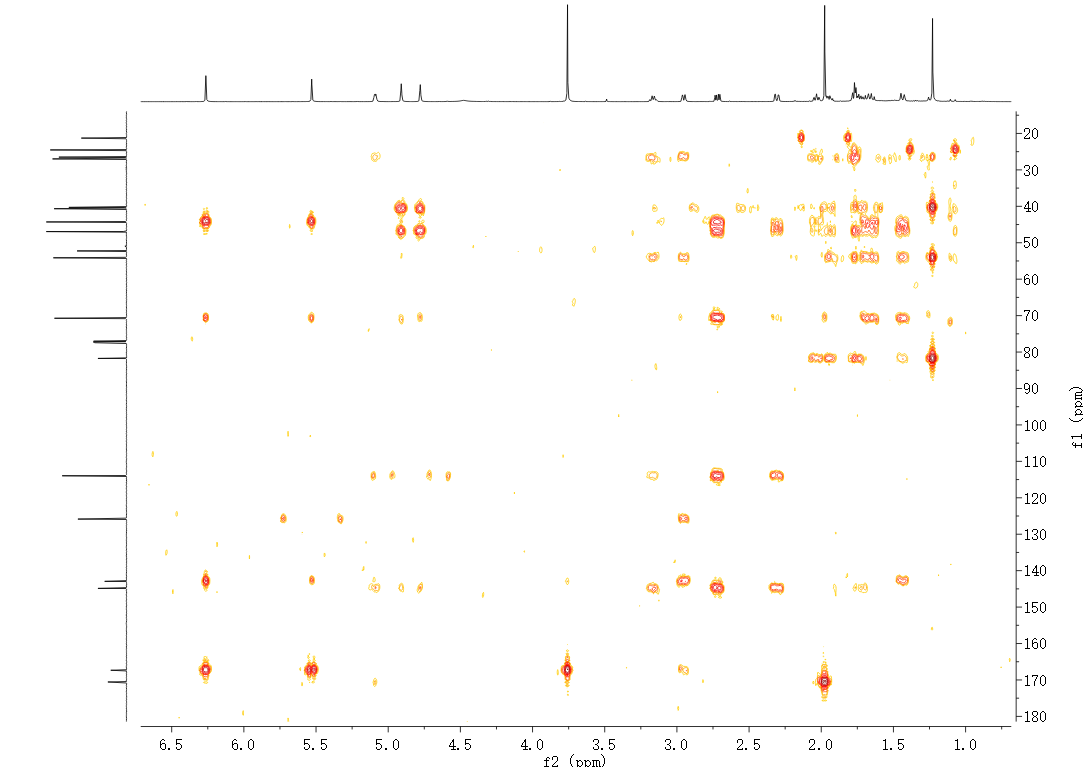


**Figure S25**. HMBC spectrum of artemyrianin C (**3**) recorded in CDCl_3_


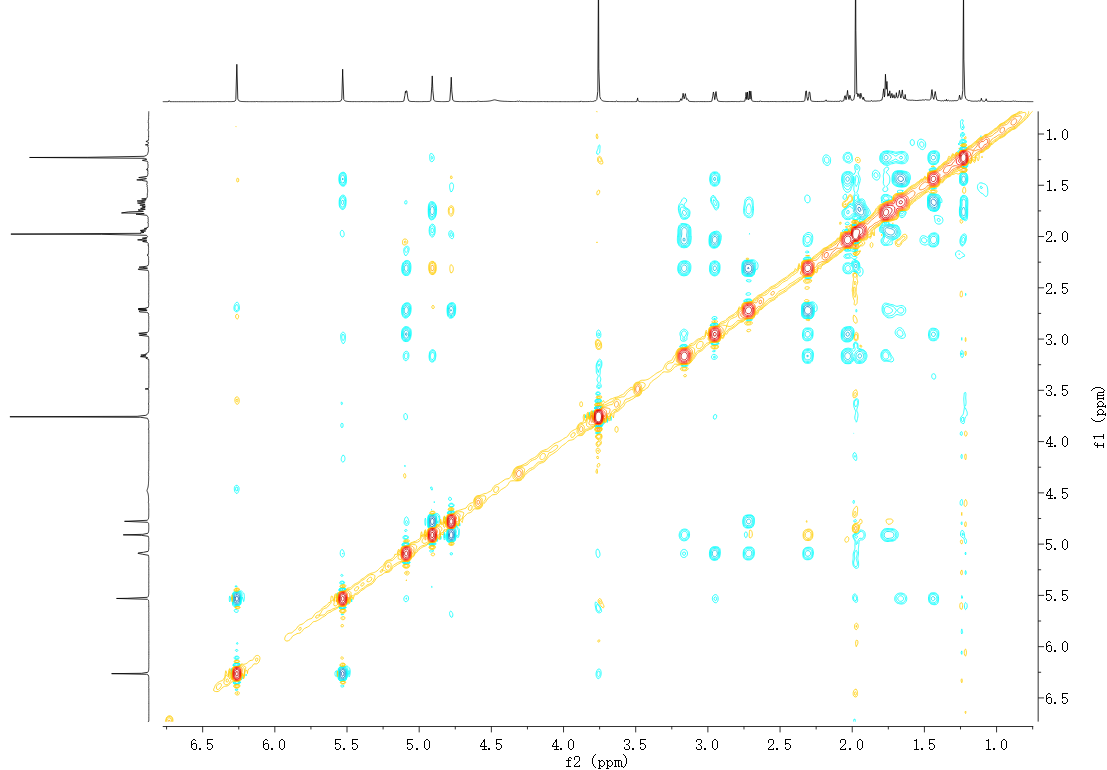


**Figure S26**. ROESY spectrum of artemyrianin C (**3**) recorded in CDCl_3_

_
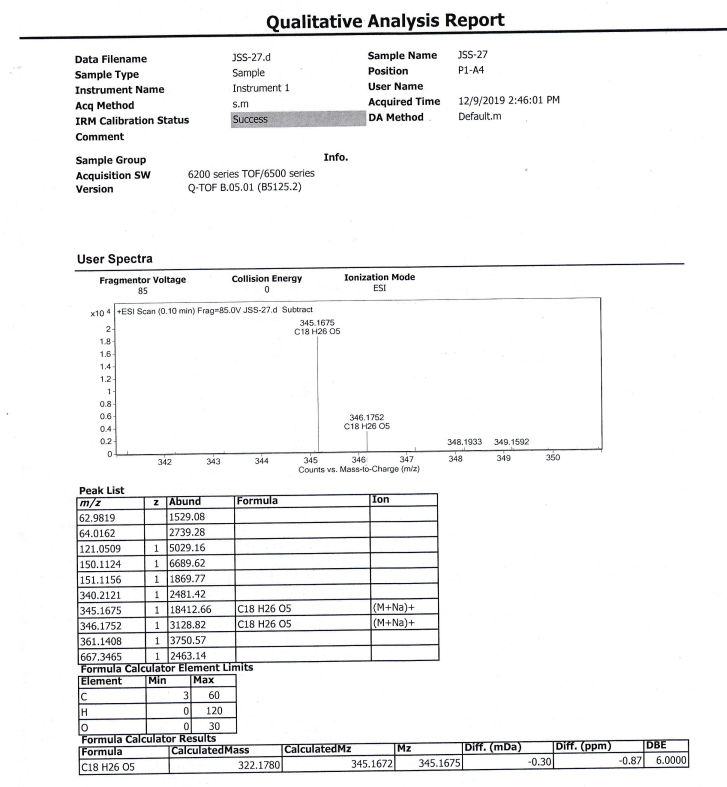
_

**Figure S27**. (+)-HRESIMS spectrum of artemyrianin C (**3**)


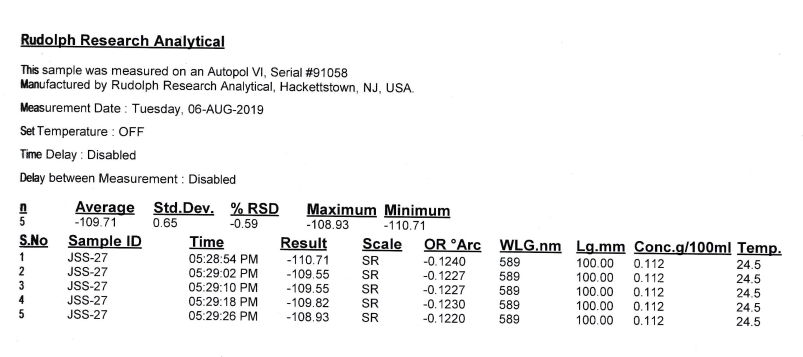


**Figure S28**. [*α*]_D_ spectrum of artemyrianin C (**3**) in MeOH


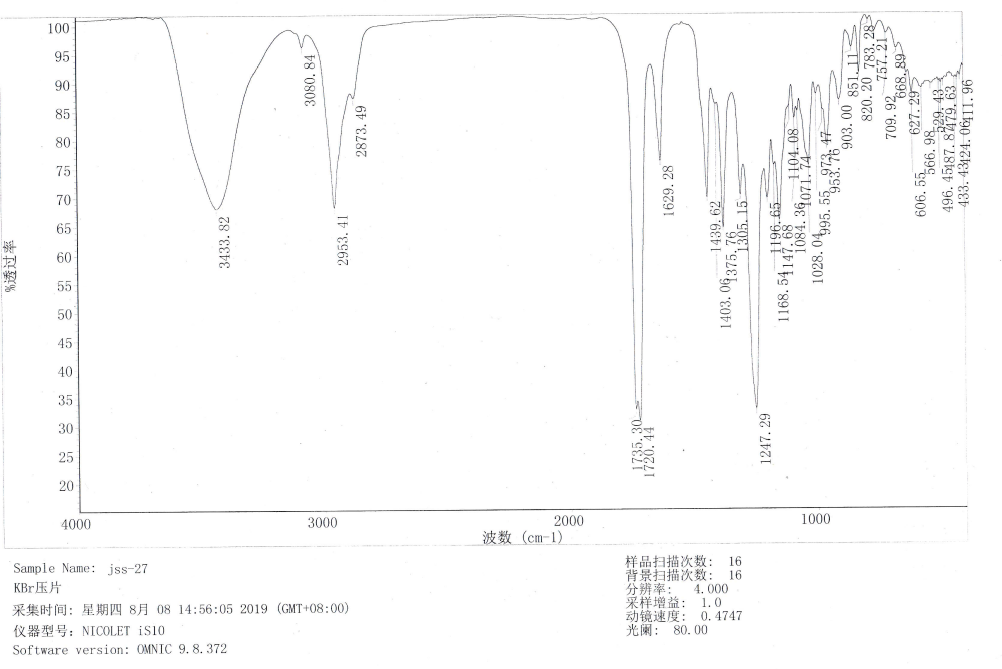


**Figure S29**. IR spectrum of artemyrianin C (**3**)


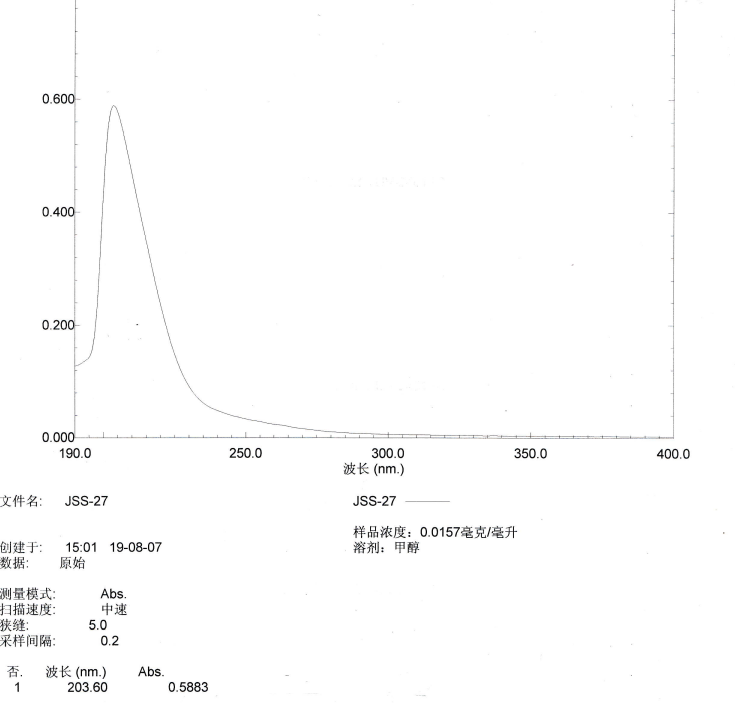


**Figure S30**. UV spectrum of artemyrianin C (**3**) in MeOH


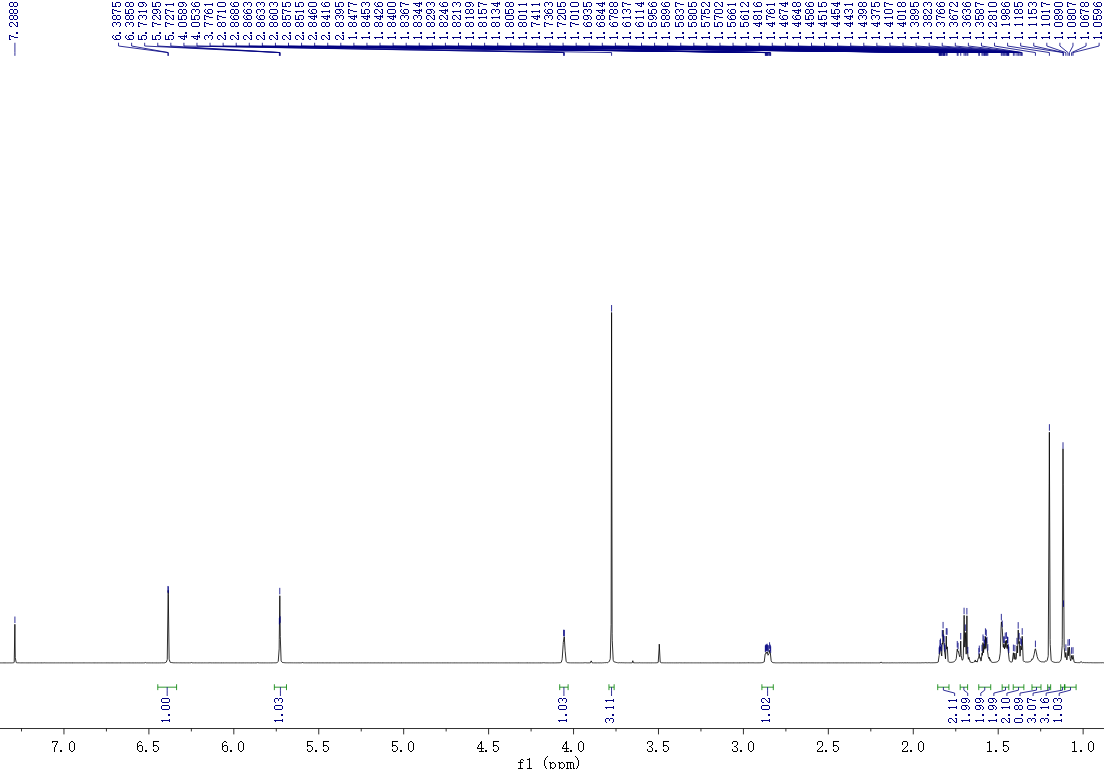


**Figure S31**. ^1^H NMR spectrum of artemyrianin D (**4**) recorded in CDCl_3_ at 600 MHz


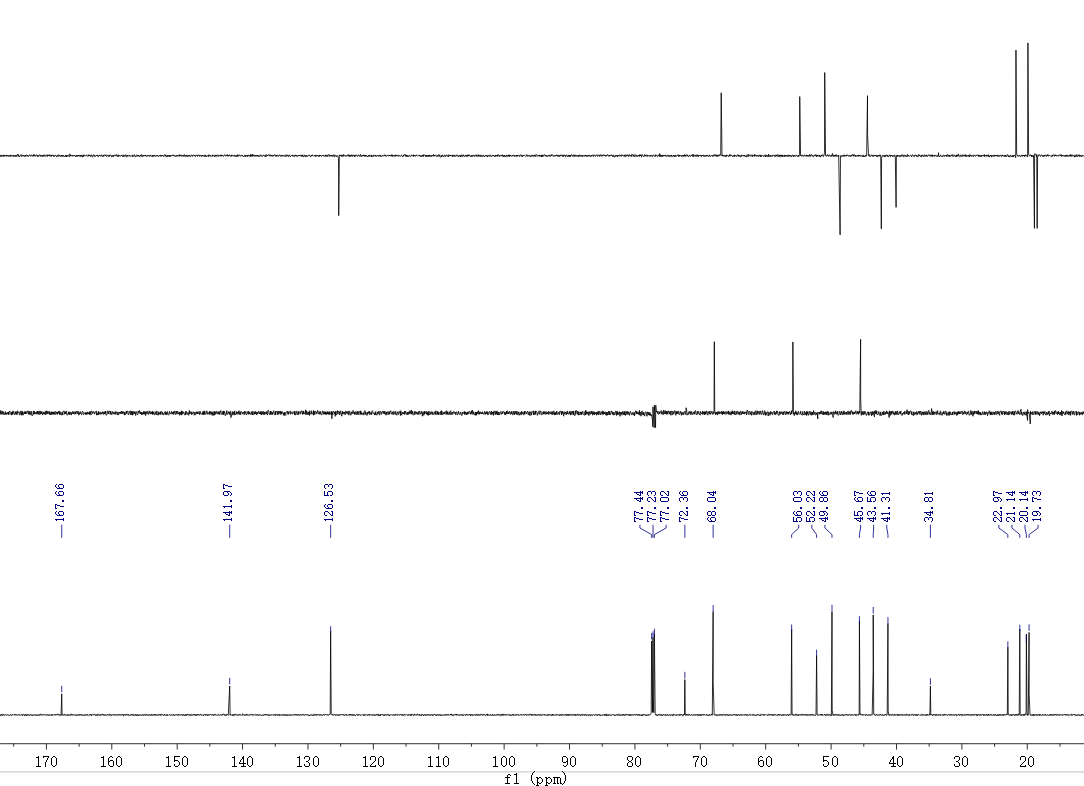


**Figure S32**. ^13^C NMR (DEPT) spectrum of artemyrianin D (**4**) recorded in CDCl_3_ at 150 MHz


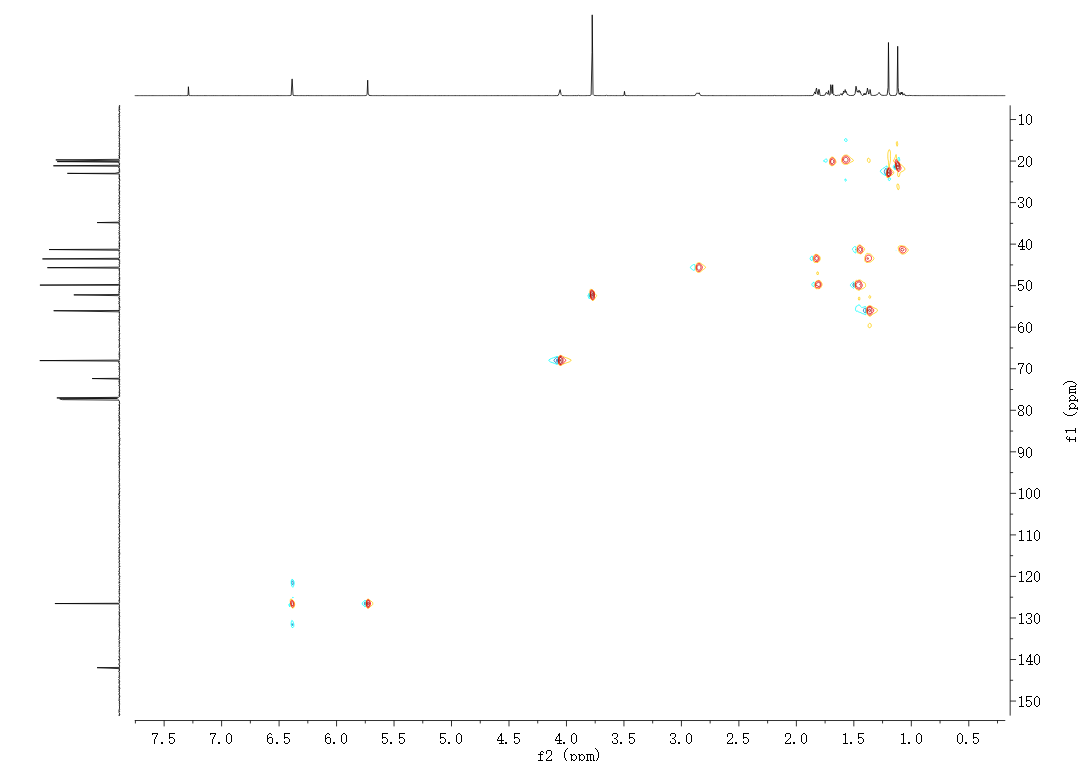


**Figure S33**. HSQC spectrum of artemyrianin D (**4**) recorded in CDCl_3_


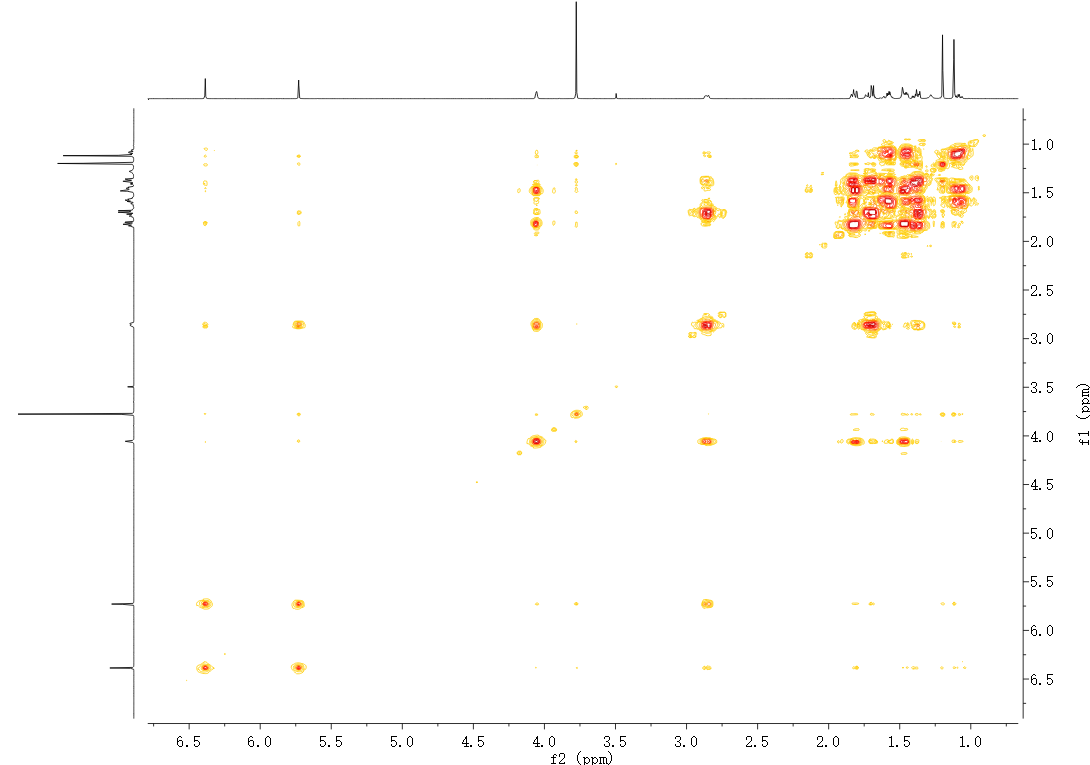


**Figure S34**. ^1^H−^1^H COSY spectrum of artemyrianin D (**4**) recorded in CDCl_3_


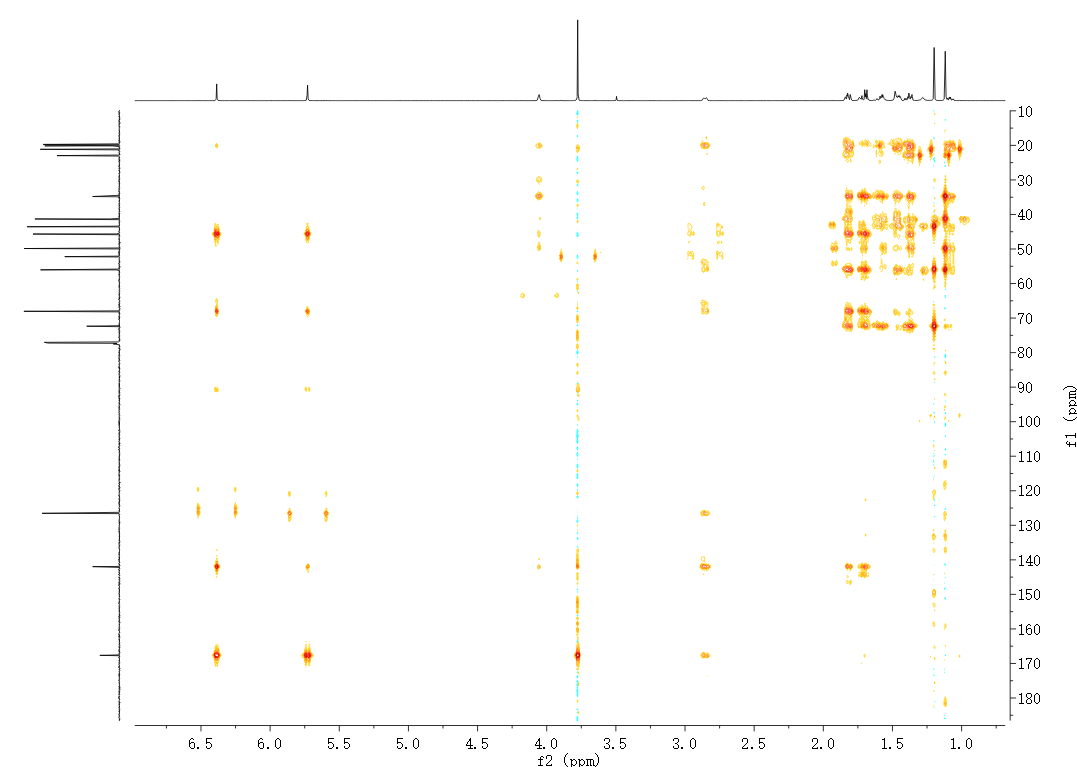


**Figure S35**. HMBC spectrum of artemyrianin D (**4**) recorded in CDCl_3_


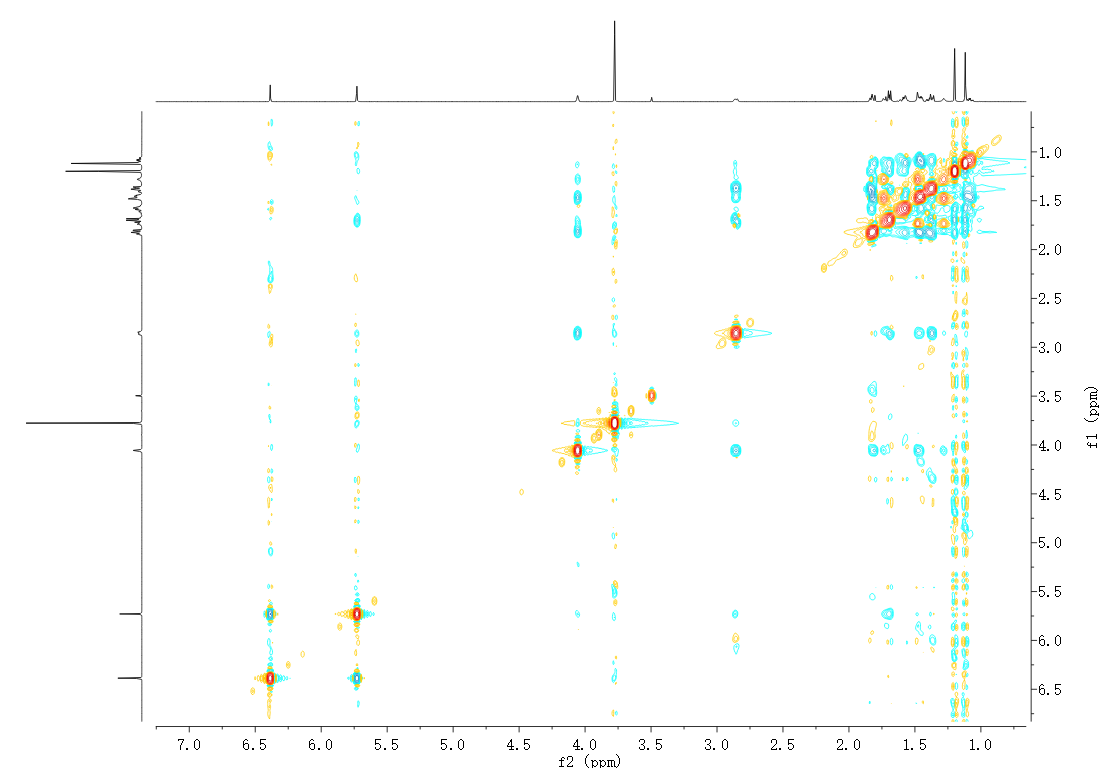


**Figure S36**. ROESY spectrum of artemyrianin D (**4**) recorded in CDCl_3_

_
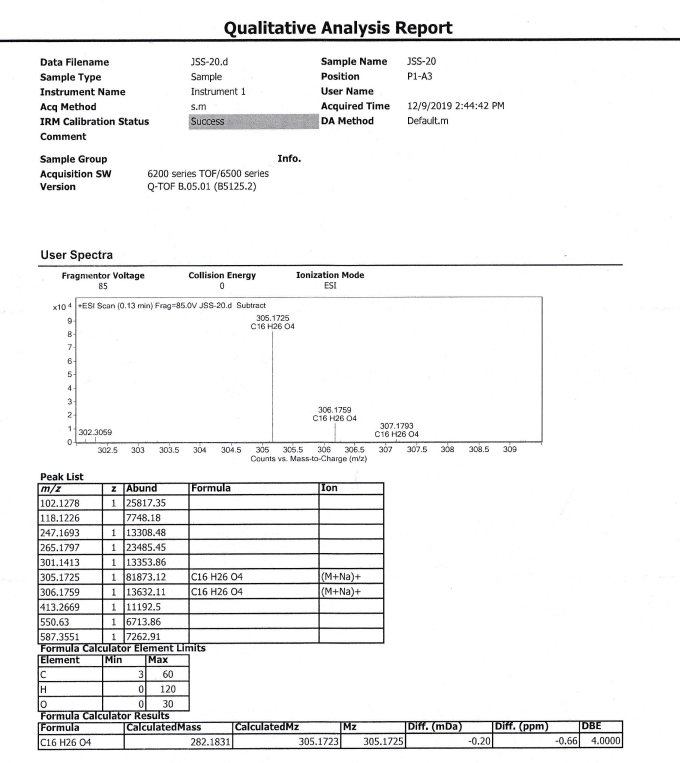
_

**Figure S37**. (+)-HRESIMS spectrum of artemyrianin D (**4**)


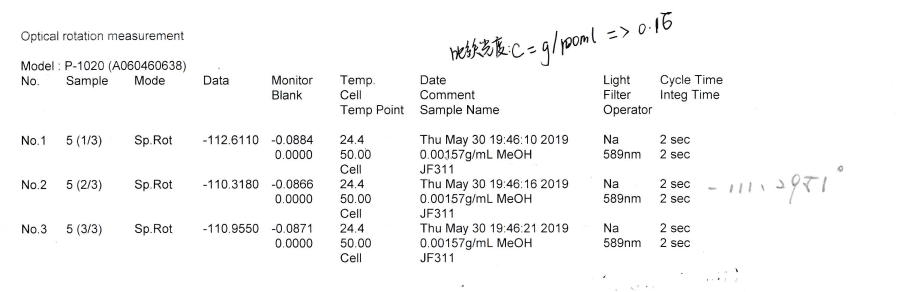


**Figure S38**. [*α*]_D_ spectrum of artemyrianin D (**4**) in MeOH


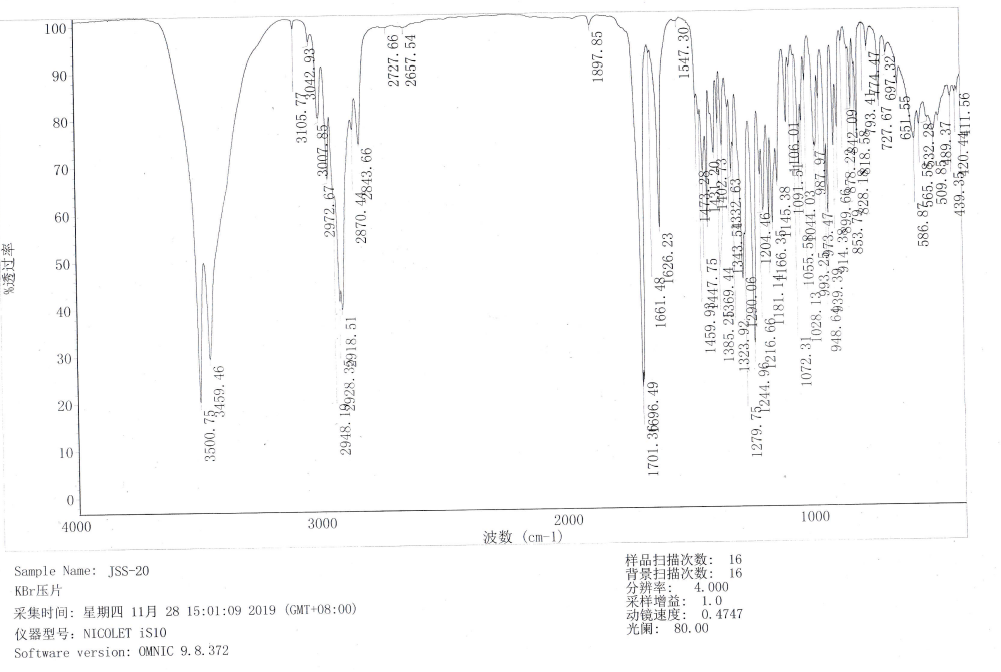


**Figure S39**. IR spectrum of artemyrianin D (**4**)


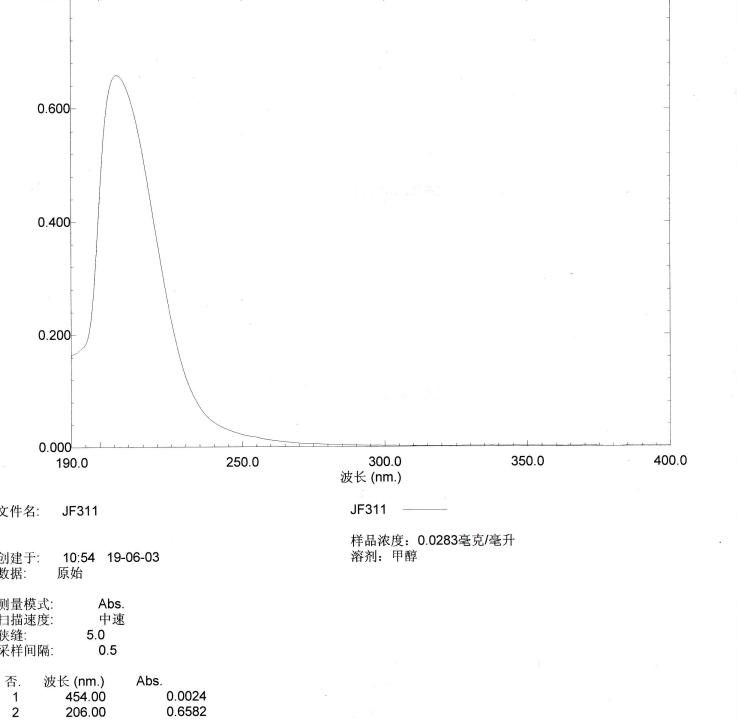


**Figure S40**. UV spectrum of artemyrianin D (**4**) in MeOH

**
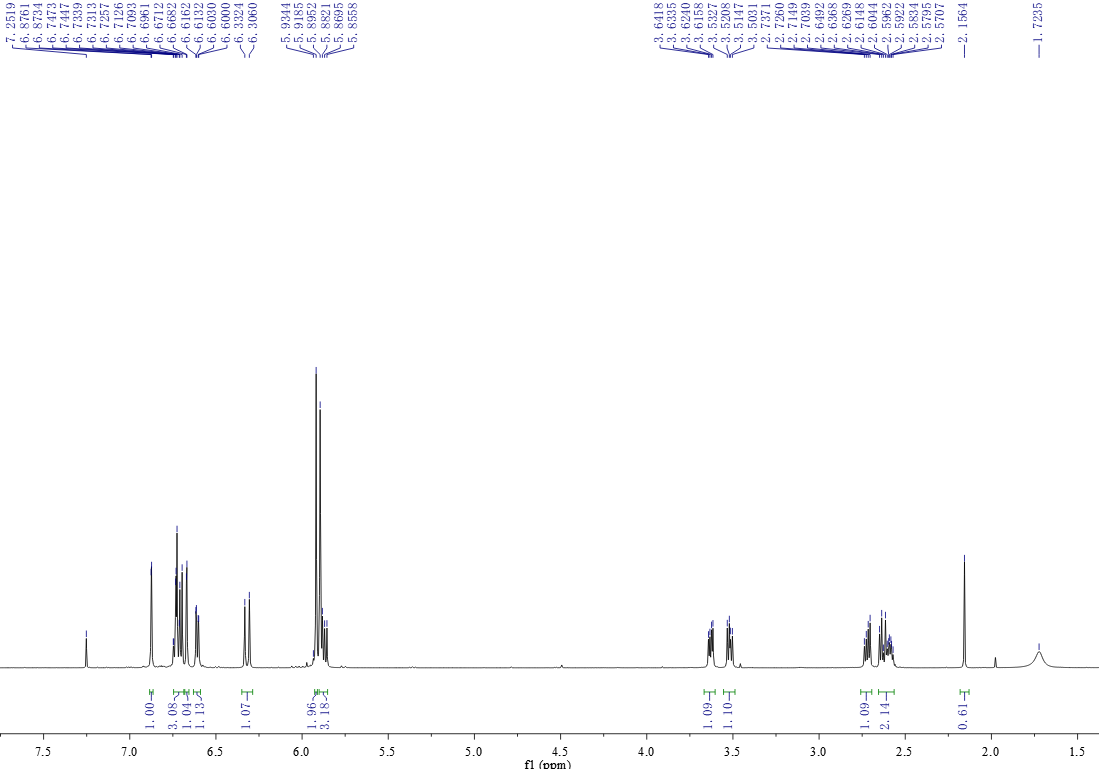
**

**Figure S41**. ^1^H NMR spectrum of artemyrianin E (**5**) recorded in CDCl_3_ at 600 MHz

**
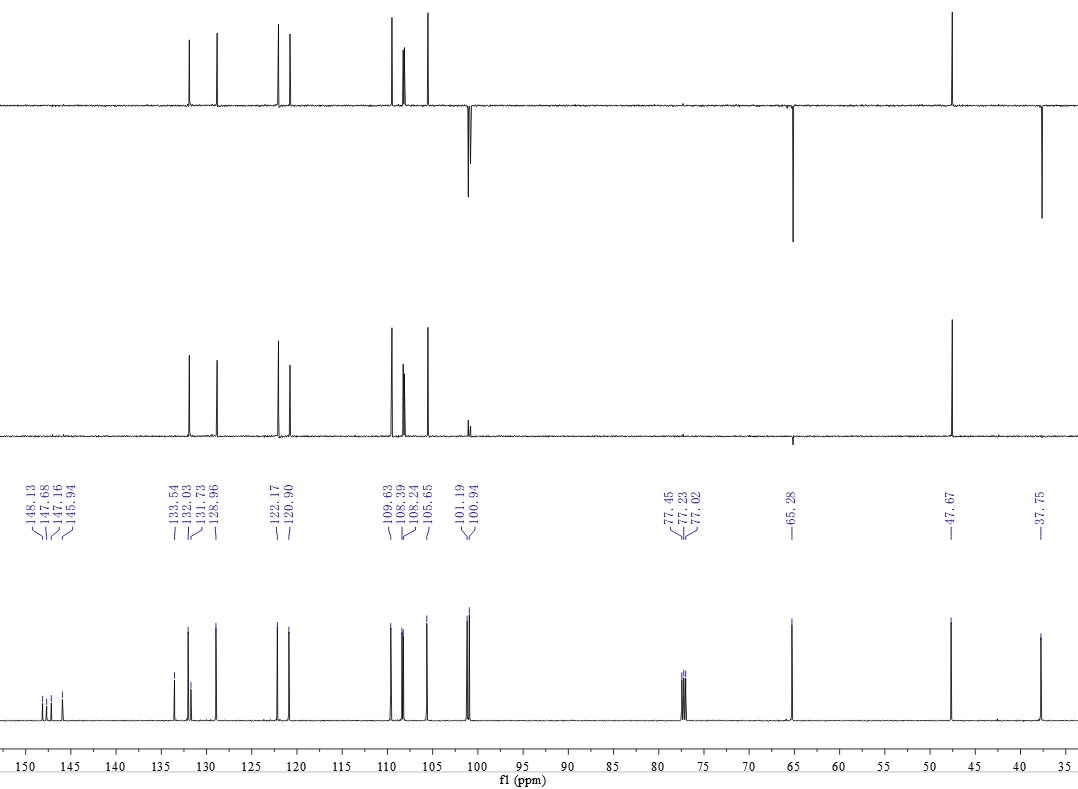
**

**Figure S42**. ^13^C NMR (DEPT) spectrum of artemyrianin E (**5**) recorded in CDCl_3_ at 150 MHz

**
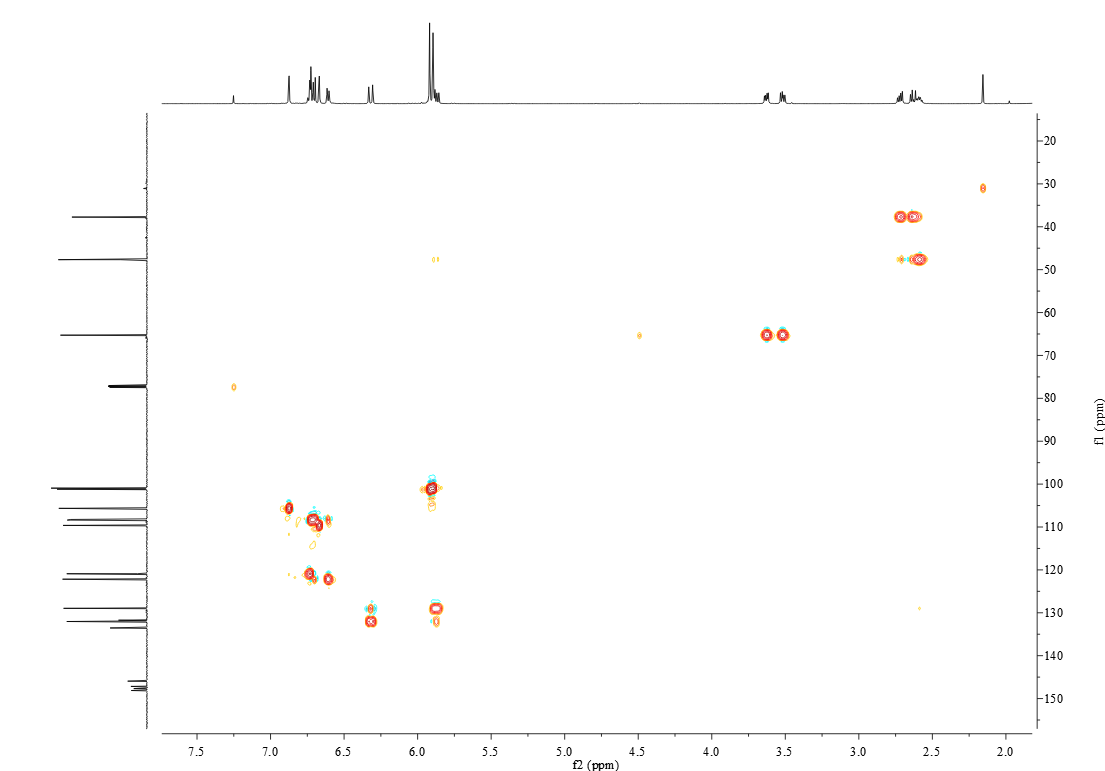
**

**Figure S43**. HSQC spectrum of artemyrianin E (**5**) recorded in CDCl_3_

**
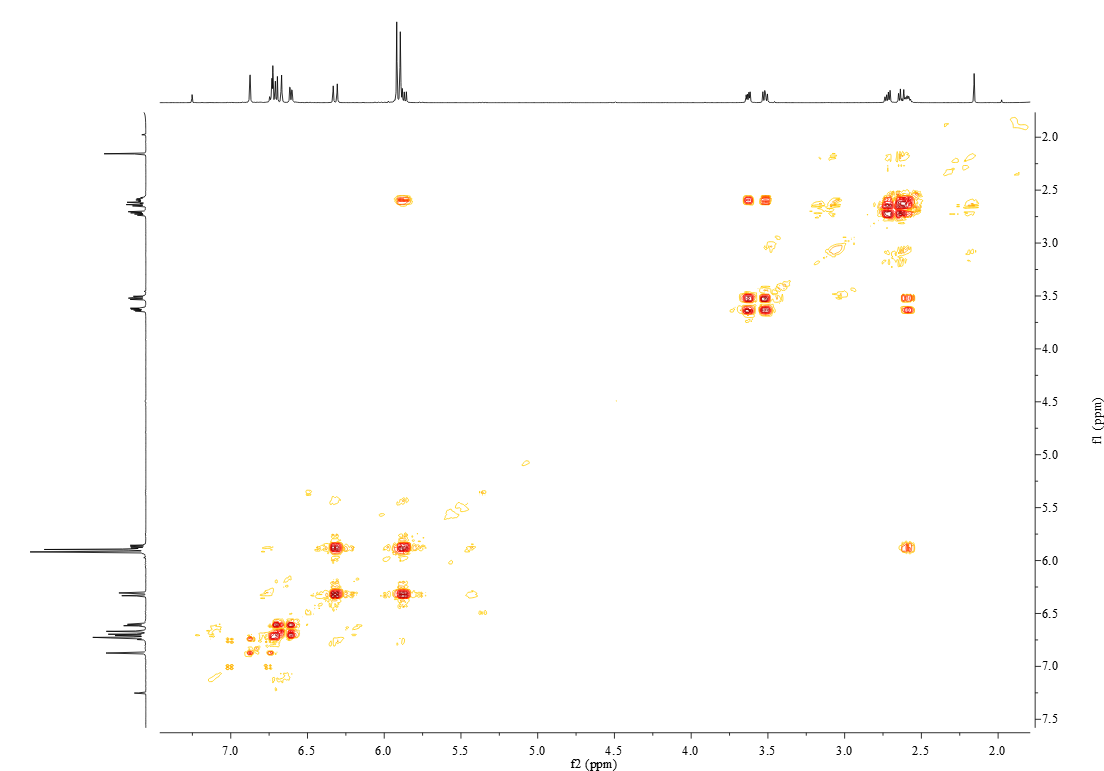
**

**Figure S44**. ^1^H−^1^H COSY spectrum of artemyrianin E (**5**) recorded in CDCl_3_

**
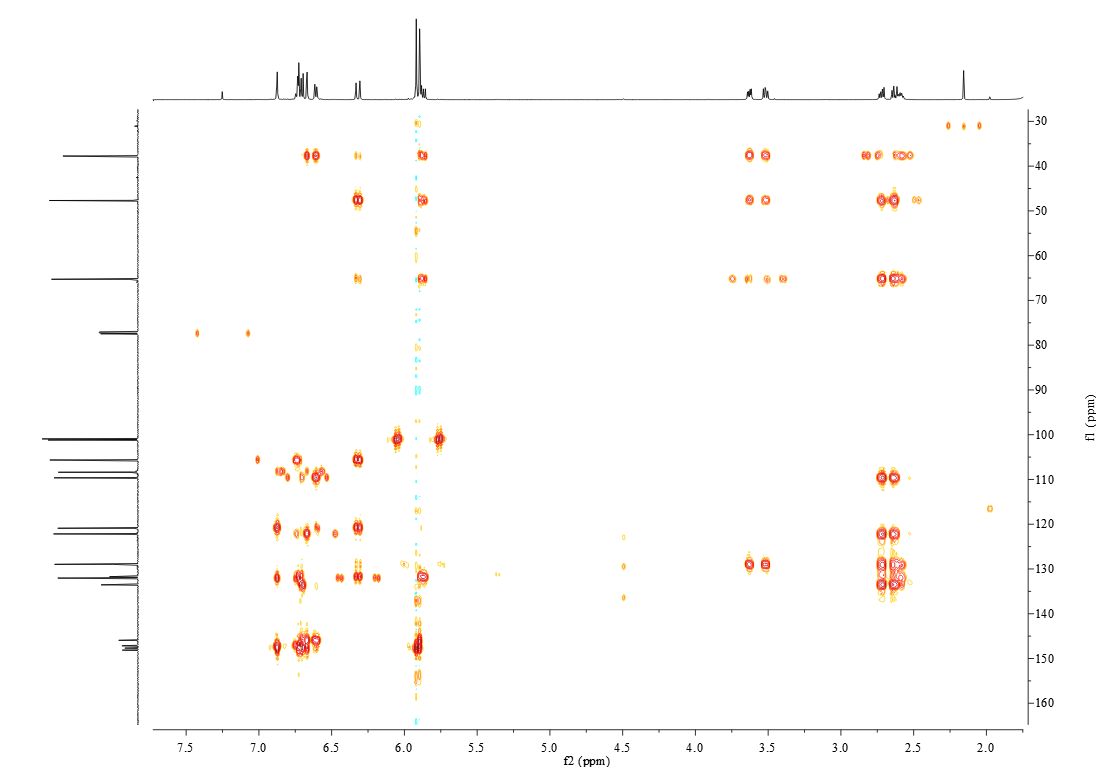
**

**Figure S45**. HMBC spectrum of artemyrianin E (**5**) recorded in CDCl_3_

**
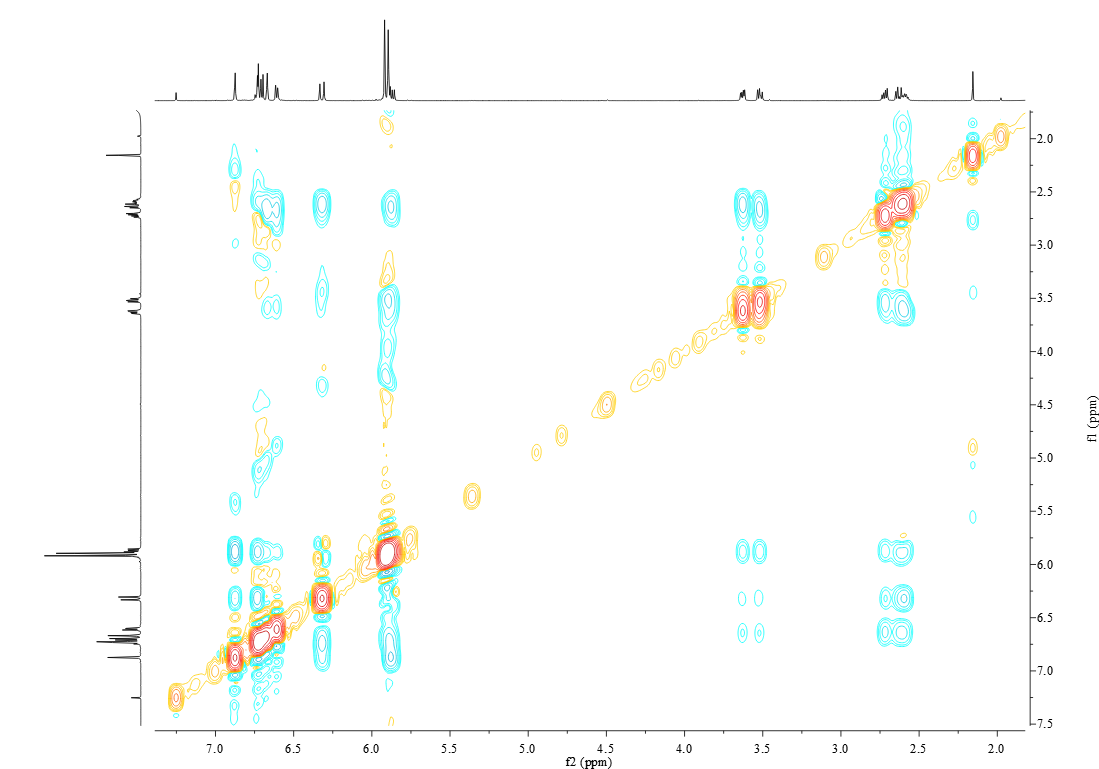
**

**Figure S46**. ROESY spectrum of artemyrianin E (**5**) recorded in CDCl_3_

**
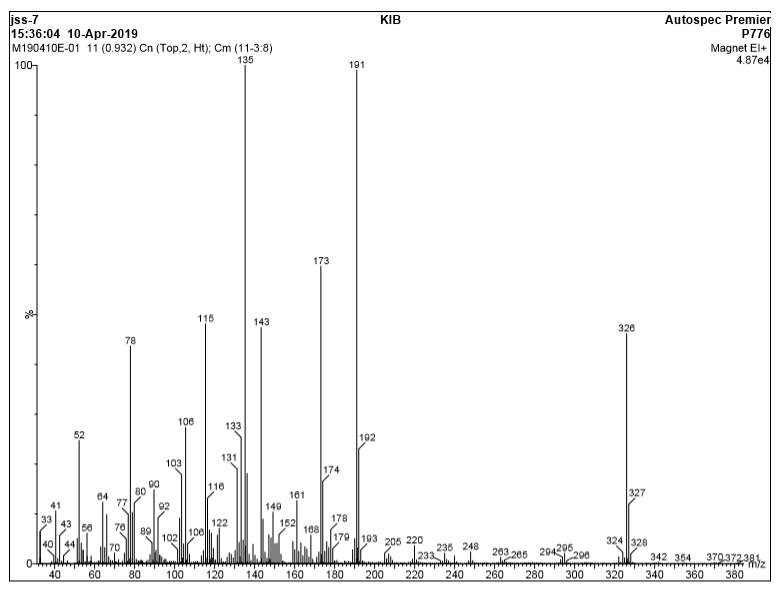
**

**Figure S47**. EIMS spectrum of artemyrianin E (**5**)

**
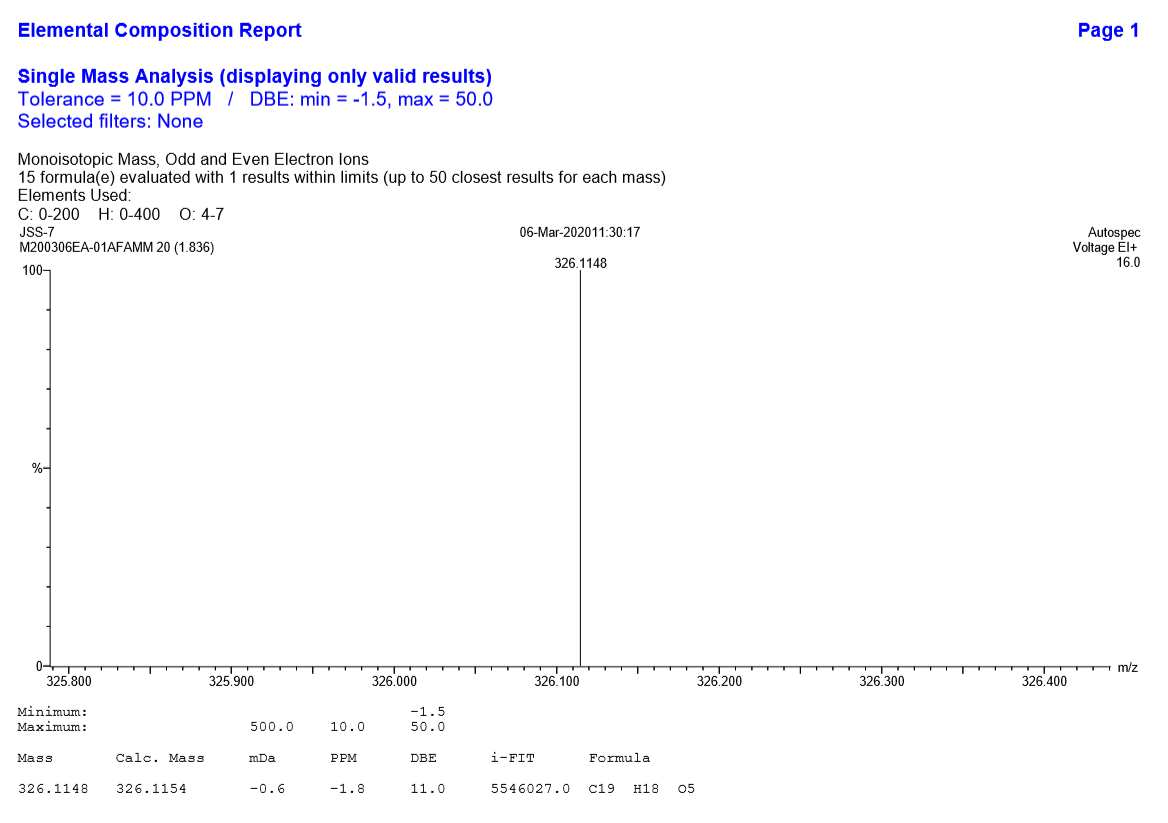
**

**Figure S48**. HREIMS spectrum of artemyrianin E (**5**)

**
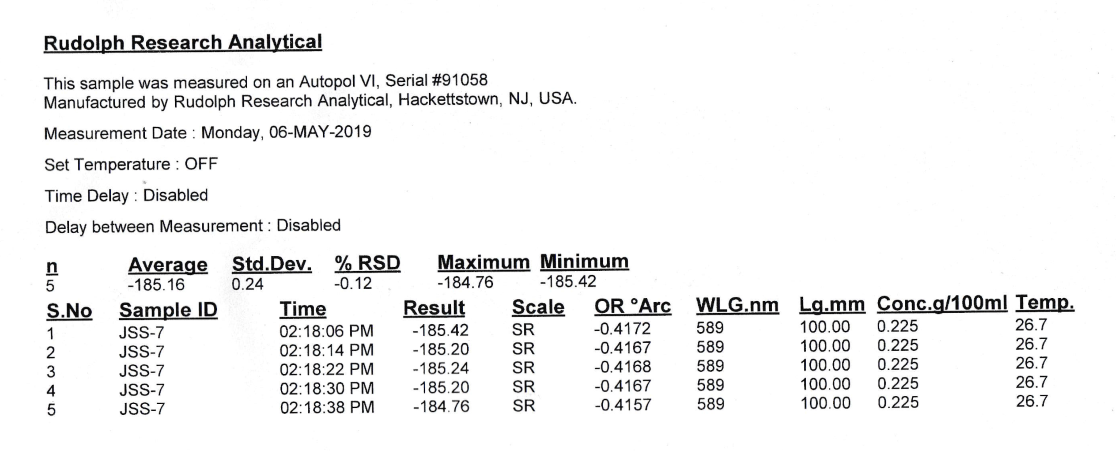
**

**Figure S49**. [*α*]_D_ spectrum of artemyrianin E (**5**) in MeOH

**
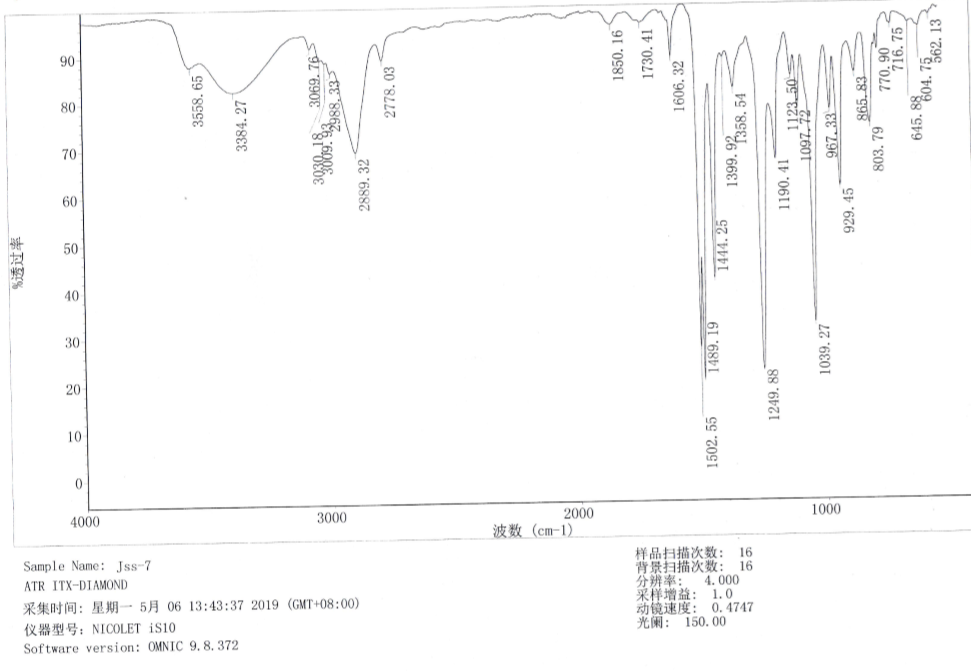
**

**Figure S50**. IR spectrum of artemyrianin E (**5**)

**
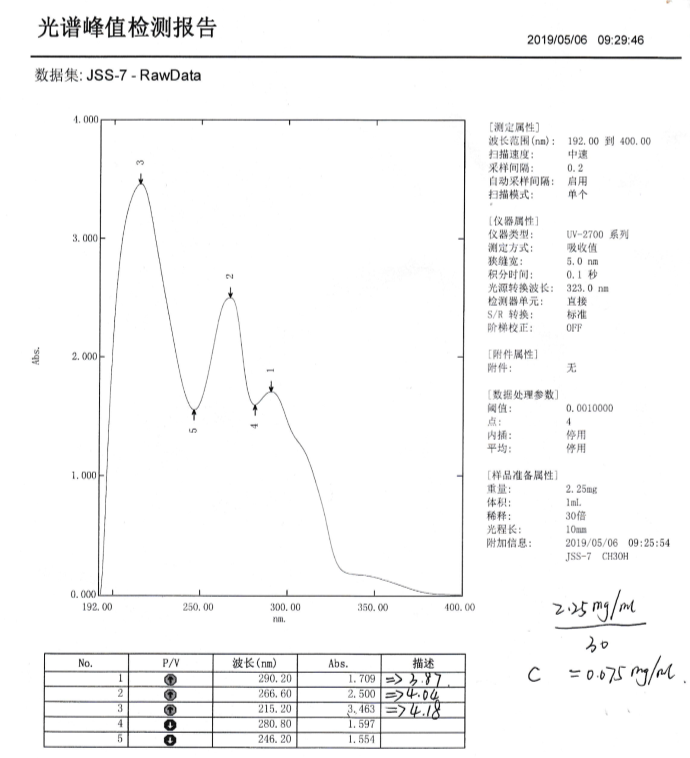
**

**Figure S51**. UV spectrum of artemyrianin E (**5**) in MeOH

_
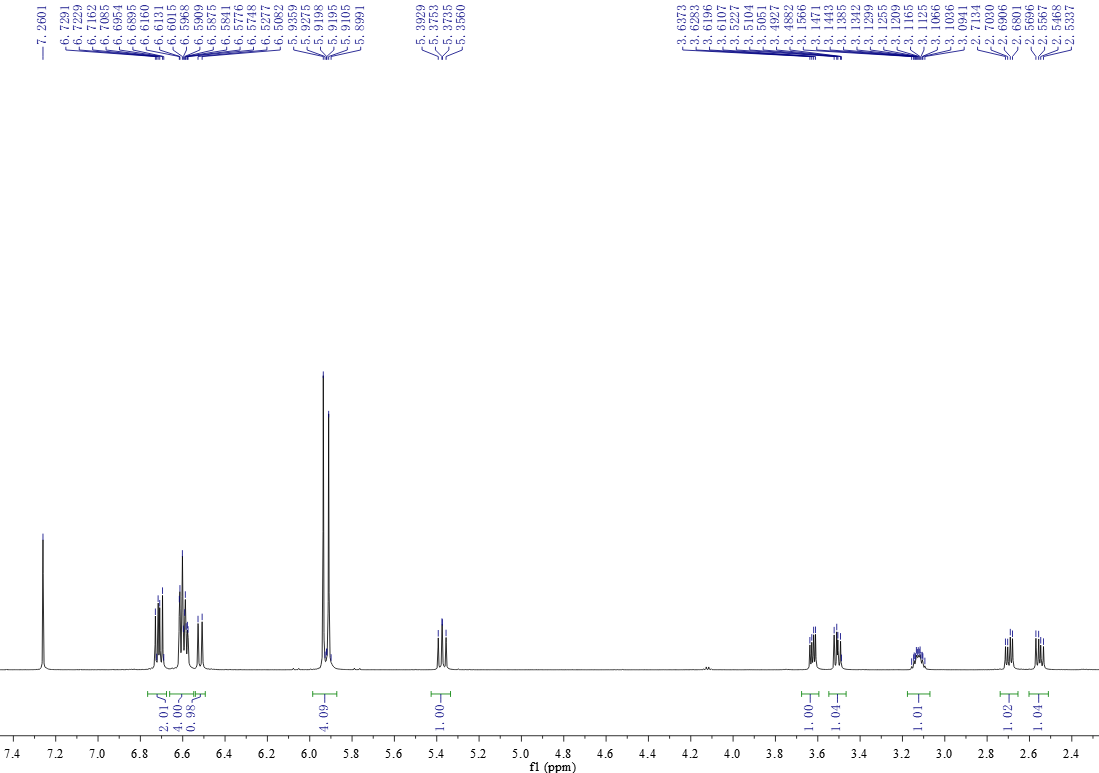
_

**Figure S52**. ^1^H NMR spectrum of artemyrianin F (**6**) recorded in CDCl_3_ at 600 MHz

_
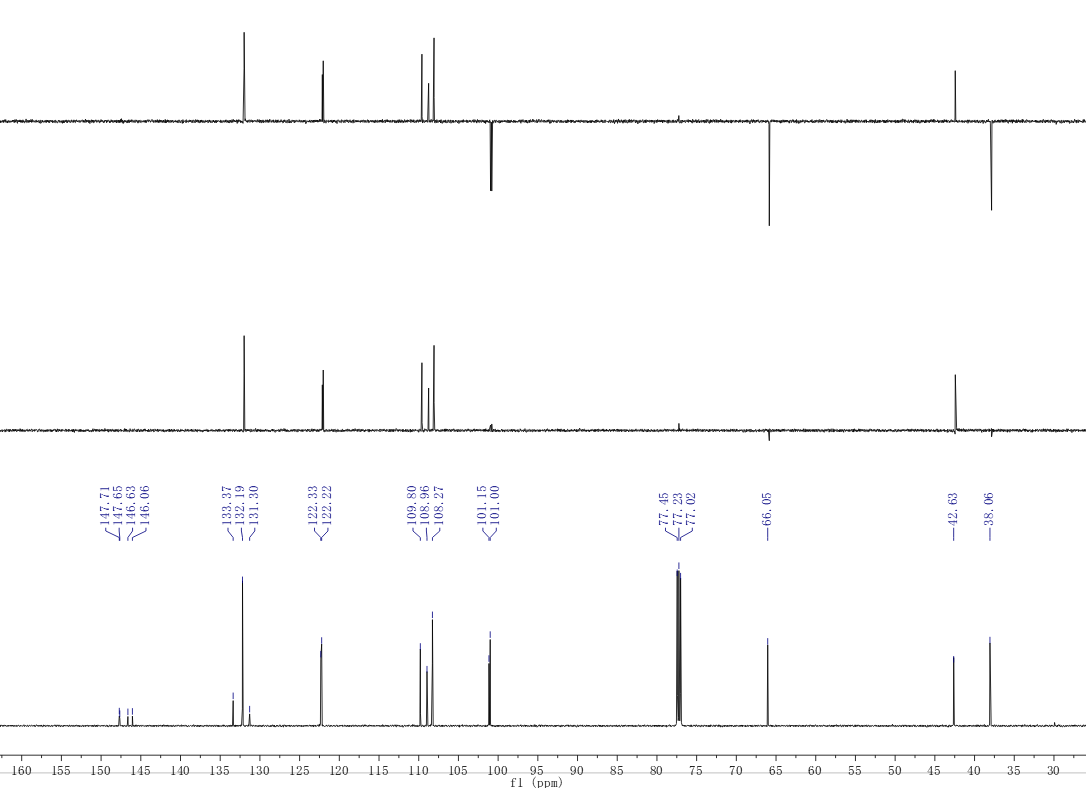
_

**Figure S53**. ^13^C NMR (DEPT) spectrum of artemyrianin F (**6**) recorded in CDCl_3_ at 150 MHz

_
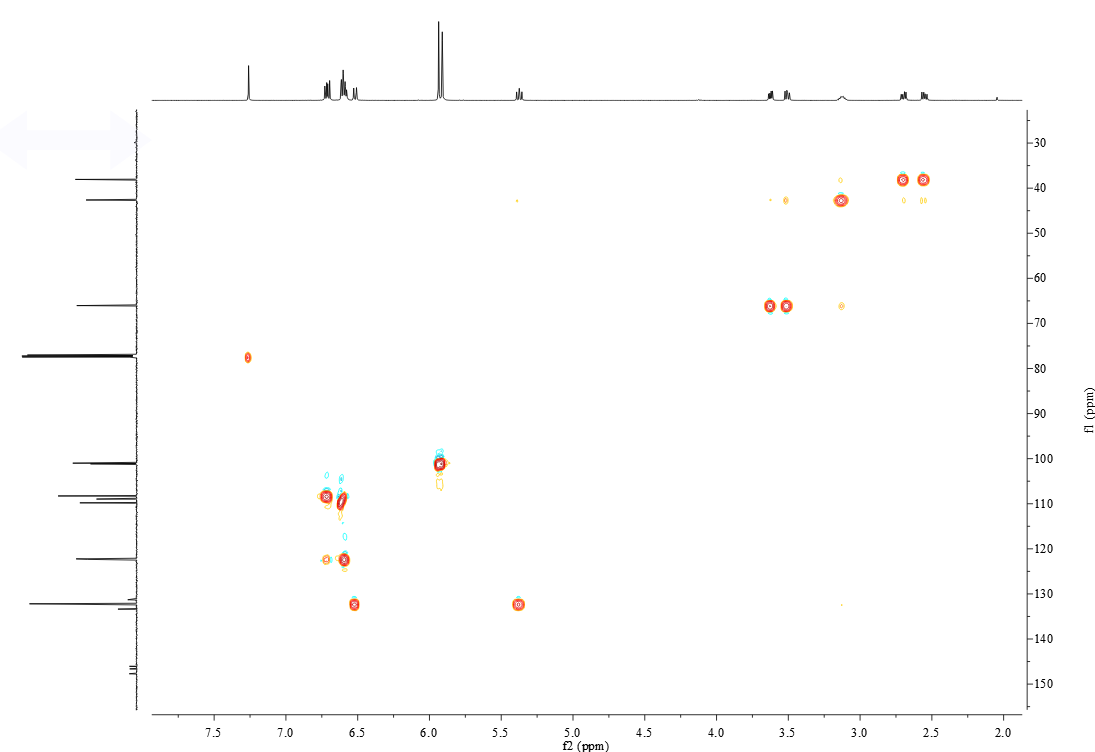
_

**Figure S54**. HSQC spectrum of artemyrianin F (**6**) recorded in CDCl_3_

_
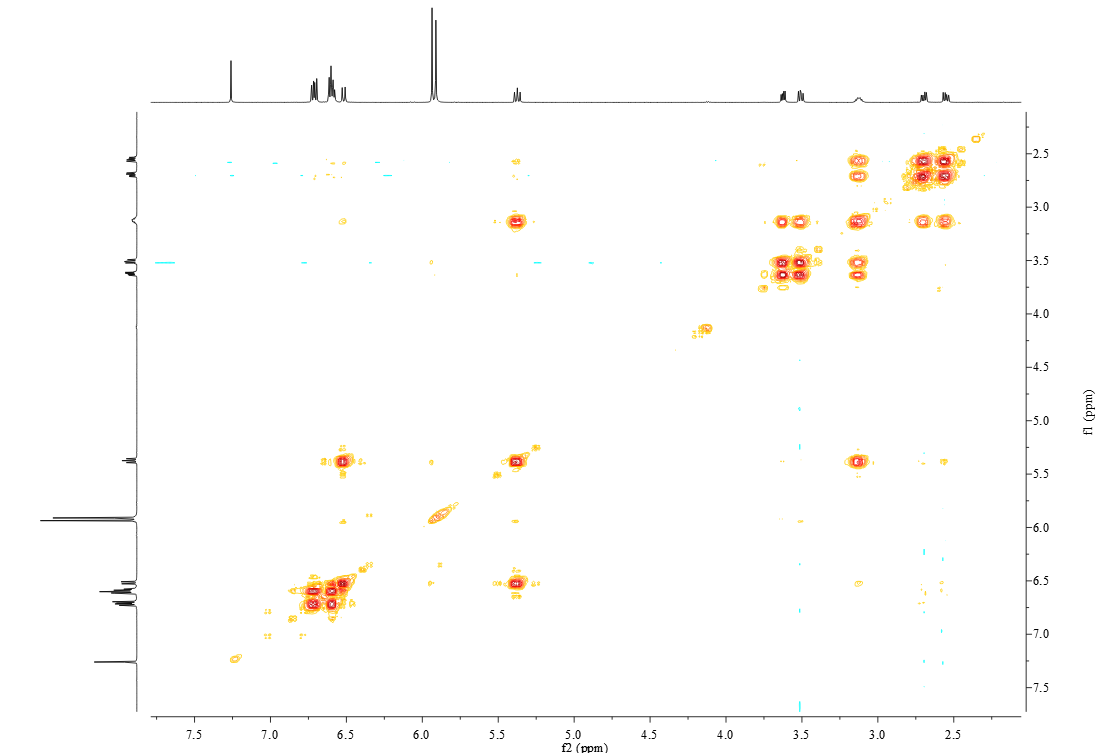
_

**Figure S55**. ^1^H−^1^H COSY spectrum of artemyrianin F (**6**) recorded in CDCl_3_

_
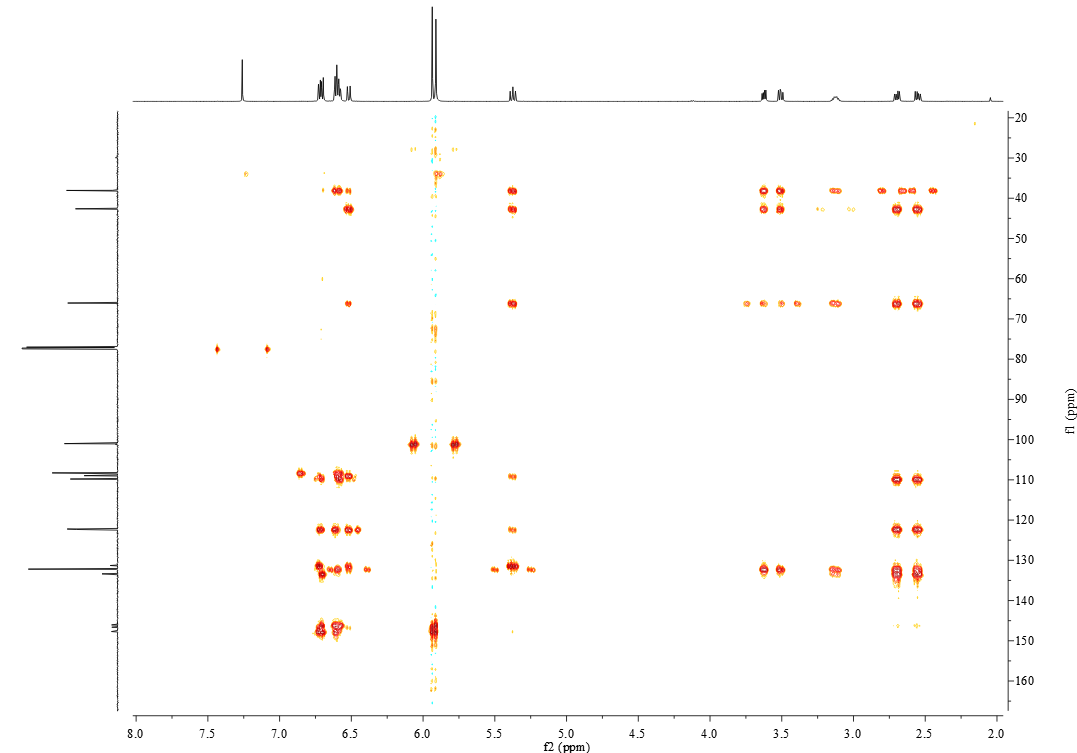
_

**Figure S56**. HMBC spectrum of artemyrianin F (**6**) recorded in CDCl_3_

_
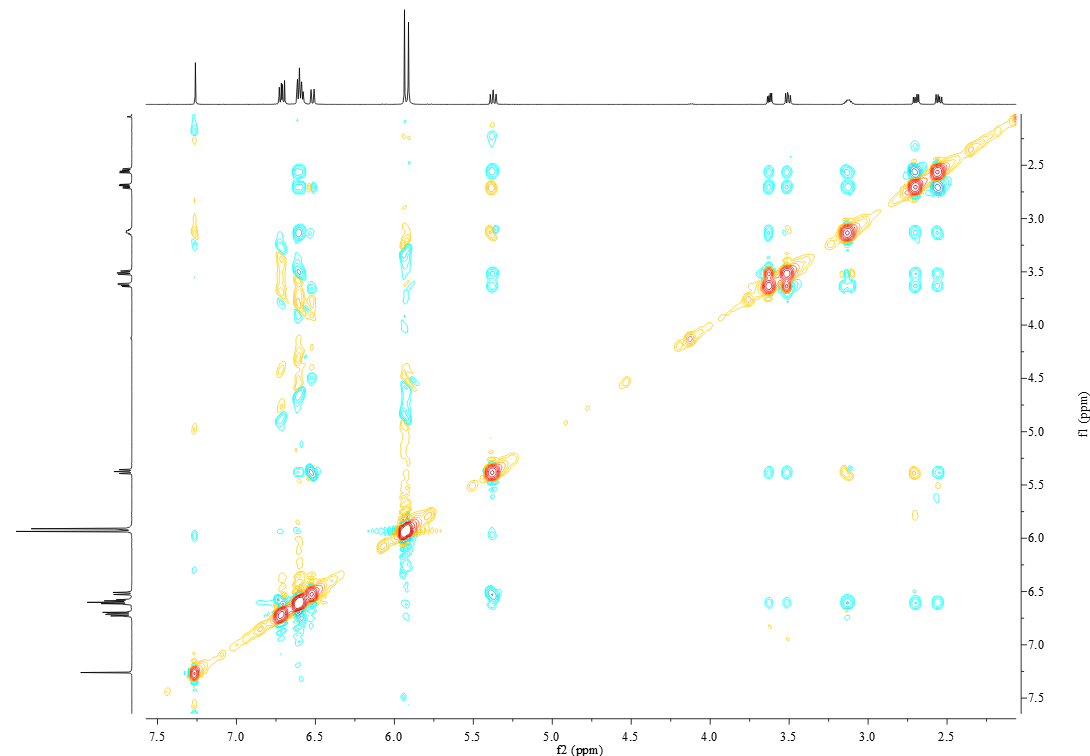
_

**Figure S57**. ROESY spectrum of artemyrianin F (**6**) recorded in CDCl_3_

_
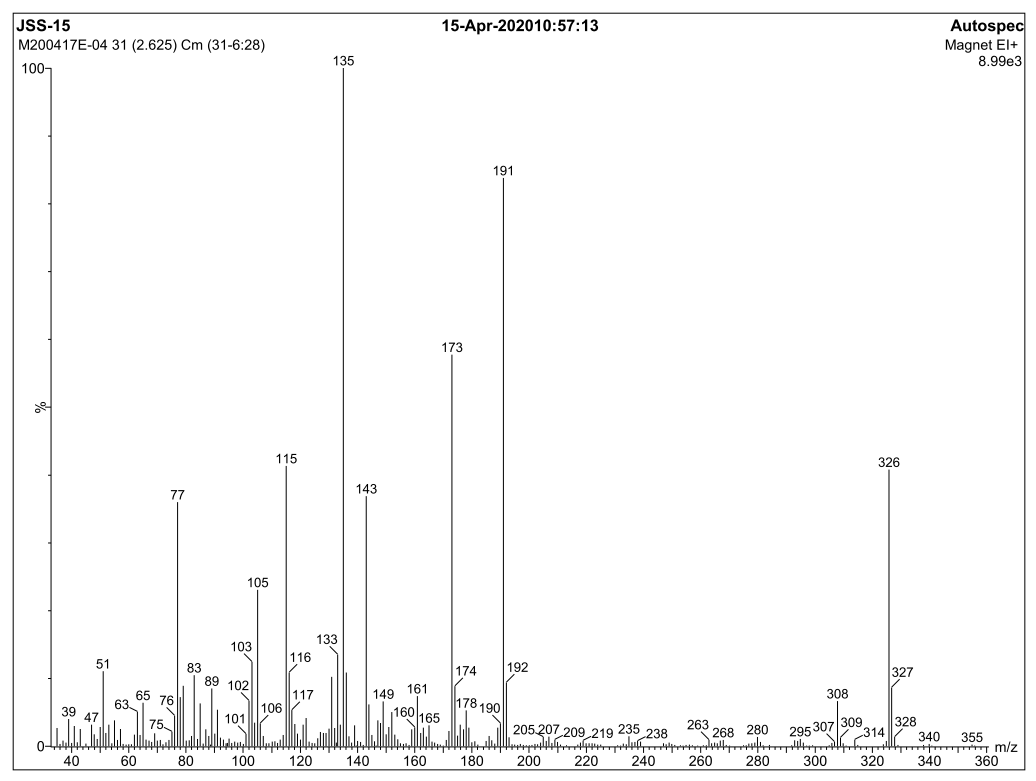
_

**Figure S58**. EIMS spectrum of artemyrianin F (**6**)

_
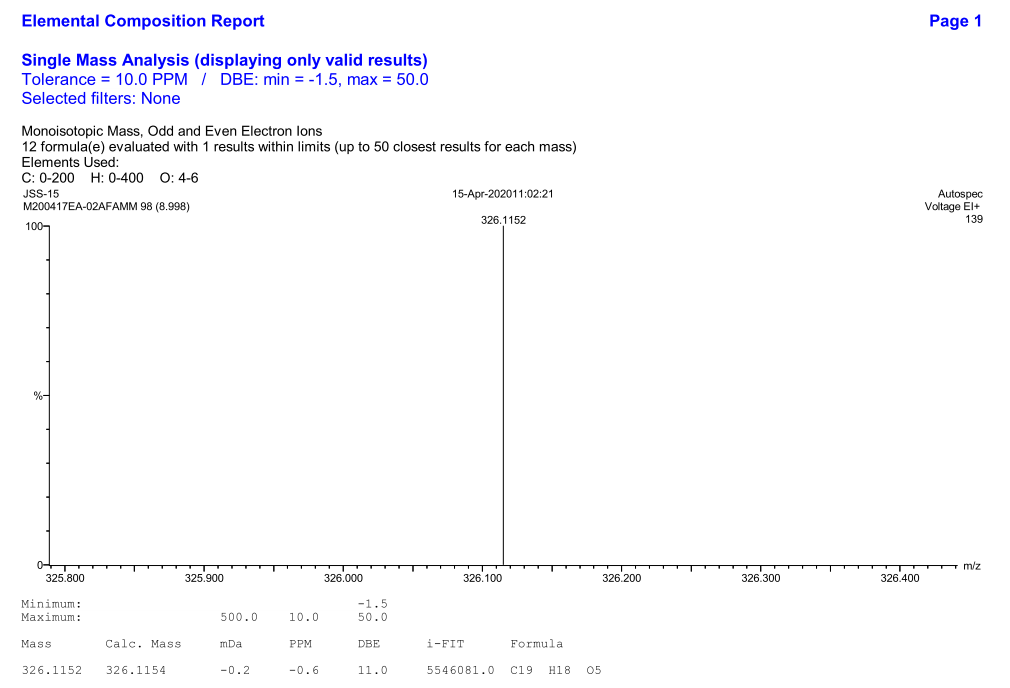
_

**Figure S59**. HREIMS spectrum of artemyrianin F (**6**)

_
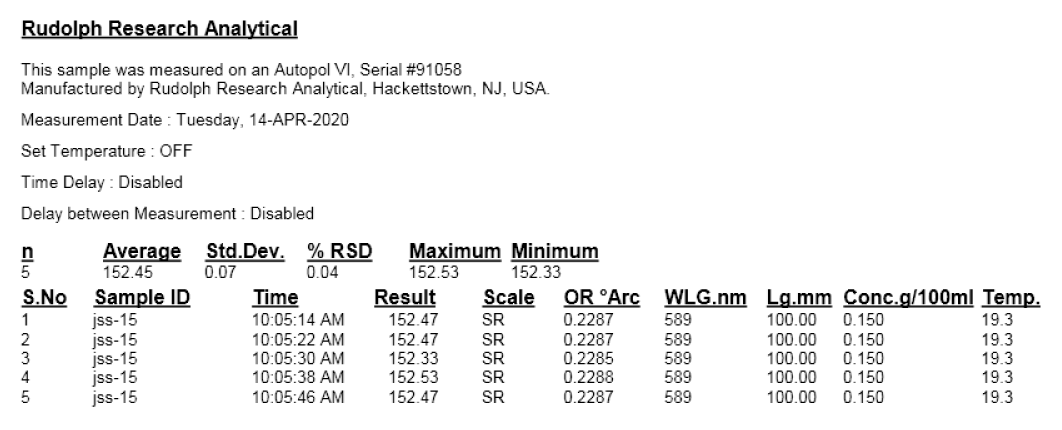
_

**Figure S60**. [*α*]_D_ spectrum of artemyrianin F (**6**) in MeOH

_
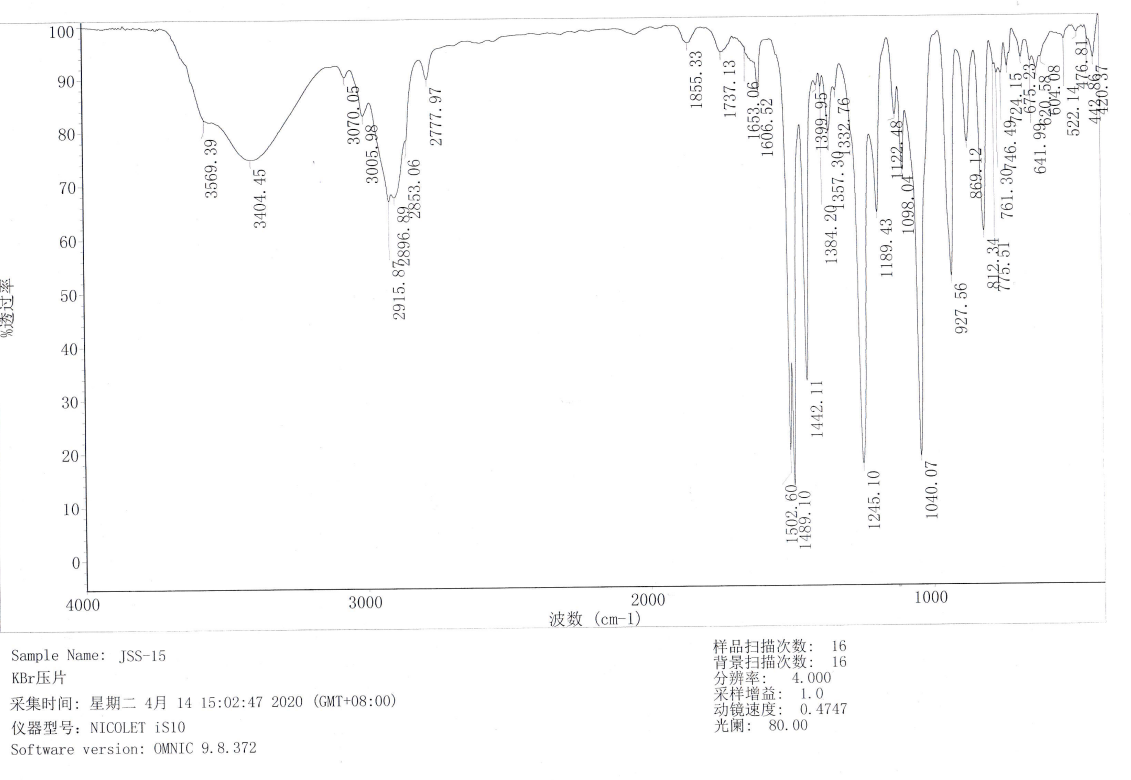
_

**Figure S61**. IR spectrum of artemyrianin F (**6**)


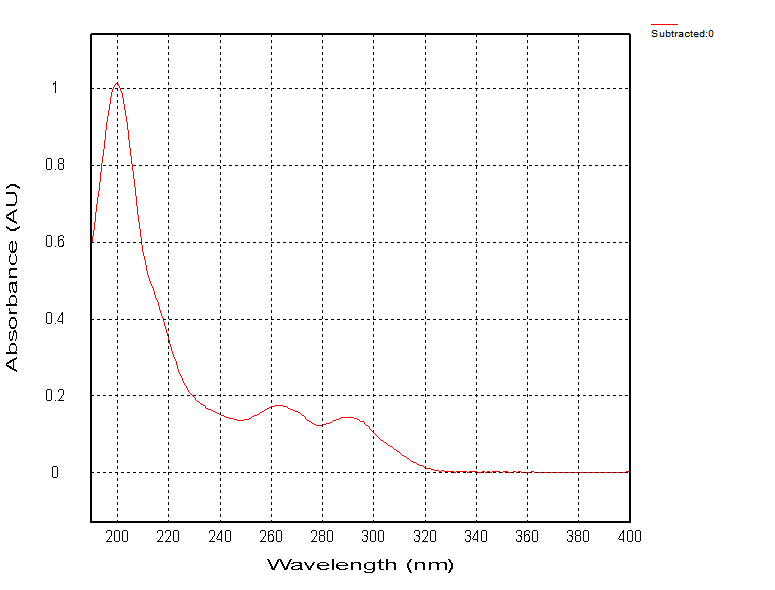


**Figure S62**. UV spectrum of artemyrianin F (**6**) in MeCN

**
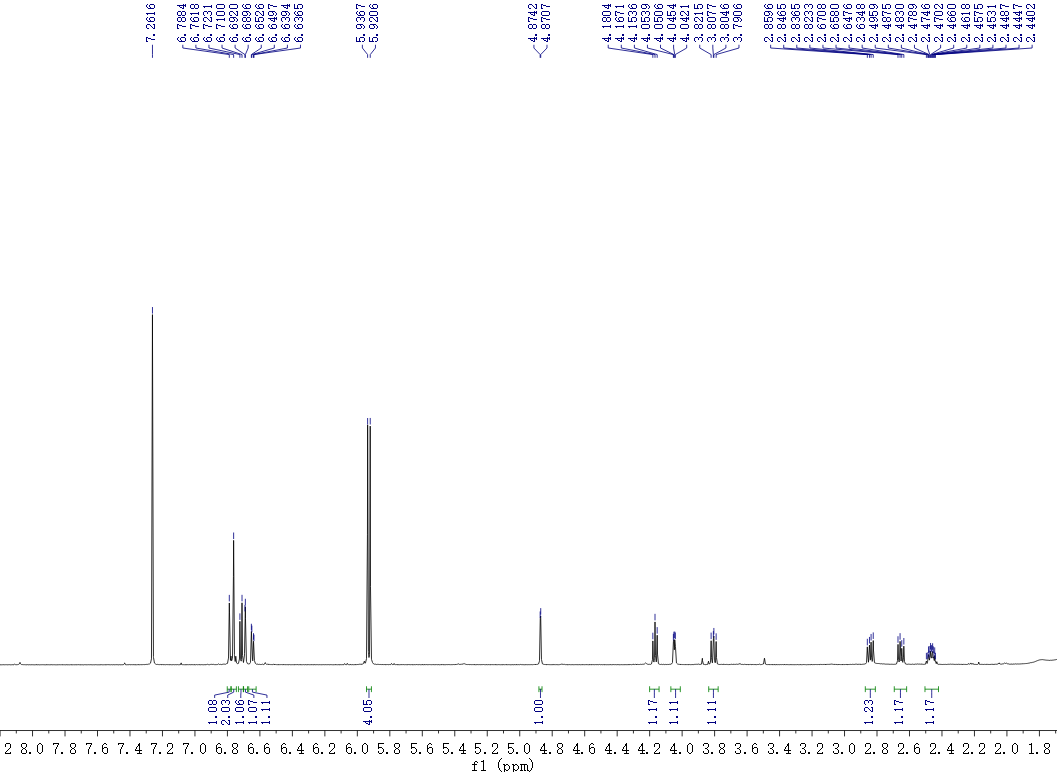
**

**Figure S63**. ^1^H NMR spectrum of artemyrianin G (**7**) recorded in CDCl_3_ at 600 MHz

**
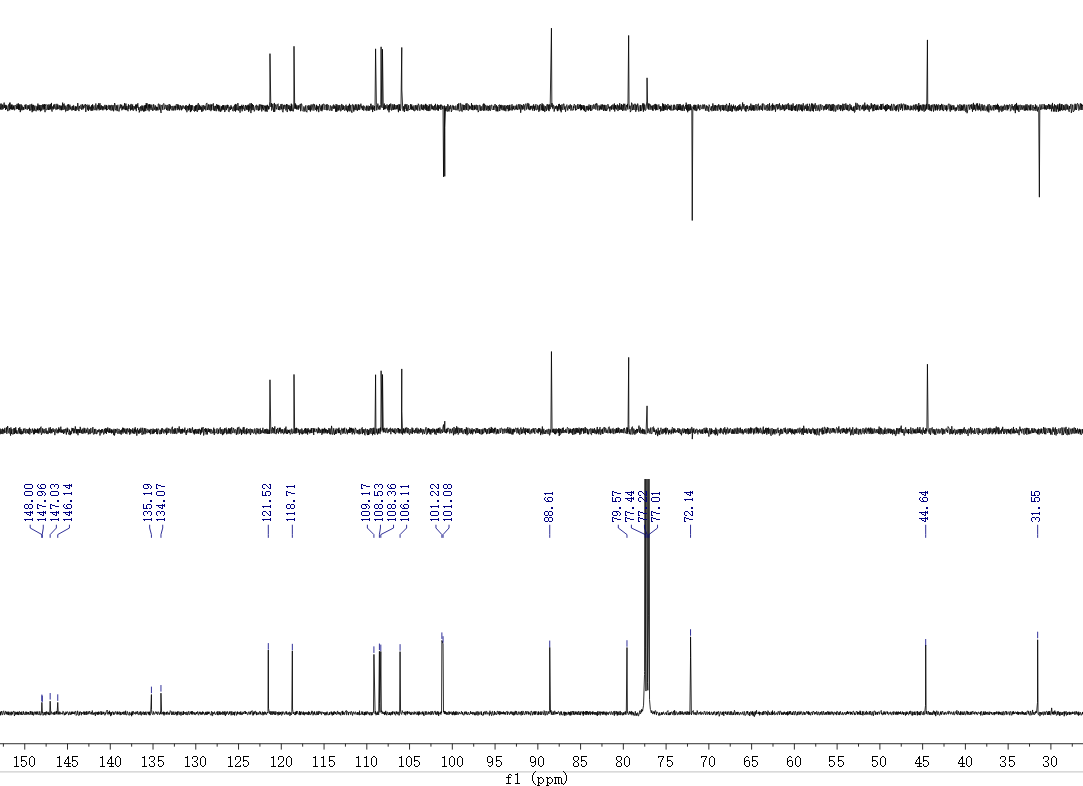
**

**Figure S64**. ^13^C NMR (DEPT) spectrum of artemyrianin G (**7**) recorded in CDCl_3_ at 150 MHz

**
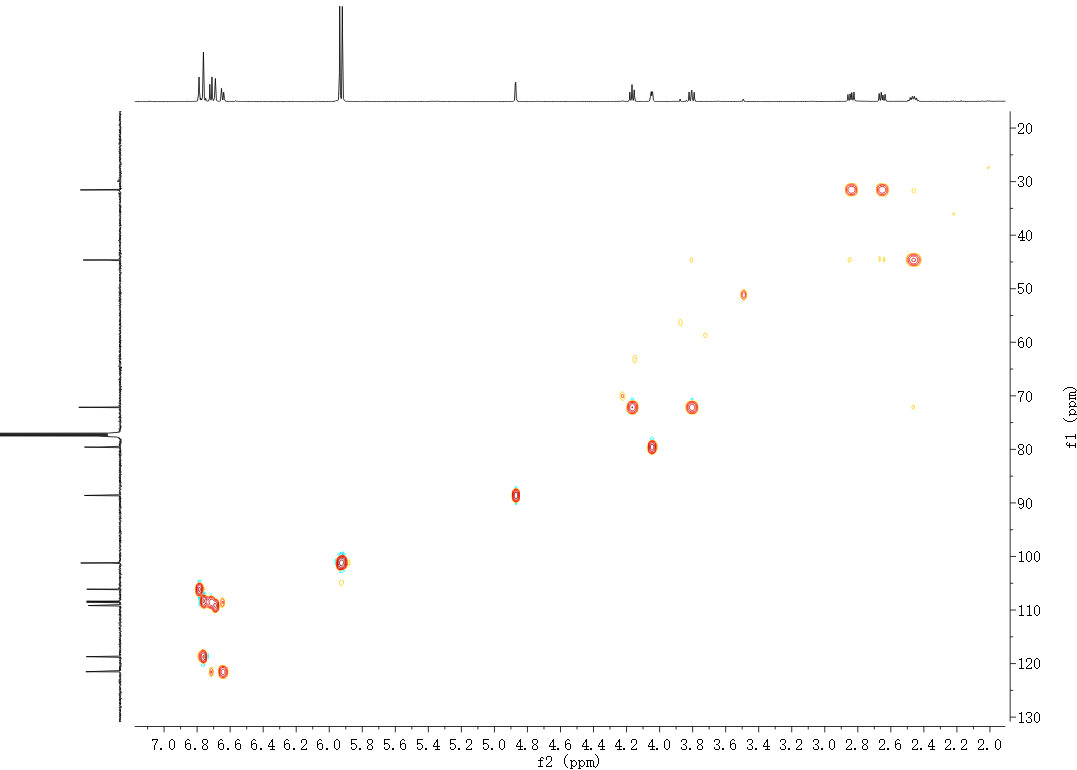
**

**Figure S65**. HSQC spectrum of artemyrianin G (**7**) recorded in CDCl_3_

**
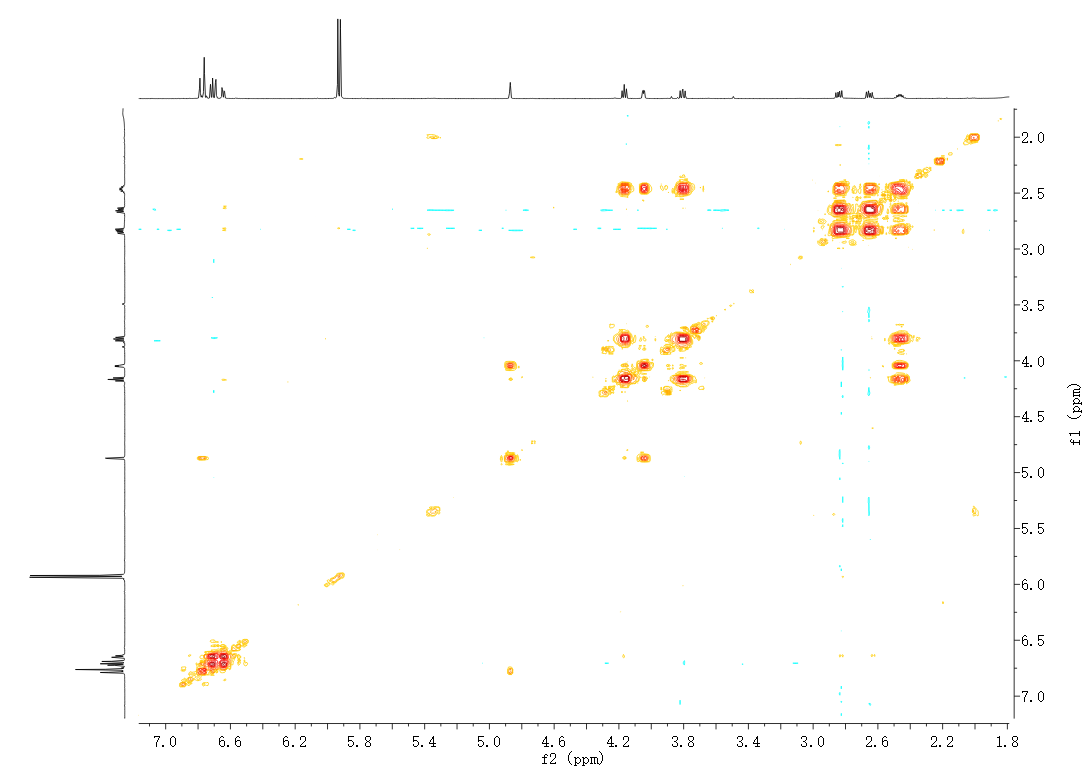
**

**Figure S66**. ^1^H−^1^H COSY spectrum of artemyrianin G (**7**) recorded in CDCl_3_

**
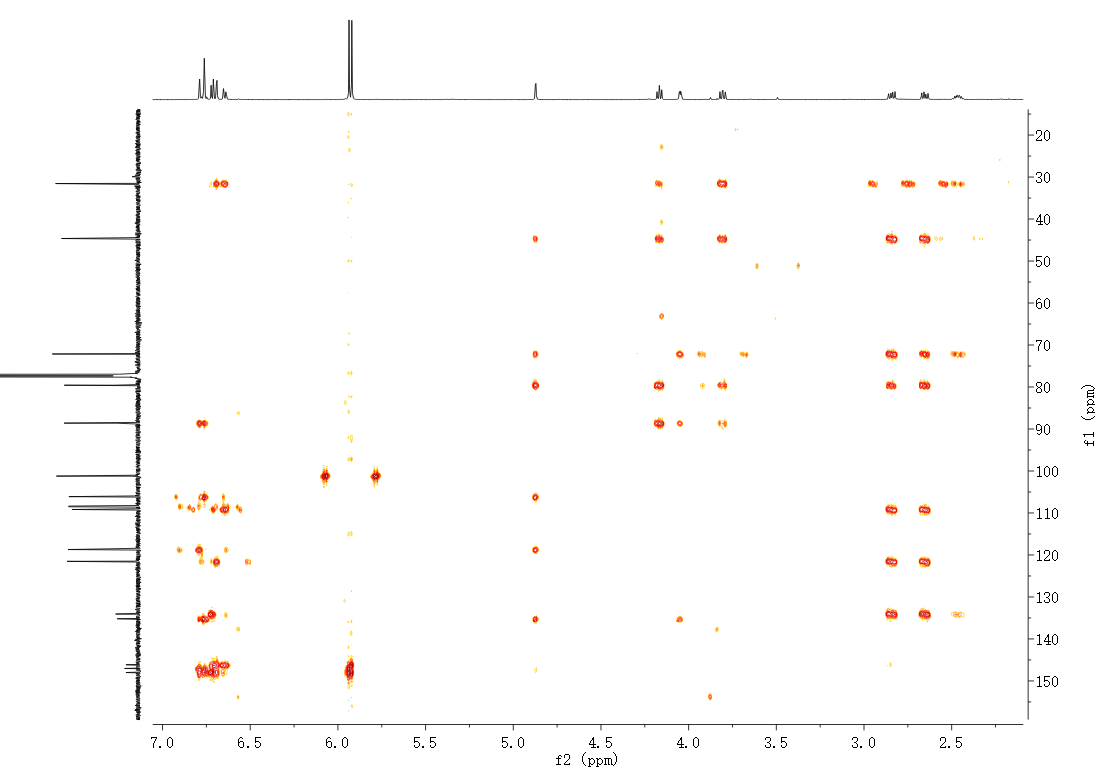
**

**Figure S67**. HMBC spectrum of artemyrianin G (**7**) recorded in CDCl_3_

**
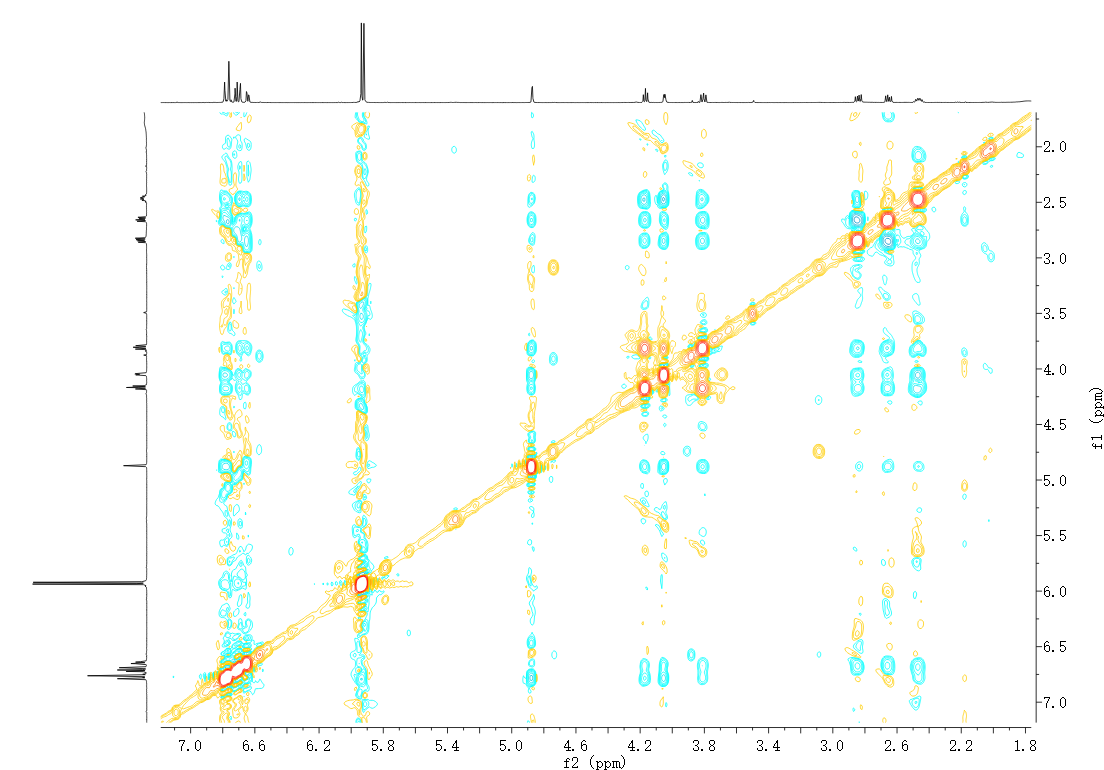
**

**Figure S68**. ROESY spectrum of artemyrianin G (**7**) recorded in CDCl_3_

_
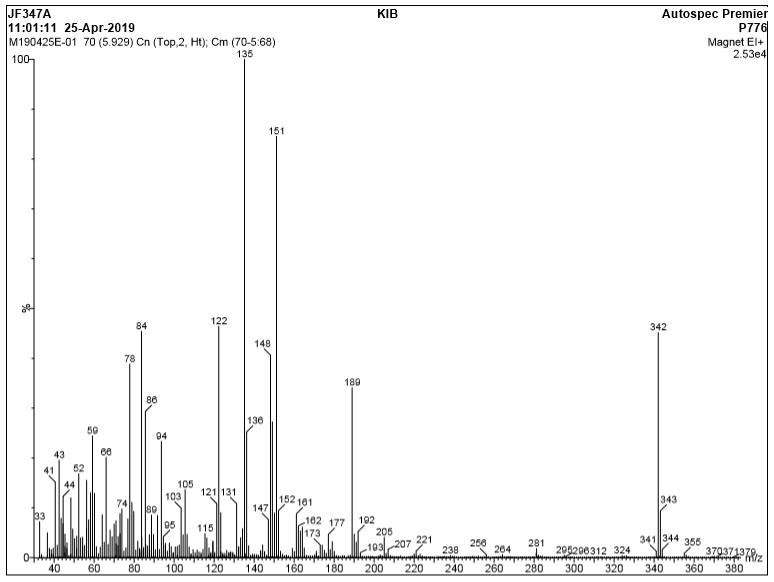
_

**Figure S69**. EIMS spectrum of artemyrianin G (**7**)

**
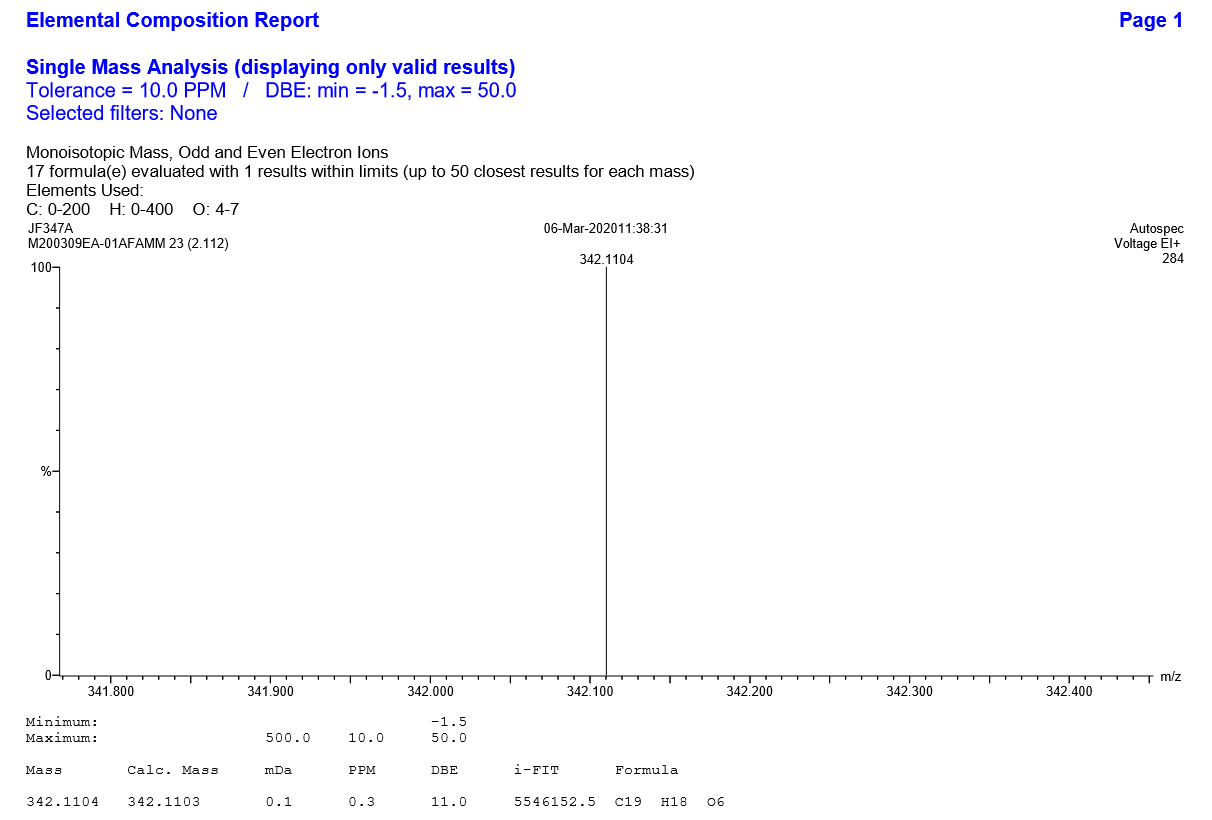
**

**Figure S70**. HREIMS spectrum of artemyrianin G (**7**)

**
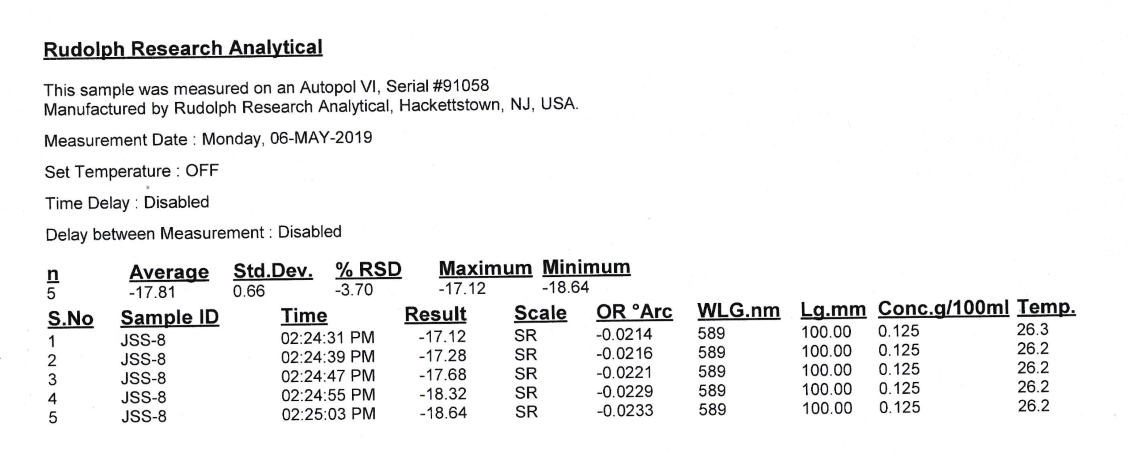
**

**Figure S71**. [*α*]_D_ spectrum of artemyrianin G (**7**) in MeOH

**
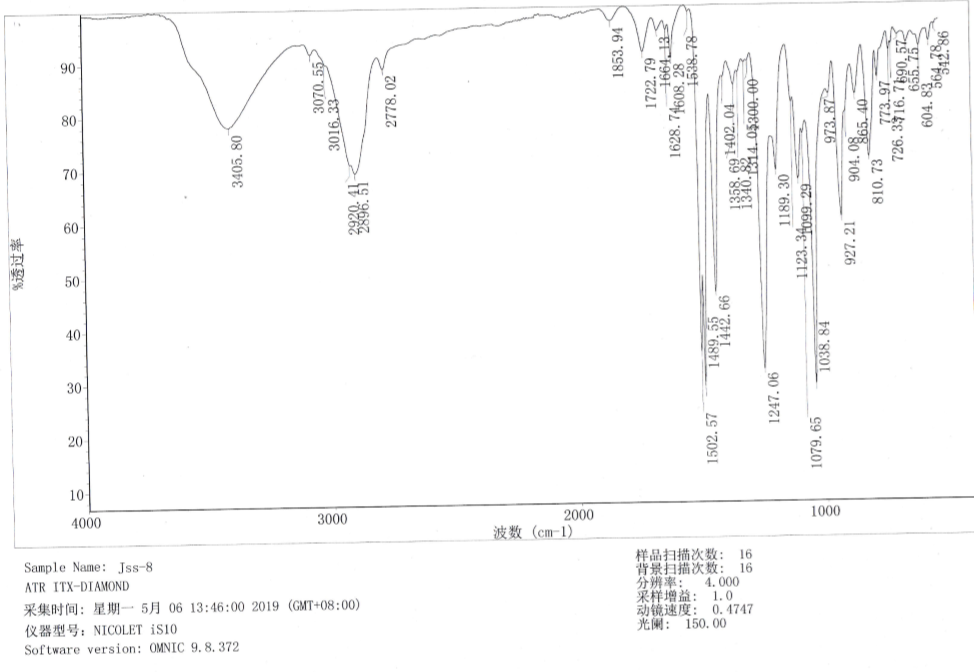
**

**Figure S72**. IR spectrum of artemyrianin G (**7**)

**
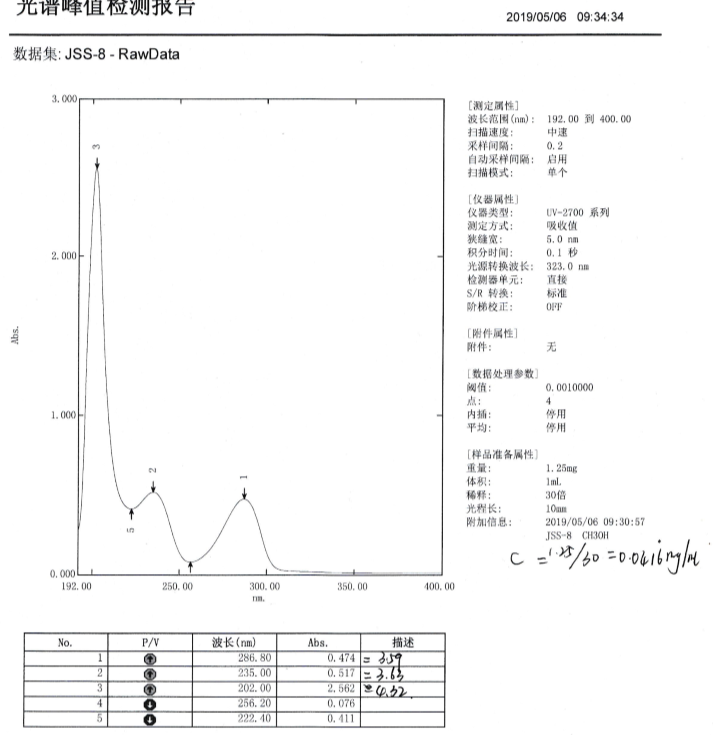
**

**Figure S73**. UV spectrum of artemyrianin G (**7**) in MeOH

**X-ray crystallographic data for artemyrianin A (1)**

C_15_H_20_O_2_, *M* = 232.31, *a* = 6.7424(3) Å, *b* = 6.9858(3) Å, *c* = 7.7309(3) Å, *α* = 105.3440(10)°, *β* = 101.9110(10)°, *γ* = 106.6430(10)°, *V* = 320.37(2) Å^3^, *T* = 100.(2) K, space group *P*1, *Z* = 1, *μ*(Cu *Kα*) = 0.615 mm^-1^, 4616 reflections measured, 2090 independent reflections (*R_int_* = 0.0519). The final *R_1_* values were 0.0484 (*I* > 2*σ*(*I*)). The final *wR*(*F*^2^) values were 0.1244 (*I* > 2*σ*(*I*)). The final *R_1_* values were 0.0485 (all data). The final *wR*(*F*^2^) values were 0.1245 (all data). The goodness of fit on *F*^2^ was 1.040. Flack parameter = 0.18(14).

View of a molecule of **1** with the atom-labelling scheme

Displacement ellipsoids are drawn at the 30% probability level

View of the pack drawing of **1**

Hydrogen-bonds are shown as dashed lines

Crystal data and structure refinement for **1**

Identification code global

Empirical formula C15 H20 O2

Formula weight 232.31

Temperature 100(2) K

Wavelength 1.54178 Å

Crystal system Triclinic

Space group P1

Unit cell dimensions a = 6.7424(3) Å α= 105.3440(10)°.

b = 6.9858(3) Å β= 101.9110(10)°.

c = 7.7309(3) Å γ = 106.6430(10)°.

Volume 320.37(2) Å3

Z 1

Density (calculated) 1.204 Mg/m3

Absorption coefficient 0.615 mm-1

F(000) 126

Crystal size 0.700 x 0.400 x 0.200 mm3

Theta range for data collection 10.95 to 72.29°.

Index ranges -7<=h<=8, -8<=k<=8, -9<=l<=9

Reflections collected 4616

Independent reflections 2090 [R(int) = 0.0519]

Completeness to theta = 72.29° 97.1 %

Absorption correction Semi-empirical from equivalents

Max. and min. transmission 0.89 and 0.58

Refinement method Full-matrix least-squares on F2

Data / restraints / parameters 2090 / 3 / 157

Goodness-of-fit on F2 1.040

Final R indices [I>2sigma(I)] R1 = 0.0484, wR2 = 0.1244

R indices (all data) R1 = 0.0485, wR2 = 0.1245

Absolute structure parameter 0.18(14)

Largest diff. peak and hole 0.352 and -0.250 e.Å-3
